# Feather arrays are patterned by interacting signalling and cell density waves

**DOI:** 10.1371/journal.pbio.3000132

**Published:** 2019-02-21

**Authors:** William K. W. Ho, Lucy Freem, Debiao Zhao, Kevin J. Painter, Thomas E. Woolley, Eamonn A. Gaffney, Michael J. McGrew, Athanasia Tzika, Michel C. Milinkovitch, Pascal Schneider, Armin Drusko, Franziska Matthäus, James D. Glover, Kirsty L. Wells, Jeanette A. Johansson, Megan G. Davey, Helen M. Sang, Michael Clinton, Denis J. Headon

**Affiliations:** 1 Roslin Institute Chicken Embryology, Roslin Institute and Royal (Dick) School of Veterinary Studies, University of Edinburgh, Edinburgh, United Kingdom; 2 School of Mathematical and Computer Sciences, Heriot-Watt University, Edinburgh, United Kingdom; 3 School of Mathematics, Cardiff University, Cathays, Cardiff, United Kingdom; 4 Mathematical Institute, University of Oxford, Oxford, United Kingdom; 5 Department of Genetics and Evolution, University of Geneva, Geneva, Switzerland; 6 Department of Biochemistry, University of Lausanne, Epalinges, Switzerland; 7 FIAS and Faculty of Biological Sciences, University of Frankfurt, Frankfurt, Germany; 8 Cancer Research UK Edinburgh Centre and MRC Human Genetics Unit, Institute of Genetics and Molecular Medicine, Western General Hospital, University of Edinburgh, Edinburgh, United Kingdom; Stanford University School of Medicine, UNITED STATES

## Abstract

Feathers are arranged in a precise pattern in avian skin. They first arise during development in a row along the dorsal midline, with rows of new feather buds added sequentially in a spreading wave. We show that the patterning of feathers relies on coupled fibroblast growth factor (FGF) and bone morphogenetic protein (BMP) signalling together with mesenchymal cell movement, acting in a coordinated reaction-diffusion-taxis system. This periodic patterning system is partly mechanochemical, with mechanical-chemical integration occurring through a positive feedback loop centred on FGF20, which induces cell aggregation, mechanically compressing the epidermis to rapidly intensify *FGF20* expression. The travelling wave of feather formation is imposed by expanding expression of Ectodysplasin A (EDA), which initiates the expression of *FGF20*. The EDA wave spreads across a mesenchymal cell density gradient, triggering pattern formation by lowering the threshold of mesenchymal cells required to begin to form a feather bud. These waves, and the precise arrangement of feather primordia, are lost in the flightless emu and ostrich, though via different developmental routes. The ostrich retains the tract arrangement characteristic of birds in general but lays down feather primordia without a wave, akin to the process of hair follicle formation in mammalian embryos. The embryonic emu skin lacks sufficient cells to enact feather formation, causing failure of tract formation, and instead the entire skin gains feather primordia through a later process. This work shows that a reaction-diffusion-taxis system, integrated with mechanical processes, generates the feather array. In flighted birds, the key role of the EDA/Ectodysplasin A receptor (EDAR) pathway in vertebrate skin patterning has been recast to activate this process in a quasi-1-dimensional manner, imposing highly ordered pattern formation.

## Abstract 2

The highly accurate positioning of feathers in bird embryos is achieved through a spreading wave of the signal EDA, which triggers dense mesenchyme to begin feather patterning. Flightless birds have a more random feather distribution resulting from loss of interaction of the EDA wave with areas of high cell density.

## Author summary

Feathers are a defining feature of birds. In flighted birds, the feathers are arranged in a highly ordered pattern that arises during embryonic development. Feather rudiments are produced in rows, with these rows appearing one after another in a spreading wave. We show that the formation of highly regular feather patterns depends on this wave of formation, with the row-by-row progression triggered by a spreading wave of the signal Ectodysplasin A (EDA). The EDA signal cooperates with a spreading wave of cellular density to spark the formation of regularly spaced cell aggregates, each of which serves as the precursor to a feather. The EDA wave and the cell density wave are themselves independent of feather formation, as they can be detected travelling across the embryonic skin even when feather formation is impaired. In the flightless birds examined these waves are missing and a highly ordered feather pattern is not produced. However, the manner of wave loss differs in different species, with ostrich embryos lacking the wave of EDA, whereas the emu lacks sufficient skin cells at the appropriate developmental stage to make precisely organised feather rudiments.

## Introduction

Feathers evolved before birds but today are their primary diagnostic character. Feathers are arranged in clusters called tracts [1], apparent as well-defined feather-bearing areas of skin at specific body sites. Each tract is a contiguous set of feathers initially laid out with a regular spacing between neighbouring feathers. This typically results in every feather within the developing tract having six nearest neighbours arranged about it, the entire pattern forming a lattice of hexagons. Such a hexagonal arrangement of neighbours is the most efficient packing of a 2-dimensional space, arising through the closest possible positioning of elements when each projects a circular inhibitory influence preventing the placing of new elements nearby [2,3].

**Fig 1:**
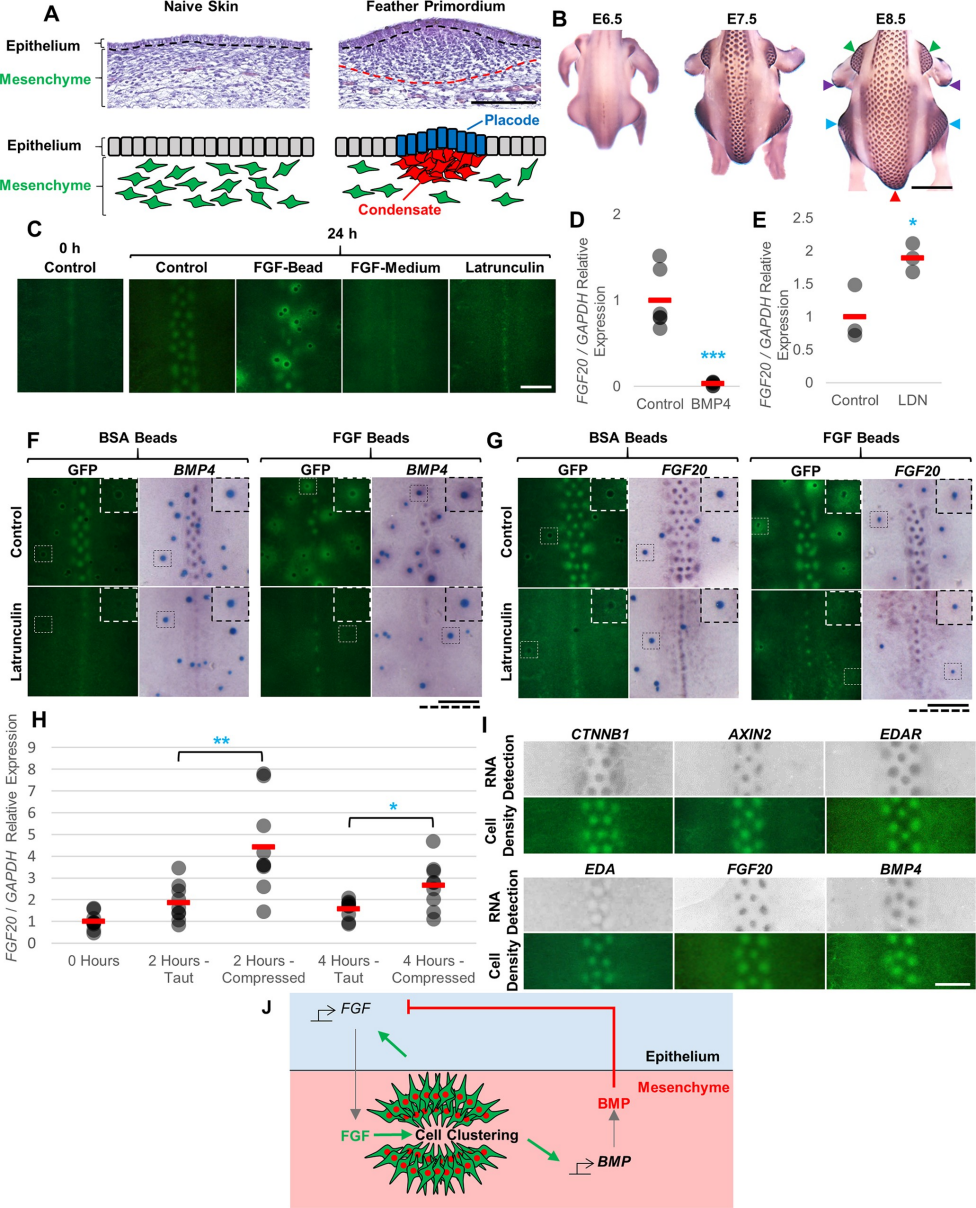
Molecular and cellular interactions in feather patterning. (A) Sections of embryonic skin showing cell distribution before and after feather primordium formation. Cell nuclei are stained purple. Black dashed lines indicate the epithelial-mesenchymal boundary. Schematic below. Scale bar: 100 μm. (B) Time series of feather primordium formation in chicken embryos from E6.5 visualised by detection of *CTNNB1* expression by RNA in situ hybridisation. Arrowheads indicate the discrete tracts that are visible in this orientation (red: dorsal, green: humeral, blue: femoral, purple: alar). Scale bar: 2.5 mm. (C) Skin explants from E6.5 CAG-GFP embryos cultured for 0 or 24 hours. Cell condensates appear as green spots. Cell aggregation occurs only when FGF sources are localised to beads (apparent here as nonfluorescent black dots). Treatment with 1 μg/ml FGF9 or 150 ng/ml Latrunculin A, an inhibitor of cell movement, abolishes skin patterning. Scale bar: 1 mm. (D) and (E) qRT-PCR assessing *FGF20* expression in E6.5 skin explants cultured in the presence of (D) 500 ng/ml BMP4 or (E) 10 μM LDN193189 (an inhibitor of BMP signalling) for 5 hours. Filled circles indicate individual data points; red bars indicate the mean. Statistical significance from control was calculated using a Student *t* test (*p < 0.05, ***p < 0.001). (F) and (G) Skin explants from E6.5 CAG-GFP embryos cultured for 18 hours with BSA- or FGF9-coated beads (blue coloured) in the presence or absence of 150 ng/ml Latrunculin A in the culture medium. Detection is of (F) *BMP4* or (G) *FGF20* expression (purple-coloured signal). Cell aggregation at FGF beads is accompanied by induction of *FGF20* and *BMP4* expression. Insets show enlargement of area around a single bead and corresponding dotted bars for scale. Scale bars: 1 mm. (H) qRT-PCR determination of *FGF20* expression levels in E6.5 skin explants freshly dissected from embryos and explants cultured for 2 or 4 hours either on filters (taut) or free-floating in culture medium (compressed). Statistical significance from respective controls was calculated using a Student *t* test (*p < 0.05, **p < 0.01). (I) Skin from E6.5 CAG-GFP embryos was cultured for 24 hours and imaged to detect GFP, and then *CTNNB1*, *AXIN2*, *EDAR*, *EDA*, *FGF20*, and *BMP4* expression was detected in the same sample by in situ hybridisation. Regions of high cell density (i.e., intense GFP signal) completely overlap focal expression of molecular markers of primordium formation. Scale bar: 500 μm. (J) Schematic of the molecular and cellular processes involved in feather induction. Green arrows indicate stimulation and red bars inhibition. The numerical values for D, E, and H can be found in S1 Data. BMP, bone morphogenetic protein; BSA, bovine serum albumin; E, embryonic day; FGF, fibroblast growth factor; GFP, green fluorescent protein; qRT-PCR, quantitative reverse transcription PCR.

The skin is a composite tissue, with its epithelium, the epidermis, attached to the underlying mesenchyme, the dermis. The epithelium arises from the embryonic ectoderm, whereas the dermis has distinct embryonic sources depending on anatomical location. Intercellular junctions fix epidermal cells together to form a sheet, limiting their independent movement, whereas the individual cells of the dermis are free to move through the matrix that they secrete. In the embryonic skin, the initiation of feather formation begins with the condensation of dermal mesenchymal cells beneath an epidermal placode (Fig 1A). Both structures are identifiable by the tighter packing of their cells and the alteration of expression of a suite of genes. This combined structure, the feather primordium, then grows into a filament within a follicle, branches internally, and produces a down feather by hatch. In the chicken embryo, the process of laying out the periodic arrangement of feathers begins at precisely stereotyped anatomical sites at embryonic day (E)6.5, laying down a row of feathers as the initiation of each tract. New rows are added progressively in parallel to existing ones such that a wave of feather formation sweeps laterally across the skin (Fig 1B, S1A Fig). These spreading waves terminate before colliding, yielding embryonic tracts containing hexagonal arrays of feather primordia and bare spaces between the tracts. In the chicken, these spaces remain largely unfeathered, but in other species they can become filled with small down feathers.

Prior to the appearance of the dermal mesenchymal cell condensates that represent the rudiment of individual feathers, the prospective feather tracts are identifiable by their high mesenchymal cell density, contrasting with the lower cell density of the future unfeathered skin. Both epithelium and mesenchyme are essential for periodic patterning and feather primordium formation, and this process fails to initiate if either tissue is maintained in isolation [4]. Separations and recombinations of epithelium and mesenchyme from different skin regions have shown that low-cell-density mesenchyme is not capable of supporting feather development, whereas high-density mesenchyme undergoes feather formation together with an overlying epidermis taken from any embryonic site [4–10].

The rapid emergence of a regular repeating pattern of feather rudiments has long served as a model for epithelial-mesenchymal self-organisation in development and has attracted much experimental and theoretical effort to define its mechanistic basis. By distorting skin shape and area, Davidson [11] showed that the positions of feathers are not predetermined during embryonic development and that the underlying patterning mechanism does not rely on a mechanism for counting of feather rows, as the number of rows formed is dependent upon skin area. Among the theories proposed to explain skin pattern formation, one set focuses on the potential for cell behaviour to enact pattern formation directly, particularly through physical means such as traction via construction of and migration along a collagen lattice [12–15]. Shyer and colleagues [16] have coupled such ideas with molecular events by presenting evidence that local contraction of skin arising from mesenchymal tension initiates feather primordium formation through a mechanically triggered stimulation of epidermal β-catenin activity. In general, these ideas centre on motile cell behaviour directly driving tissue patterning, without recourse to a template of altered cell states with differential gene expression.

An alternative set of theories to explain periodic patterning suggests that interacting intercellular signals first define the pattern that will form and that this pattern template then alters cell behaviour locally to enact morphological change. Such Turing or reaction-diffusion systems [17–20] rely on patterned gene expression as an intermediate stage in pattern formation, distinct from the direct cell aggregation systems produced by mesenchymal cell movement, traction, or compression. In these template-directed systems, cells attain information on their relative locations using diffusible signals, defining activated and nonactivated regions [21]. This requires that cell movement over the time course of pattern definition be limited so that cells acquire information on their relative position and thus appropriate differentiation route relative to the states of nearby and distant cells, without such information being scrambled through rapid changes in cell location.

Alongside the proposal of these overarching patterning mechanisms, molecular approaches have identified several regulators of feather development. The bone morphogenetic protein (BMP) class of extracellular signals have been identified as inhibitors [22–24] and WNT [25] and fibroblast growth factor (FGF) [26–29] as stimulators of feather primordium formation. The activity of the extracellular signal Ectodysplasin A (EDA) and EDA receptor (EDAR) have been found to be crucial for hair follicle, tooth, and scale formation in fish, lizards, and mammals [30,31]. In chicken, targeted experiments found that locally forced stimulation of the EDAR pathway can induce formation of extra feather buds at the margins of expanding tracts [32]. No loss-of-function mutation affecting a component of the EDAR pathway has been identified in birds.

More recently, understanding of the genetic basis of natural variation in feather patterning has further supported the defining role of the BMP and FGF pathways in this process. The Naked neck trait, which is characterised by reduced feathering on the body and an absence of feathers on the neck, was found to be caused by a regulatory mutation leading to increased *BMP12* production in the embryonic skin. In this study, simulations of a reaction-diffusion signalling process, with BMPs acting as inhibitor, were sufficient to interpret the experimentally induced pattern changes observed [33].

The scaleless mutant [34] lacks feathers and foot scales because of a developmental failure of mesenchymal condensate and epidermal placode formation [28,35–39]. The trait is caused by a loss-of-function mutation in *FGF20*, a gene expressed strictly in the epidermis [29], agreeing with older literature showing that the defect in scaleless mutant skin lies in the epidermis [36]. This mutant line has been used extensively to study the interactions between embryonic dermis and epidermis, and a wealth of results obtained underscore a critical role for the epithelium in the initiation of feather development [36,38,40,41].

Here, we investigate the integration of intercellular signalling, mesenchymal cell movement and density, and mechanical processes in driving feather pattern formation. We identify EDA/EDAR signalling as the critical signal driving the wave of patterning and spreading across and interacting with a gradient of mesenchymal cell density. These waves are lost, though in different ways, in the flightless ostrich and emu, leading to loss of precise hexagonal periodicity of their feather arrangements.

## Results

### An integrated cell signalling, cell aggregation, and mechanical process breaks symmetry to achieve periodic patterning of feathers

We first aimed to determine the mechanism underlying the local spatial patterning of feathers. Based on genetic evidence for their importance in feather patterning [29,33], we began by investigating the relationship between FGFs and BMPs, and their control of cell aggregation, in developing chicken dorsal skin. To define the role of FGF, we treated skin explant cultures with FGF9 (a member of the FGF9/16/20 subfamily with more stable activity than FGF20) [42,43] to assess its effect on cell behaviour and feather patterning. We used embryonic skin from the CAG-GFP transgenic line [44], which expresses green fluorescent protein (GFP) in all cells, to reveal feather primordia as sites of high cell density. Skin explants were cultured and imaged after perturbation of FGF signalling. Treatment of explants with FGF protein yielded differing results depending on the protein’s spatial availability. Local exposure via delivery from an FGF-soaked bead resulted in mesenchymal cell aggregation at the FGF source and disruption to the endogenous condensate pattern, whereas ubiquitous exposure through FGF incorporation into the culture medium inhibited all primordium formation (Fig 1C). Inhibition of FGF signalling, via treatment of explants with SU5402, further demonstrated the importance of FGF signalling in condensate formation (S1B Fig). These findings suggest that FGF protein acts as a chemoattractant inducing mesenchymal cell clustering at its local sources, but ubiquitous exposure blinds cells to endogenous sources. Consistent with a requirement for cell movement in feather condensate formation, Latrunculin A, an inhibitor of actin polymerisation [45] and thus cell movement, also suppresses feather pattern formation (Fig 1C).

Application of BMP4 or other BMP family members reduced the number of feather primordium rows formed in cultured explants in a dose-dependent manner (S1C Fig and S1D Fig). Pharmacological inhibition of BMP signalling using LDN193189 resulted in broad areas of skin assuming primordium identity, with loss of the interprimordium interval (S1E Fig and S1F Fig). Quantitative reverse transcription PCR (qRT-PCR) analysis detected suppression of *FGF20* expression after 5 hours of BMP4 treatment, whereas inhibition of BMP signalling led to rapid elevation of *FGF20* levels, showing that BMP rapidly negatively regulates *FGF20* expression (Fig 1D and Fig 1E). Cotreatment of explants with FGF-soaked beads and ubiquitous BMP4 inhibited endogenous primordium formation but did not prevent cell aggregation at applied FGF protein sources (S1G Fig). Thus, BMP signalling inhibits FGF production but does not abolish cell coalescence at FGF sources.

At the core of periodic patterning systems based on intercellular signalling is the phenomenon of self-activation coupled to lateral inhibition [20,21]. If FGF and BMP interactions constitute such a system in feather patterning, then FGF signals should stimulate BMP expression. However, we were unable to detect activation of expression of BMP family members (*BMP2*/*4*/*7*) after FGF treatment (S2A Fig), suggesting that these genes are not regulated directly by FGF stimulation. Thus, considering FGF and BMP interactions, we are unable to construct a simple reaction-diffusion system capable of producing a periodic pattern de novo in chicken skin.

We next explored the potential for the process of local cell condensation itself to induce expression of patterning genes. We observed induction of *BMP4* and *FGF20* expression at sites of cell aggregation induced by applied FGF beads (Fig 1F and Fig 1G). Coadministration of Latrunculin A, to suppress cell movement and thus aggregation, prevented induction of gene expression, demonstrating that cell aggregation, rather than simple exposure to FGF, underlies induction of *BMP4* and *FGF20* (Fig 1F and Fig 1G). Thus, cell aggregation is both controlled by and stimulates production of these signals required for periodic pattern formation.

Shyer and colleagues recently reported that contraction of embryonic chicken skin, achieved by culturing the skin without a support, is driven by mesenchymal tension [16]. Such contraction causes rapid displacement of β-catenin protein (encoded by the *CTNNB1* gene) from epithelial cell junctions and permits nuclear entry of this protein, mimicking the effect of canonical WNT signalling. We assessed whether tissue contraction as performed by Shyer and colleagues [16] might lead to activation of *FGF20* expression by culturing chicken skin either unattached and thus free to contract or pinned to filters and thus under tension, and assessing subsequent changes in *FGF20* expression. At both time points assessed (2 and 4 hours), *FGF20* expression was markedly elevated in the contracted tissue, supporting a role for cell aggregation in rapid activation of *FGF20* production (Fig 1H). No other genes that we analysed (*AXIN2*, *BMP2*, *BMP4*, *EDA*, and *EDAR*) were observed to alter expression in these conditions (S2B Fig).

These results point to the operation of an integrated reaction-diffusion-taxis system in feather patterning. If such a system underlies patterning, then the appearance of cell aggregates should neither precede nor follow local changes in gene expression, but both features should emerge synchronously. To assess the relative timing of formation of mesenchymal cell aggregates and the activation of feather primordium gene expression, we assessed both cell distribution and gene expression in the same cultured skin samples (Fig 1I and S1H Fig). Detection of marker genes that are specific for or excluded from feather primordia (*CTNNB1*, *AXIN2*, *EDAR*, *EDA*, *FGF20*, and *BMP4*) in CAG-GFP embryonic skin revealed that changes in gene expression occurred simultaneously with changes in mesenchymal cell density. Time-lapse imaging of feather pattern formation in the CAG-GFP skin (S1 Video) shows the appearance and resolution of unstable intermediates. In fixed skin specimens, shapes consistent with such unstable intermediate condensations also show overlapping gene expression (S1H Fig) highlighting the tight temporal coincidence of these two phenomena. This indicates that the formation of the feather periodic pattern does not arise from a strict reaction-diffusion prepatterning system or a pure mesenchymal cell motility-led system but has key features of both. These observations contrast with observations made during mouse primary hair follicle formation, in which periodic foci of gene expression are observed prior to mesenchymal cell condensation [46].

**Fig 2:**
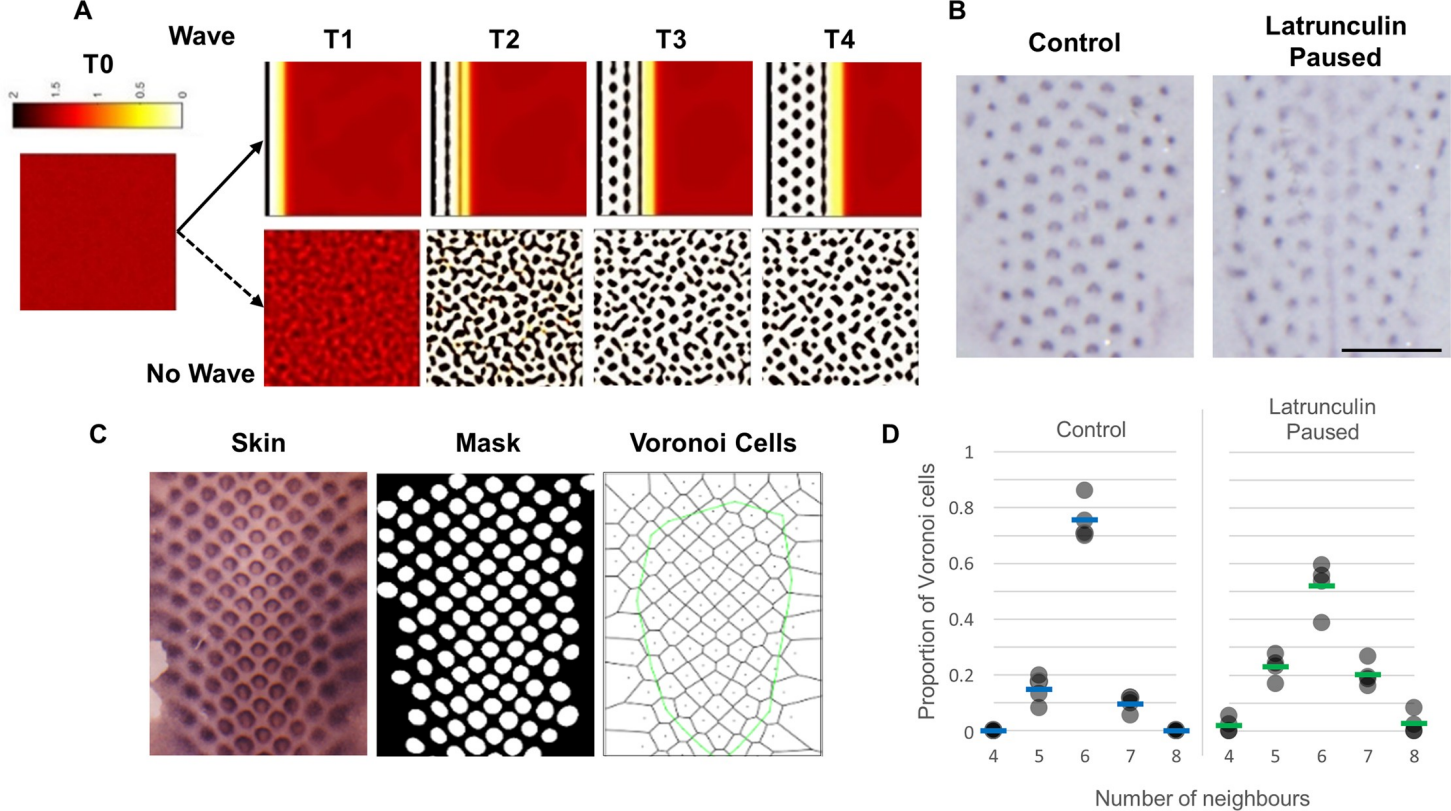
A travelling wave of primordium induction is required for high-fidelity pattern formation. (A) Time course output of a simulation of the patterning network shown in Fig 1J. A 2-dimensional patterning (bottom) yields an irregular array, whereas the same network deployed in a wave (top) yields a highly regular hexagonal pattern. (B) E6.5 skin explants cultured for a total period of 48 hours. Control explants display a hexagonal periodic pattern of high regularity. Explants treated for 24 hours with 150 ng/ml Latrunculin A to suppress feather primordium formation, followed by 24-hour incubation in normal medium to permit patterning, produces a pattern of low regularity. Scale bar: 1 mm. (C) Schematic of pattern fidelity analysis. *CTNNB1* expression was detected and imaged, and a mask of the image was generated. Voronoi tessellation was constructed with primordium centroids as seeds. Boundary Voronoi cells were removed. (D) Plot of the distribution of shapes of Voronoi cells (i.e., number of first neighbours) in control and Latrunculin-paused skin patterns, as in panel B. The proportion of each *n*-sided shape for each skin sample is shown as a single data point. The numerical values for D can be found in S2 Data. E, embryonic day.

From these experimental results, we developed a model of the molecular and cellular interactions involved in feather pattern formation (Fig 1J). In this model, local epithelial FGF stimulates mesenchymal cell aggregation. The localised aggregation of mesenchymal cells activates both the expression of *BMP4* and amplifies epithelial *FGF20* production. BMP proteins diffuse and inhibit FGF production through transcriptional suppression of *FGF20*. The regulation of cell aggregation by BMP’s suppression of FGF production, together with the local depletion of mesenchymal cells from the regions surrounding the aggregates, results in the formation of discrete and characteristically spaced feather primordia. Such a structure of interactions, incorporating aspects of both reaction-diffusion and mesenchymal condensation into a hybrid reaction-diffusion-taxis model, is capable of breaking symmetry to produce a periodic pattern de novo (Fig 2A). We have recently reported a detailed mathematical analysis of this network structure and its properties [47].

### High feather-pattern fidelity is achieved by patterning in a wave

At the ideal packing density, periodic patterns tend to form a hexagonal lattice. In silico modelling using only the interactions described in Fig 1J [47] did not replicate the chicken skin pattern but instead produced a dappled pattern rather than an evenly hexagonal array. Only when this patterning system is driven by a travelling wave of activation are the spatiotemporal progression of sequential row insertion and a uniformly hexagonal patterning outcome observed (Fig 2A).

We tested in embryonic skin the importance of a wave of patterning in producing a hexagonal arrangement by imposing a transient block on patterning. We noted that inhibition of cell movement, and thus primordium formation, in developing explants via Latrunculin A treatment is reversed once the chemical is removed. E6.5 skin explants cultured with a 24-hour Latrunculin A treatment followed by another 24 hours under control conditions displayed irregular primordium arrangements (Fig 2B), consistent with the absence of a patterning wave resulting from the temporary block on cell movement.

To quantify pattern fidelity, defined as the tendency of the lattice of feather primordia to attain a hexagonal arrangement, images of cultured explants were masked and individual primordia were used as seeds to construct a Voronoi diagram (Fig 2C). The distribution of number of neighbours of the Voronoi cells gives a measure of the hexagonality of the experimentally observed patterns. Approximately 75% and 50% of the Voronoi cells in control skin samples and Latrunculin A–paused explants, respectively, exhibited six neighbours, demonstrating a decrease in pattern fidelity in the latter (Fig 2D). These results are consistent with the presence of a pattern-triggering wave spreading across the skin, such that when the Latrunculin block is removed, patterning does not reinitiate at the midline but occurs essentially simultaneously across the entire tract.

### An expanding wave of EDA interacts with a receding wave of β-catenin/EDAR to trigger *FGF20* expression

The nature of the wave that triggers feather patterning has been elusive since the first descriptions of sequential feather row appearance [11,48–50]. Initiation of feather primordia was reported by Shyer and colleagues [16] to rely on local tissue compression, which was mimicked experimentally by permitting the skin to contract by culturing without a supporting substrate. We assessed whether such local compaction might serve to initiate patterning of the dorsal tract by preparing skin cultures in which a strip of skin parallel to and displaced from the midline was free of the underlying support. This strip was compressed, while the remainder of the skin, including the midline, was pinned taut to a filter to prevent generalised compression. In this condition, we observed an unaltered initiation site and timing of patterning, starting as normal at the midline and expanding symmetrically across the control and experimentally compressed sides of skin at the same rate (S3 Fig). This indicates that feather pattern initiation and its spread from the midline does not rely on heterogeneities in tissue compression.

**Fig 3:**
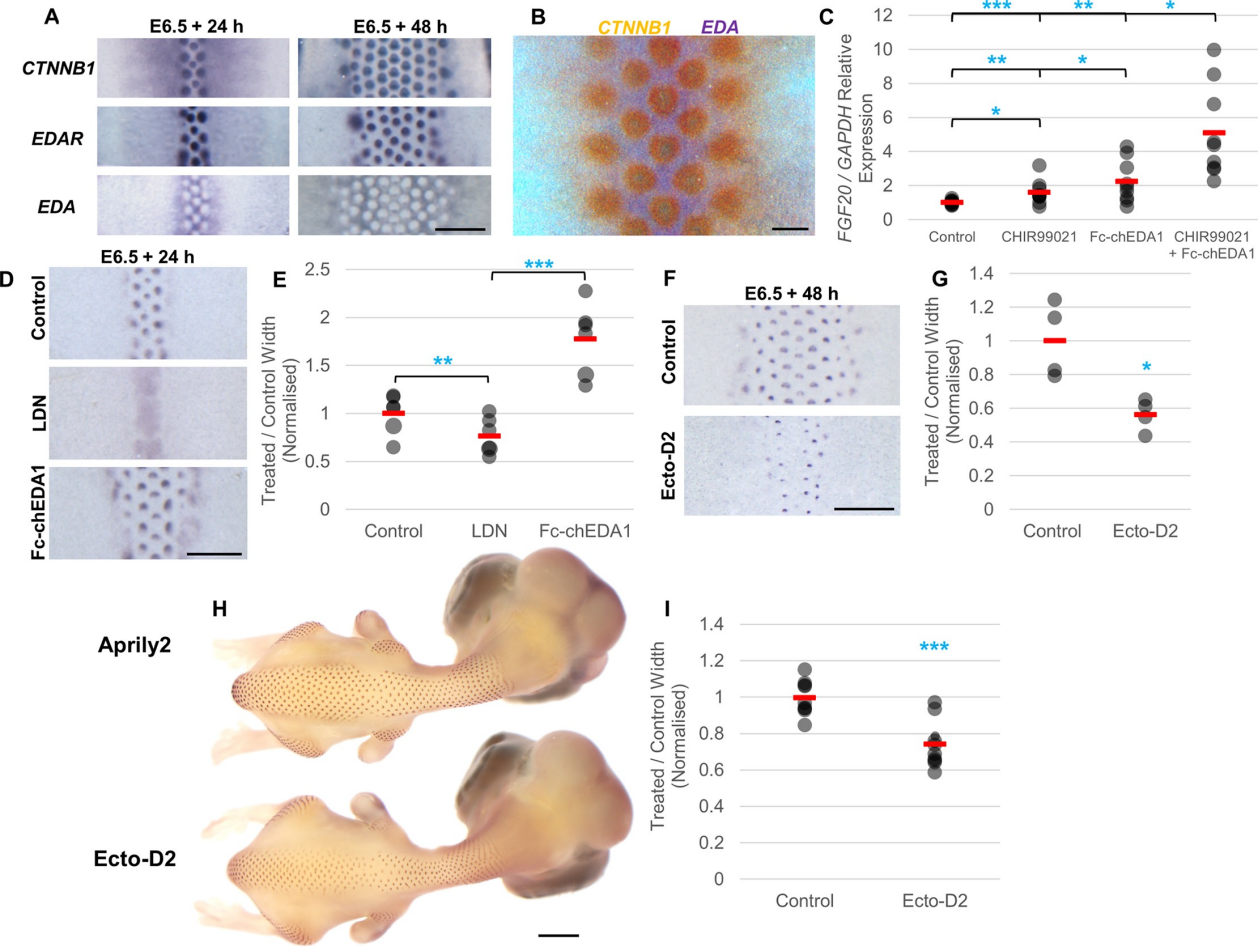
An EDA travelling wave triggers feather patterning. (A) E6.5 skin after 24 hours and 48 hours in culture showing expression of *CTNNB1*, *EDAR*, and *EDA* transcripts. Scale bar: 1 mm. (B) Whole-mount double in situ detection of *CTNNB1* (orange) and *EDA* (purple) at E7.5. Scale bar: 200 μm. (C) qRT-PCR analysis of *FGF20* expression levels in E6.5 skin after 5 hours in 20 μM CHIR99021 (an activator of WNT/β-catenin signalling), 50 ng/ml Fc-chEDA1 (an activator of EDAR signalling), or a combination of both. Filled circles indicate individual data points; red bars indicate the mean. Statistical significance was calculated using a Student *t* test (*p < 0.05, **p < 0.01, ***p < 0.001). (D) Comparison of the periodically patterned region, defined by *FGF20* expression, in E6.5 skin cultured for 24 hours in the presence of either 15 μM LDN193189 (an inhibitor of BMP signalling) or 2 μg/ml Fc-chEDA1. Scale bar: 1 mm. (E) Quantification of patterned region width in control, LDN193189-treated, and Fc-chEDA1-treated explants. Statistical significance was calculated using a Student *t* test (**p < 0.01, ***p < 0.001). (F) Comparison of patterned region width in E6.5 skin cultured for 48 hours in either control or 10 μg/ml Ecto-D2 (an inhibitor of EDA/EDAR signalling) supplemented medium. Scale bar: 1 mm. (G) Quantification of patterned region width in control and Ecto-D2-treated explants. Statistical significance was calculated using a Student *t* test (*p < 0.05). (H) Detection of *FGF20* expression in in ovo control antibody (Aprily2) and Ecto-D2-treated embryos for comparison of feather patterned region width. Scale bar: 2 mm. (I) Quantification of width of primordium carrying dorsal region in Ecto-D2 in ovo injected E8.5 embryos compared to controls. Statistical significance was calculated using a Student *t* test (***p < 0.001). The numerical values for C, E, G, and I can be found in S3 Data. BMP, bone morphogenetic protein; E, embryonic day; EDA, Ectodysplasin A; EDAR, EDA receptor; qRT-PCR, quantitative reverse transcription PCR.

Based on the mechanistic model defined above, the first step of feather primordium formation is a mild chemotaxis to a source of FGF. We thus reasoned that the key activity of the pattern-triggering wave should be capable of stimulating *FGF20* production, which is often detected in a faint streak ahead of and parallel to the most recently formed feather rows (S4A Fig). The expression or activity of the triggering factor should also expand across the skin coincident with the spread of the feather patterning process. Thus, we began by identifying molecules able to induce *FGF20* expression. Canonical WNT/β-catenin activation and EDA/EDAR signalling have been reported to induce *FGF20* expression in mouse skin [51]. We found that stimulation of each of these pathways, with CHIR99021 or recombinant chicken EDA protein (Fc-chEDA1) [52] respectively, rapidly induced *FGF20* expression in embryonic chicken skin (S4B Fig). We also found that stimulation of WNT/β-catenin activity induced *EDAR* expression in cultured skin (S4C Fig). In situ hybridisation revealed that *EDA* is expressed as an expanding wave in dorsal skin (Fig 3A). In contrast, *CTNNB1* and *EDAR* expression are first present in a block of skin defining the presumptive tract, and their expression is then maintained in the feather primordia but lost from the interprimordium space as feather rows are laid down. Thus, in the skin, there is a moving front of loss of *CTNNB1* and *EDAR* expression, starting at the midline and receding towards both sides [32,39], as the *EDA* expression wave advances (Fig 3A). Spreading of *EDA* expression coincident with patterning is also observed in the other anatomical tracts (S4D Fig). Expansion of *EDA* expression and regression of *CTNNB1* expression across the skin is observed in *FGF20*^*sc/sc*^ mutant embryos, which define tracts at the molecular level but fail to undergo subsequent specification of feather primordia (S5 Fig) [29,39]. This demonstrates that neither FGF20 function nor primordium formation is required for the propagation of these waves. Simultaneous detection of *EDA* and *CTNNB1* by double whole-mount in situ hybridisation shows that primordium formation is closely associated with the leading edge of *EDA* expression and the lagging edge of the regressing *CTNNB1* wave (Fig 3B). EDA is a secreted protein [32,53], and its availability may extend beyond the mRNA location detected by in situ hybridisation.

As primordium induction occurs where the EDA wave’s leading edge meets the *CTNNB1* receding edge (Fig 3A and Fig 3B), and because of the ability of both canonical WNT and EDA/EDAR signalling to induce *FGF20* expression (S4B Fig), we reasoned that these two signalling pathways may function synergistically to spark primordium formation at their junction. Indeed, we found that when both signalling pathways were stimulated simultaneously via cotreatment with low doses of CHIR99021 and Fc-chEDA1, *FGF20* expression is induced to a greater degree than in individual treatments, demonstrating a synergistic regulatory effect (Fig 3C). This explains the induction of *FGF20* expression along the stripe where the leading edge of the *EDA* wave meets the receding edge of the *CTNNB1*/*EDAR* wave.

### The EDA wave triggers periodic pattern formation and controls tract expansion

Having shown that a wave of EDA induces *FGF20* expression and is coincident with the onset of periodic patterning, we assessed the functional effects of EDA on feather primordium formation. Specifically, we predicted that if EDA is the limiting factor for triggering pattern formation, then generalised application of EDA should expand the periodically patterned region. Conversely, suppression of endogenous EDA function should restrict pattern expansion. We tested this by treating skin explants with modulators of EDA/EDAR signalling and measuring the width of the periodically patterned region. Ubiquitous stimulation of EDA/EDAR signalling, using recombinant EDA-A1 for 24 hours, almost doubled the width of *FGF20* patterned skin (Fig 3D and Fig 3E), supporting the hypothesis that limited EDA availability normally restricts pattern formation. The converse experiment, that of inhibiting EDA/EDAR signalling with the anti-EDA function–blocking antibody Ecto-D2 [52], narrowed the patterned area compared to control cultures (Fig 3F and Fig 3G), further supporting a key role for endogenous EDA in defining patterning wave expansion. Suppression of BMP signalling, which is required for periodic patterning, did not produce such an effect (Fig 3D and Fig 3E), demonstrating that activation of feather patterning potential—rather than simple suppression of BMP activity—is required to drive the advance of the primordium wave.

To test the role of the EDA wave on feather wave progression in an intact and growing embryo, we introduced the blocking antibody Ecto-D2 into the circulation of E5.5 chicken embryos and continued incubation to day E8.5. Such systemic treatment with Ecto-D2 is capable in mouse of inducing an ectodermal dysplasia phenotype that matches that of *EDA* gene mutation in exposed embryos [52]. In chicken embryos, this blockade of EDA/EDAR signalling permitted feather patterning but restricted the width of the feather pattern in all tracts as compared to controls (Fig 3H and Fig 3I; S6 Fig), agreeing with the results from skin culture experiments. This treatment also abolished the formation of scleral papillae of the eye, similar to but more severe than the effect of the *FGF20*^*sc*^ mutation on these structures (S7 Fig). The feather primordia generated under conditions of EDA suppression failed to develop fully, yielding largely naked skin by incubation days E11.5 and E13.5 (S8A Fig and S8B Fig) and indicating an ongoing need for EDA/EDAR signalling during feather outgrowth. Although placodes and condensates are formed in conditions of EDA suppression, at E8.5 their expression of *FGF20* is not polarised normally (S8C Fig and S8D Fig), and this may lead to a failure of their morphogenesis and disappearance over the following days. The converse experiment, stimulating EDA/EDAR signalling in ovo using the EDAR-activating antibody mAb-EDAR3 [54] at E5.5 to E7.5, expanded the feather pattern in the dorsal tracts, also agreeing with the ex vivo explant culture experiments (S9 Fig).

### A cell density wave precedes the EDA wave to produce the feather patterning field

**Fig 4:**
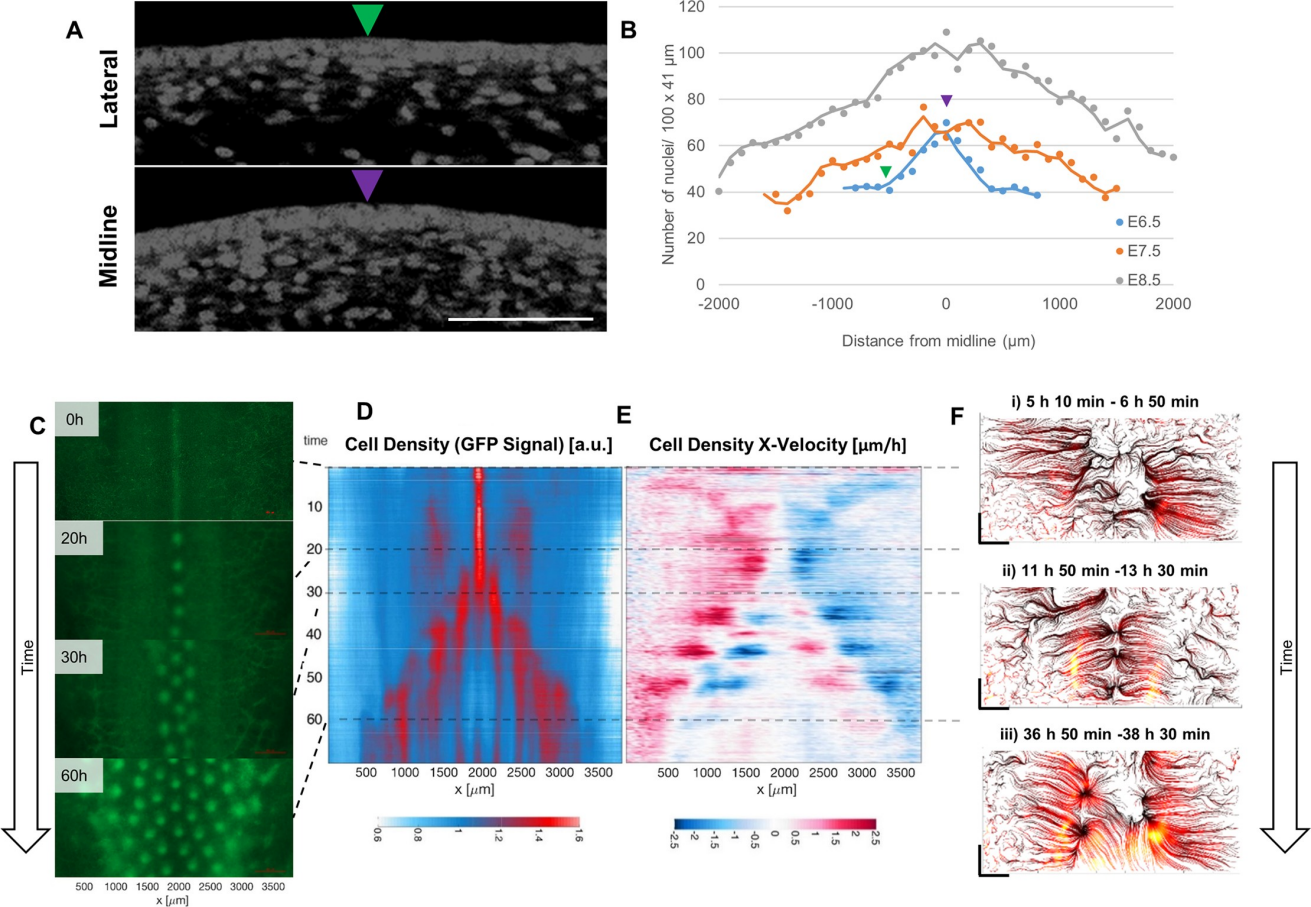
Mesenchymal cell density dynamics in dorsal skin. (A) DAPI-stained sections of E6.5 skin from the lateral and medial regions of the dorsal tract. Medial regions have a higher density of dermal mesenchymal cells than lateral regions. Scale bar: 50 μm. (B) Mesenchymal cell density quantification across the dorsal tract of E6.5, E7.5, and E8.5 chicken embryos. Arrowheads denote areas of tracts displayed in A. (C) A series of stills from a 70-hour time-lapse imaging of feather patterning during dorsal tract development in CAG-GFP skin, starting at E6.5. Intense GFP signal corresponds to regions of high cell density. Anterior is to the top of each panel. (D) Density kymograph shows high cell density medially and in lateral zones at early stages, followed by sequential feather row formation. (E) X-velocity kymograph depicting displacement of cell density (red: rightward; blue: leftward). A wave of generalised cell density displacement towards the midline occurs at early stages, followed by bilateral feather row formation from 30 hours. (F) Streamlines computed for average displacement fields of 10 consecutive frames showing different phases of primordium formation: (i) cell displacement towards the midline, (ii) formation of individual feather primordia of the midline row, and (iii) formation of the primordia in newly induced rows. Scale bar side: 500 μm. The numerical values for B can be found in S4 Data. a.u., arbitrary units; E, embryonic day; GFP, green fluorescent protein.

These findings show that EDA availability controls the spatiotemporal wave of periodic patterning in avian skin but that this signal is neither absolutely necessary nor absolutely sufficient for this process. Thus, a second factor, independent of EDA, must be both limiting for and sufficient to initiate periodic patterning. Based on the long-recognised importance of cell density in feather development [8,10], we considered mesenchymal cell density as a candidate for this second factor limiting competence to undergo periodic patterning. To characterise mesenchymal cell density at different stages of skin development, we counted cells on sectioned embryos, finding a medial-lateral decreasing gradient of cell density (Fig 4A and Fig 4B). Quantification of cell density across the presumptive tracts of developing *FGF20*^*sc/sc*^ embryos lacking mesenchymal condensates, and so used to avoid the distortion of mesenchymal distribution by cell recruitment to forming condensates, shows that this medial-high cell density gradient is maintained throughout the period of patterning, even as total mesenchymal cell density increases (Fig 4B). Thus, the EDA wave travels across a dynamic gradient of mesenchymal cell density as it triggers feather formation.

To trace the cell density dynamics involved in the emergence of periodic pattern on the dorsal tract, we performed time-lapse imaging of cultured embryonic skin from the CAG-GFP transgenic line. Time-lapse imaging of skin begun with tissue collected at E6.5 shows a stripe of high cell density along the dorsal midline. Over the following 70 hours, cells rearrange to produce an ordered periodic pattern with the breaking up of this stripe into a row of spots, followed by bilateral additions of pairs of rows of feather primordia (Fig 4C). During this process, extensive changes in cell density and dynamic relocation of cell aggregates are apparent (S1 Video).

To represent cell density across the skin over time in a 2-dimensional form, we plotted GFP signal intensity as a kymograph (Fig 4D). Each frame from the time-lapse series is depicted as a single line, with red indicating high and blue indicating low cell density. This depiction compresses the 2-dimensional information of each original frame into a line that depicts cell density relative to the left-right axis of the original image, with the compression removing anterior-posterior information. The y-axis on the kymograph is produced by stacking these frames to show the progression of cell density over time. This plot shows the initial midline stripe of high cell density, soon followed by the emergence of two broad lateral regions of high cell density between 10 and 25 hours, after which the addition of the first lateral feather primordium rows occurs from 25 hours. Progressive addition of rows is apparent as the emergence of additional vertical red stripes, though ongoing cell depletion from interprimordium intervals leads to a reduction in overall fluorescence for the most mature (i.e., central) rows.

To track general cell motility, we plotted a velocity kymograph (Fig 4E) showing for each pair of frames compressed along their y-axes the change in cell density (i.e., the change in GFP signal intensity) occurring across the time course of imaging, with blue indicating leftward movement and red indicating rightward movement. Thus, meeting points of red and blue, where red is to the left of blue, show the sites of cell accumulation. This plot shows a generalised movement of cells towards the midline at early stages of patterning, even before the first periodic condensates become apparent there. This widespread midline movement is followed by progressively more lateral local clustering at later stages to form the distinct feather rows. We used streamlines calculated from a particle image velocimetry (PIV)-based velocity field averaged over 10 consecutive frames to better represent cell behaviours during specific phases of dorsal tract patterning (Fig 4F), here maintaining the x-axis (left-right) and y-axis (anterior-posterior) of the original skin images. This shows the initial generalised flow of cells towards the midline (time 5:10–6:50), followed by periodic patterning of condensates on the midline (11:50–13:30) and then cell condensation laterally to form the next rows of feather condensates (36:50–38:30).

To assess the extent to which these observations reflect cellular events specifically in the mesenchymal component of the skin, we visualised mesenchymal cells by transplanting somites from the newly developed tdTomato (TPZ) transgenic line (S1 Supporting Methods) into a CAG-GFP host embryo. Four days after somite transplantation, we collected the chimeric skin at day E6.5 for time-lapse imaging (S10A Fig and S10B Fig). In this condition, the somite derivatives, which are entirely mesenchymal in the skin [55], will on one side of the embryo produce red fluorescent signal, whereas the host cells, including the overlying ectoderm, will be green. Computational analyses of time-lapse imaging of skin explants prepared from host-GFP/mesenchyme graft–tdTomato chimeras show the same cell behaviours as observed in entirely CAG-GFP skin, demonstrating that the key behaviours observed arise from mesenchymal cell dynamics (S10C Fig and S2 Video). Together, these investigations show that the process of dorsal tract patterning involves an initial movement of mesenchymal cells towards the midline, followed by laterally expanding dense dermis and rapid cell density changes to generate a periodic condensate array.

### EDA sets the mesenchymal density threshold required for periodic patterning

**Fig 5:**
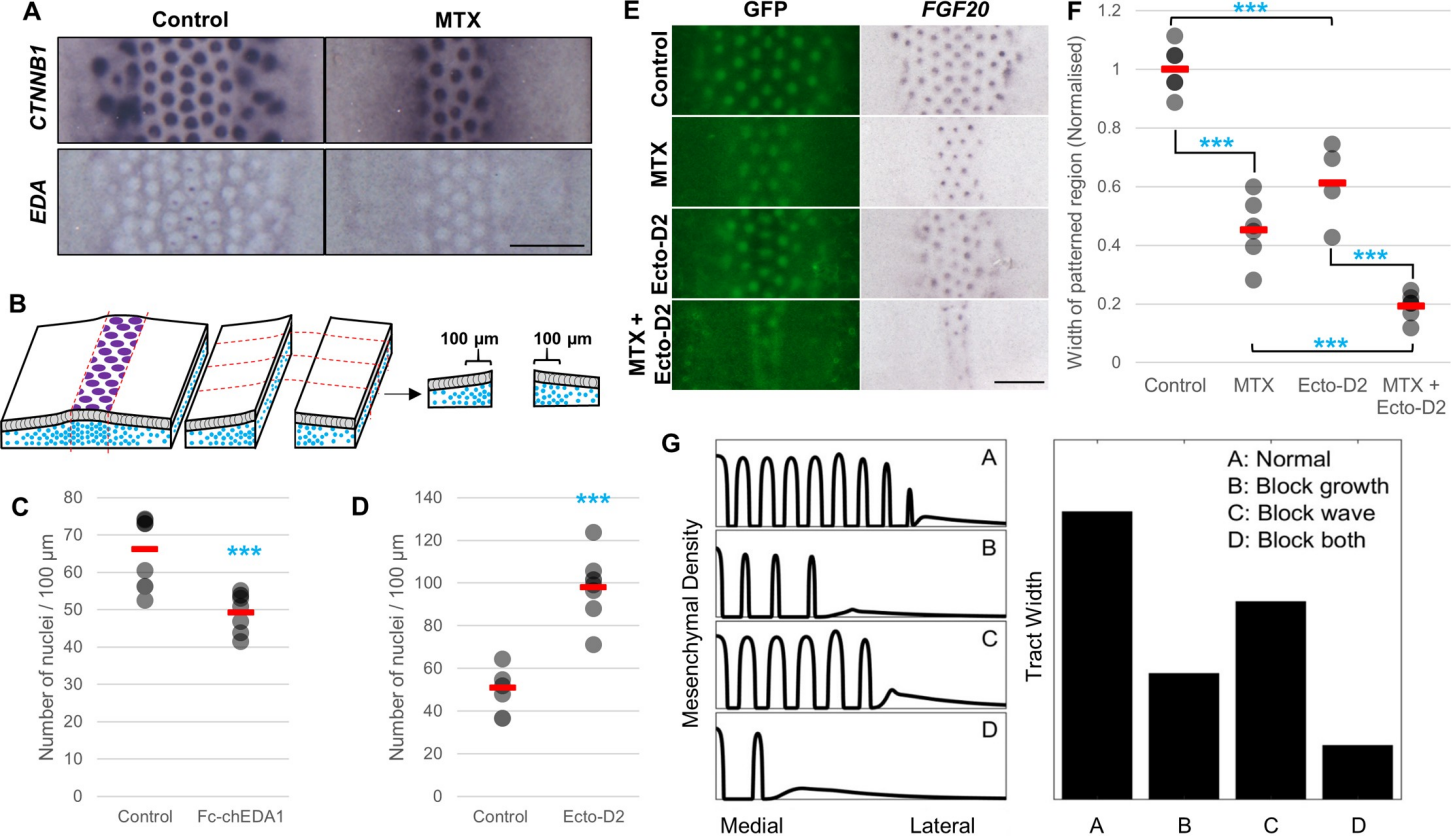
Mesenchymal cell density and EDA signalling interact during skin patterning. (A) E6.5 skin cultured for 48 hours in 5 μM MTX, an inhibitor of cell proliferation. Primordium formation is detected by *CTNNB1* expression. Inhibition of cell proliferation reduces the width of the patterned region, but the extent of the *EDA* expression wave is unaffected. Scale bar: 2 mm. (B) Schematic of assessment of the critical cell density at which patterning occurs. (C) and (D) Mesenchymal cell density within 100 μm of the edge of the patterned region of skin explants in the presence of (C) an activator (Fc-chEDA1) or (D) an inhibitor (Ecto-D2) of EDA/EDAR signalling. Filled circles indicate individual data points; red bars indicate the mean. Statistical significance was calculated using a Student *t* test (***p < 0.001). (E) E6.5 CAG-GFP skin explants cultured for 48 hours with 5 μM MTX, 10 μg/ml Ecto-D2, or both. Scale bar: 2 mm. (F) Quantification of patterned region widths, based on *FGF20* expression, shows reduction in patterned region of cotreated samples compared to each single treatment. Statistical significance was calculated using a Student *t* test (***p < 0.001). (G) One-dimensional representation of a simulation of the network shown in Fig 1J, with peaks representing high cell density (condensates). Reduction of the wave or of cell growth restricts the spread of pattern, whereas reduction of both has a stronger effect. The numerical values for C, D, F, and G can be found in S5 Data. E, embryonic day; EDA, Ectodysplasin A; EDAR, EDA receptor; GFP, green fluorescent protein; MTX, methotrexate.

To assess the functional impact of mesenchymal cell density on feather patterning, we reduced cell density by suppressing proliferation using methotrexate (MTX). This treatment in culture reduced cell density in the dermis (S11 Fig) and limited the area of the feather patterned region, causing it to arrest at five normally spaced rows. This reduction in feather row number occurred without any change in the gene expression waves of *CTNNB1* and of *EDA*, which continued to extend to the normal edge of the presumptive tract (Fig 5A). Thus, the molecular wave propagates independently of either the cell density wave or of feather primordium formation.

We assessed the mesenchymal cell density at which patterning occurs in different EDA signalling regimens by making incisions directly adjacent to the *FGF20-*defined margin of the patterned region of EDA modulated explants and quantifying the mesenchymal cell density within the first 100 μm lateral to the pattern margin (Fig 5B). Application of soluble EDA, which led to expansion of the patterned region (Fig 3D and Fig 3E), permitted patterning at a lower mesenchymal cell density than normal (at the lateral pattern margins 66 cells/100 μm versus 49 cells/100 μm after 24 hours in culture) (Fig 5C). Conversely, suppression of EDA function permitted patterning after 48 hours in culture only where a density of 98 cells/100 μm was attained (Fig 5D), restricting patterning to a narrow strip at the midline (Fig 3F and Fig 3G). Thus, EDA signalling lowers the mesenchymal cell density at which the dermis becomes permissive to periodic pattern formation.

To test whether EDA signalling sets the critical mesenchymal cell density quorum independently of skin location, we assessed the effects of combined modulation of both cell density and EDA function. As before, suppression of either cell proliferation or of EDA function individually reduced the width of the patterned region of the skin. Combined suppression of cell density and EDA function yielded a still greater reduction of the periodically patterned region (Fig 5E and Fig 5F), showing that a high cell density, rather than any other means of circumventing EDA activity, is required for medial skin patterning in conditions of EDA suppression. Simulations of the patterning process involving a priming wave, representing EDA, and including a mesenchymal cell density gradient show that such behaviour is expected of the network upon suppression of cell density, suppression of EDA, or the enhanced effect of suppressing both (Fig 5G) (see S2 Supporting Methods). Thus, mesenchymal cell density has a defining effect on determining whether periodic patterning occurs or not, with EDA setting the minimum density of mesenchymal cells required to undergo pattern formation.

### Continual movement of individual mesenchymal cells occurs in loose and dense dermis prior to and during condensate formation

**Fig 6:**
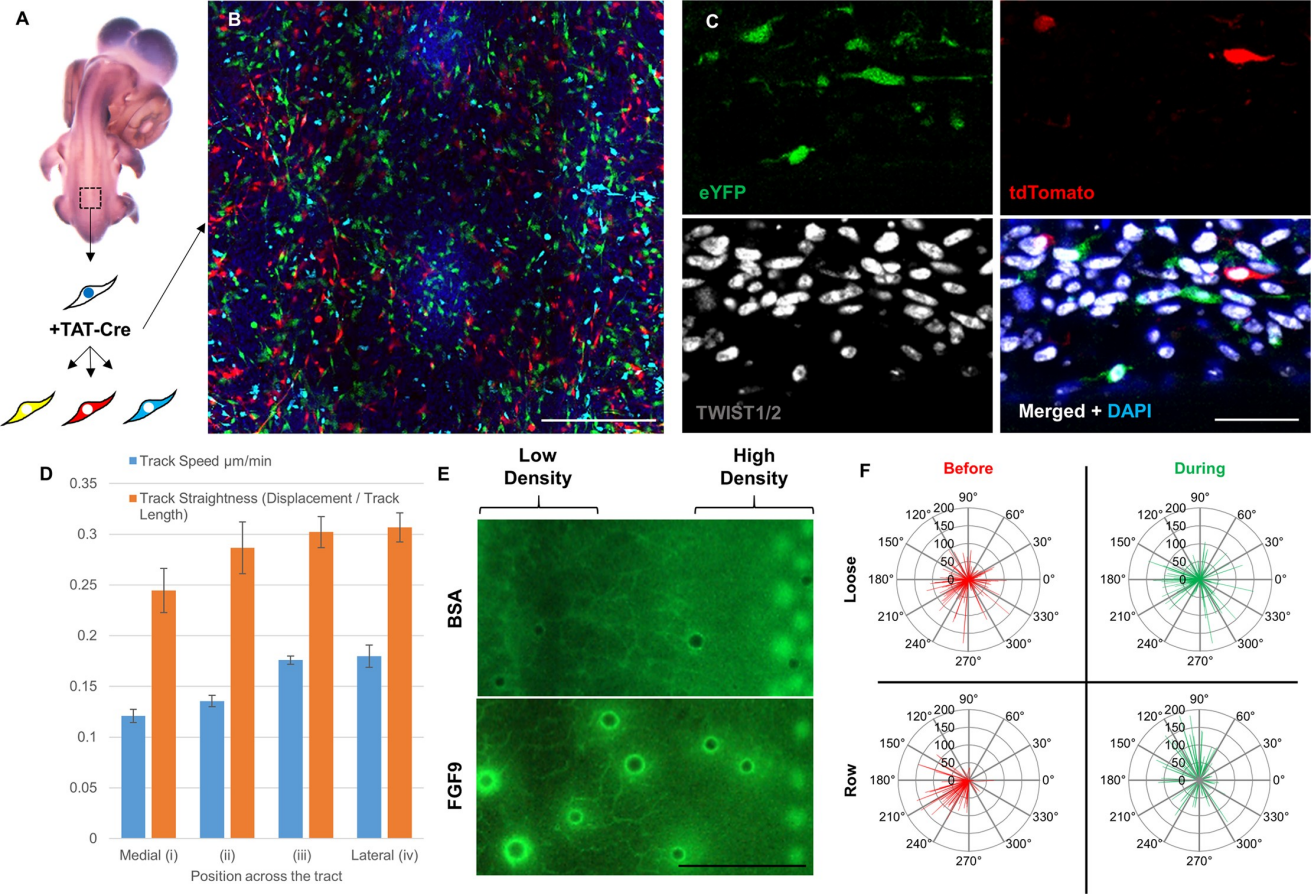
Individual mesenchymal cell movement and morphology are unaffected by cell density. (A) Cells of the Chameleon line express Nuclear Blue until exposure to Cre recombinase, which causes cells to express one of three colours of cytoplasmic fluorescent proteins: red, cyan, or yellow. (B) E6 Chameleon skin treated with TAT-Cre and cultured for 24 hours, demonstrating sparse labelling of cells. Two feather condensates are present at the midline (vertical centre). Scale bar: 200 μm. (C) Frozen transverse section of TAT-Cre labelled Chameleon mesenchyme. Colocalisation of nuclear TWIST1/2 staining (a marker of mesenchymal cells, white) and cytoplasmic tdTomato or eYFP (shown as green) confirms that labelled cells have dermal mesenchymal identity. Scale bar: 20 μm. (D) Mesenchymal cell speed and track straightness plotted for each region of skin, from medial (i) and intermediate (ii, iii) to lateral (iv). Analysis derived from confocal time-lapse imaging of labelled E6.5 Chameleon skin explants cultured for 24 hours. Error bars indicate S.E.M. (E) CAG-GFP explants cultured for 24 hours carrying either BSA- or FGF9-coated beads across the presumptive dorsal tract (high density) and intertract loose mesenchyme (low density). Scale bar: 1 mm. (F) Angle plots showing direction of individual mesenchymal cell movement in the 10 hours before and the 10 hours during the formation of a feather primordium row (Row) or the same time period in loose mesenchyme that does not form feather primordia (Loose). Line length indicates cell movement track length. Midline: 180°, Anterior: 90°. The numerical values for D and F can be found in S6 Data. BSA, bovine serum albumin; E, embryonic day; eYFP, enhanced yellow fluorescent protein; FGF, fibroblast growth factor.

Having defined a requirement for a mesenchymal quorum in feather patterning and its relationship to EDA, we assessed cell behaviour in dense and loose dermis. We aimed to determine the mode of cell accumulation at condensates and whether differences in cell behaviour might account for the requirement for a minimal mesenchymal density to initiate periodic patterning. To track single cells and quantify their behaviour, we employed embryos of the Chameleon transgenic chicken line. This line carries an array of fluorescent protein encoding genes, one of which is selected for expression upon Cre-mediated recombination at the transgene locus ([56] and Saunders and colleagues in preparation). We treated cultured Chameleon transgenic skin with a cell-permeable TAT-Cre to sparsely label cells, applying the reagent to the mesenchymal side of the dissected skin. This results in stochastic labelling of scattered cells with different colours (Fig 6A and Fig 6B). Detection of TWIST1/2 (also referred to as Dermo-1), a nuclear marker of mesenchymal cells contributing to pattern formation and dermal condensates [57–59], shows Cre-mediated recombination and resulting cytoplasmic fluorescence in this cell population at all levels in the dermis (Fig 6C).

We collected time-lapse confocal images of cells in such sparsely labelled skin during patterning in culture, permitting the tracking of individual cells. We observed that cells in all parts of the skin move, with cells in the epidermis moving in a highly coherent manner, as predicted based on their physical attachment to one another in a sheet (S3 Video, S12A Fig). We analysed cell behaviour by confocal layer, finding that the more superficial layers, which are epithelial, undergo coordinated cell movement, whereas the deeper layers of mesenchyme have cells undergoing random and uncoordinated movement (S12A Fig). However, we detected very little movement upward or downward at any skin depth, with most cells remaining in the same confocal layer throughout and thus moving in parallel with the plane of the epidermis (S12B Fig). The net movement for individual mesenchymal cells over the course of imaging occurs almost entirely towards the midline (S12C Fig).

By measuring individual mesenchymal cell behaviours over the entire time course of imaging, we found that cells in dense and loose dermis move, though somewhat more rapidly in the lateral, loose dermis (Fig 6D and S4 Video). This may result from freer movement of cells where there are fewer cell–cell interactions. Thus, the failure of patterning in loose dermis is not a result of impaired cell movement, nor is it due to an inability to respond to FGF sources, as FGF beads are readily capable of inducing cell aggregation in both dense and loose dermis (Fig 6E). By tracking cells specifically prior to and during the formation of condensates, we observe that before the formation of a new primordium row, cells move medially; but to form the condensates themselves, movement is predominantly along the anterior-posterior axis (Fig 6F). Throughout this process, there is remarkably constant migration speed and straightness (persistence), though the midline-directed cell movement occurring before condensate formation displays the greatest persistence of all classes (S13A and S13B Fig). Individual condensates are thus formed by the FGF-guided accretion of cells that move constantly with approximately the same velocity and persistence.

The skin’s mesenchymal cells display extensive and dynamic projections when they are both outside and within condensates, and this morphology does not differ between low- and high- cell-density mesenchyme (S13C Fig). We used the MacGreen reporter line, which expresses GFP specifically in macrophages under control of *CSF1R* regulatory elements [60], to detect macrophages in embryonic chicken skin. These cells are distinct from other mesenchymal cells in size of cell body and characteristics of the projections, confirming that we are not inadvertently tracking this cell population (S13D Fig). Together, this approach shows that in morphology and movement, mesenchymal cells in dense and loose regions display very similar observable behaviours. Thus, the high-cell-density dependence for feather development more likely rests on a need for rapid mesenchymal cell condensation and stabilisation of *FGF20* expression than on distinct individual cell phenotypes in dense and loose mesenchyme.

### Loss of feather patterning waves in flightless birds

**Fig 7:**
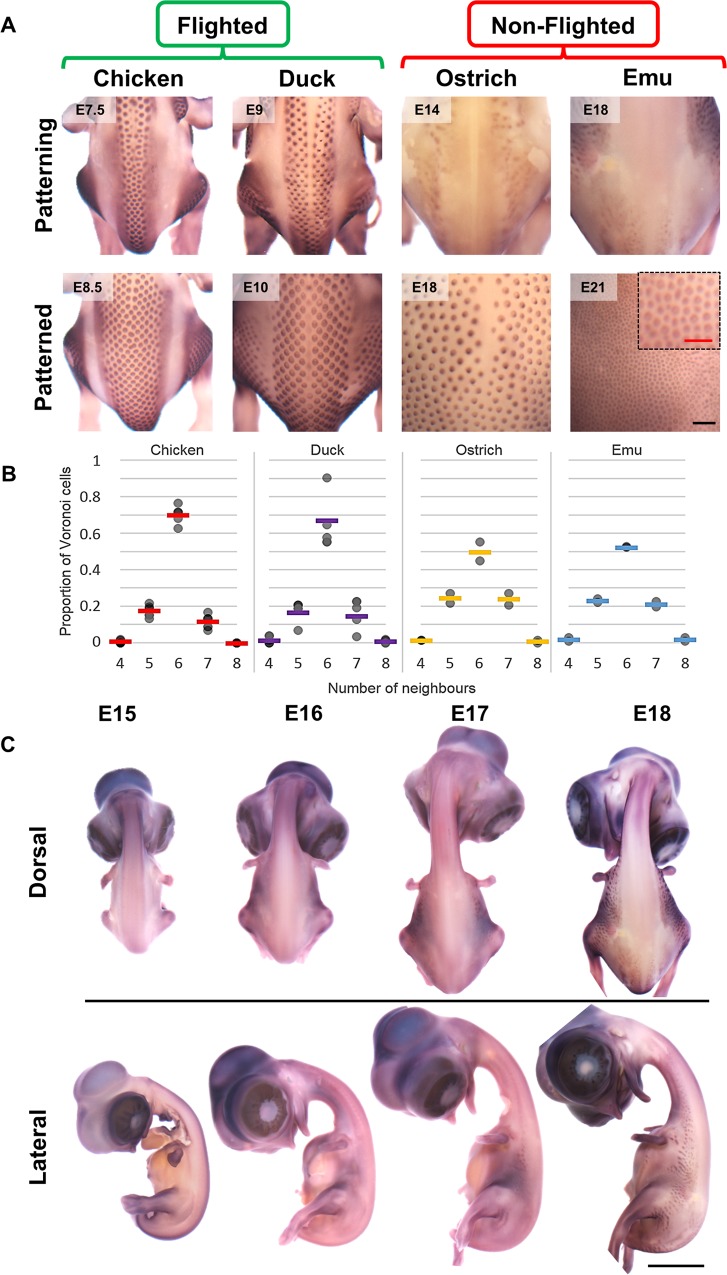
Low-fidelity feather pattern in flightless birds. (A) Visualisation of feather primordia (*CTNNB1* gene expression) during and after dorsal patterning in flighted (chicken and duck) and nonflighted (ostrich and emu) birds. Both flighted species display a high-fidelity hexagonal arrangement of feather primordia, whereas nonflighted species display a low-fidelity arrangement. Inset: high-magnification image of the corresponding panel showing low-fidelity arrangement of the small emu primordia. Scale bar: 1 mm (Inset: 500 μm). (B) Measurement of the fidelity of primordium arrangements of chicken, duck, ostrich, and emu dorsal regions. Each data point represents the proportion of the specified *n*-sided shape from a single piece of skin. (C) In situ hybridisation detecting *CTNNB1* expression in emu embryos prior to and during feather patterning. Dorsal and lateral views of embryos from the ages indicated are shown. Scale bar: 5 mm. The numerical values for B can be found in S7 Data. E, embryonic day.

We find that the skin of chicken, duck, ostrich, and emu embryos have pronounced differences in their feather arrangements. The two flighted species, chicken (of which wild ancestors and light domestic breeds can fly, though this ability is lost in some breeds because of increased body weight [61]) and duck, display a highly regular feather arrangement, whereas the two flightless species have a much less regular pattern (Fig 7A). Voronoi tessellation analysis shows that flightless species have a pattern of lower hexagonality than the flighted species (Fig 7B). Whereas the flighted species form their feather pattern through a medial-lateral travelling wave, in the flightless ostrich and emu sequential feather primordium formation is absent (S14 Fig).

Prior to patterning, both flighted and flightless (S14 Fig and S15 Fig) species define the presumptive dorsal tracts, denoted by *CTNNB1* and *EDA* expression, but the flightless species lack the spreading wave of *EDA* expression during the period of feather patterning. Rather than patterning in a wave, the ostrich embryonic skin undergoes sporadic patterning within the *CTNNB1*-defined dorsal tract in regions where mesenchymal cells attain a high density (S14A Fig). This ultimately produces a set of tracts that can be mapped onto those present in the chicken anatomy, though with some fusions (S14A Fig and S16 Fig). Indeed, the flighted Chilean tinamou (*Nothoprocta perdicaria*), a ratite related to ostrich and more closely to emu [62,63], has tracts in the same arrangement as chicken, indicating that this arrangement (S17 Fig) is ancestral to all extant birds. In the emu, in contrast, the first signs of feather formation occur late in development, at E17, after loss of *CTNNB1* expression from the presumptive tract. These feather primordia appear outside the characteristic tract locations, beginning instead on the flanks (Fig 7C), which are prominent intertract regions in chicken and ostrich. Sequential double in situ hybridisation to detect *CTNNB1* and *EDA* in emu embryos shows the feather primordia forming specifically in *EDA*-negative skin regions, lateral to the presumptive dorsal tract (S18A Fig).

**Fig 8:**
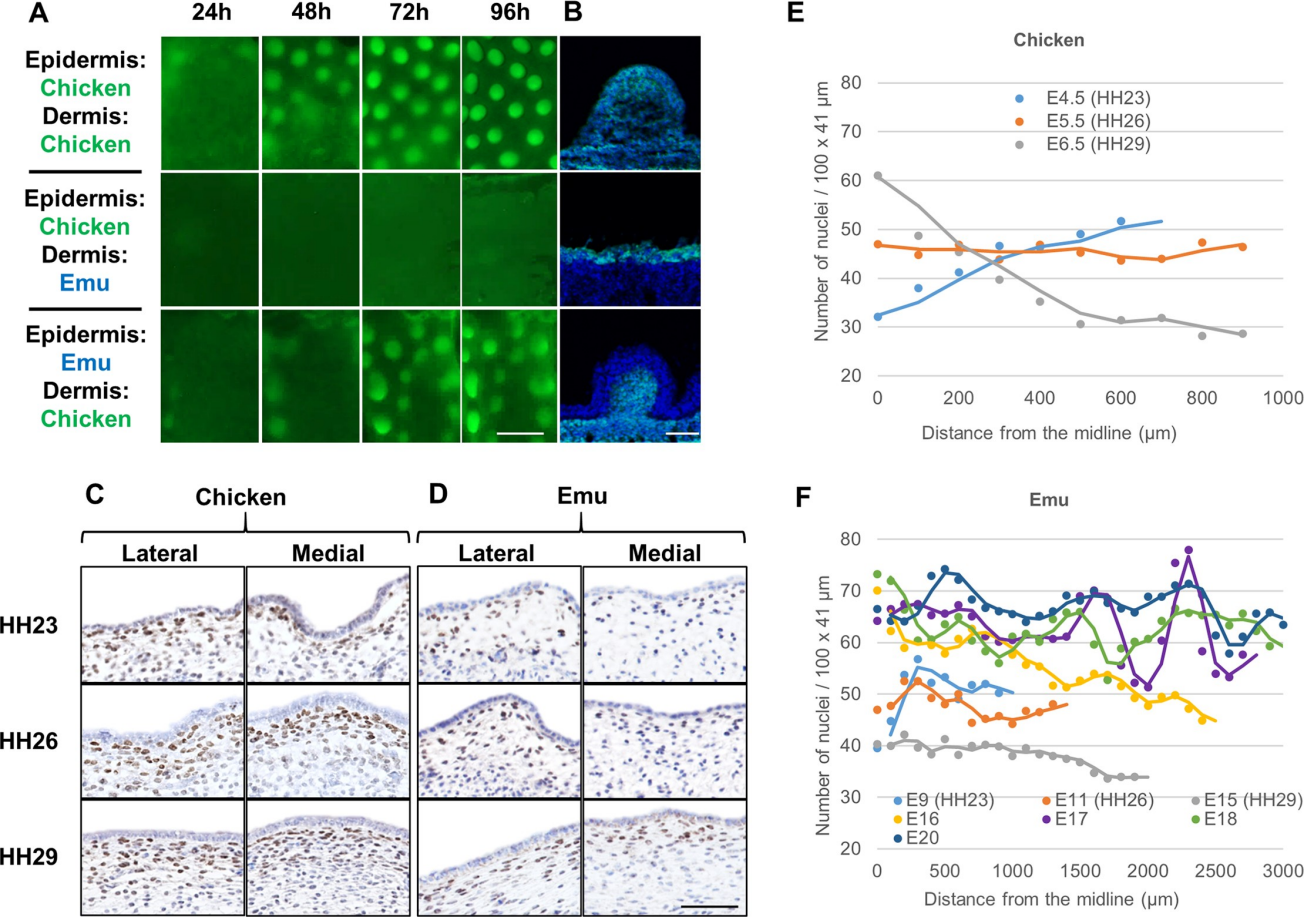
Emu dermis has low density and is not permissive to tract patterning. (A) Epidermal-dermal recombinations between CAG-GFP chicken and emu skin. E6.5 chicken and E14 emu dorsal skins were recombined and cultured for a period of 96 hours. Chicken epidermis and emu dermis do not form feather primordia, whereas emu epidermis recombined with chicken dermis does. Scale bar: 500 μm. (B) GFP detection of chicken cells in sections of recombined skin, stained with DAPI. Scale bar: 50 μm. (C) and (D) Immunodetection of TWIST1/2 protein (a nuclear marker of mesenchymal cells, brown) in the lateral and medial regions of the dorsal skin of embryonic stage HH23, HH26, and HH29 (C) chicken and (D) emu embryos. Scale bars: 50 μm. (E) Quantification of mesenchymal cell densities across the dorsal region of E4.5 (HH23), E5.5 (HH26), and E6.5 (HH29) chicken embryos. (F) Mesenchymal cell density quantification across the dorsal region of emu embryos from E9 to E20. Density increases markedly after E15. The numerical values for E and F can be found in S8 Data. E, embryonic day; GFP, green fluorescent protein; HH, Hamburger Hamilton stage.

To assess whether the absence of feather formation in the emu presumptive dorsal tract is of mesenchymal or epithelial origin, we performed reciprocal epidermal-dermal recombinations between chicken and emu embryonic skin. A combination of chicken epidermis with emu dermis did not produce feather primordia. In the reciprocal recombination, however, emu epidermis can undergo rapid primordium formation in conjunction with chicken dermis (Fig 8A). Use of CAG-GFP transgenic chicken skin allowed tracking of the chicken and emu components of the skin composites in sectioned tissue, confirming the emu origin of epidermis undergoing feather formation when combined with chicken tract dermis (Fig 8B). Therefore, the evolutionary loss of emu feather tract patterning in the dorsal skin is a result of a mesenchymal defect, with the emu epidermis being competent to participate in feather development.

To determine the nature of the mesenchymal defect that abolishes tract patterning in emu, we compared the status of the developing dorsal mesenchyme in chicken and emu embryos. TWIST1/2 staining in developing chicken and emu dorsal skin revealed a profound delay in the densification of the dermis in the presumptive emu tract compared to chicken (Fig 8C, Fig 8D and S18B Fig). Quantification of mesenchymal cell density at these stages shows that the population of the dorsal skin with mesenchyme is limited in emu embryos, which develop a dermis displaying a uniformly low mesenchymal cell density when compared to chicken embryos (Fig 8E and Fig 8F). In earlier-stage embryos, cell proliferation was observed in the dermomyotomal lip of both Hamburger Hamilton stage (HH)21 chicken and emu embryos, as determined by immunodetection of phospho-Histone H3 (Ser10) (S18C Fig), suggesting that delayed dermis densification in emu is not due to the lack of production of mesenchymal cell progenitors but rather due to a failure of cell ingression towards the dorsal midline during emu development. These observations suggest that in emu, the failure to develop a sufficient mesenchymal density at the appropriate time in development underlies the failure of tract patterning by desynchronising the cellular and molecular influences required to act coordinately in the generation of a patterned feather array.

## Discussion

A number of theoretical mechanisms to explain the spontaneous emergence of periodicity in biological systems have been proposed [21,64]. The operation of Turing or reaction-diffusion systems based only on intercellular signalling has been supported with several lines of experimental evidence and argument in diverse organs [65,66]. In mammalian skin, Glover and colleagues provided evidence that a pure reaction-diffusion system, with no reliance on cell movement, normally patterns the primary hair follicles. However, under conditions of altered FGF and BMP signalling, this mechanism can be replaced by a pure mesenchymal cell self-organisation system [46]. Our present work uncovers a distinct process normally operating in avian skin, in which elements of both reaction-diffusion and cell movement–based patterning systems are integrated into a unified periodicity-generating mechanism.

**Fig 9:**
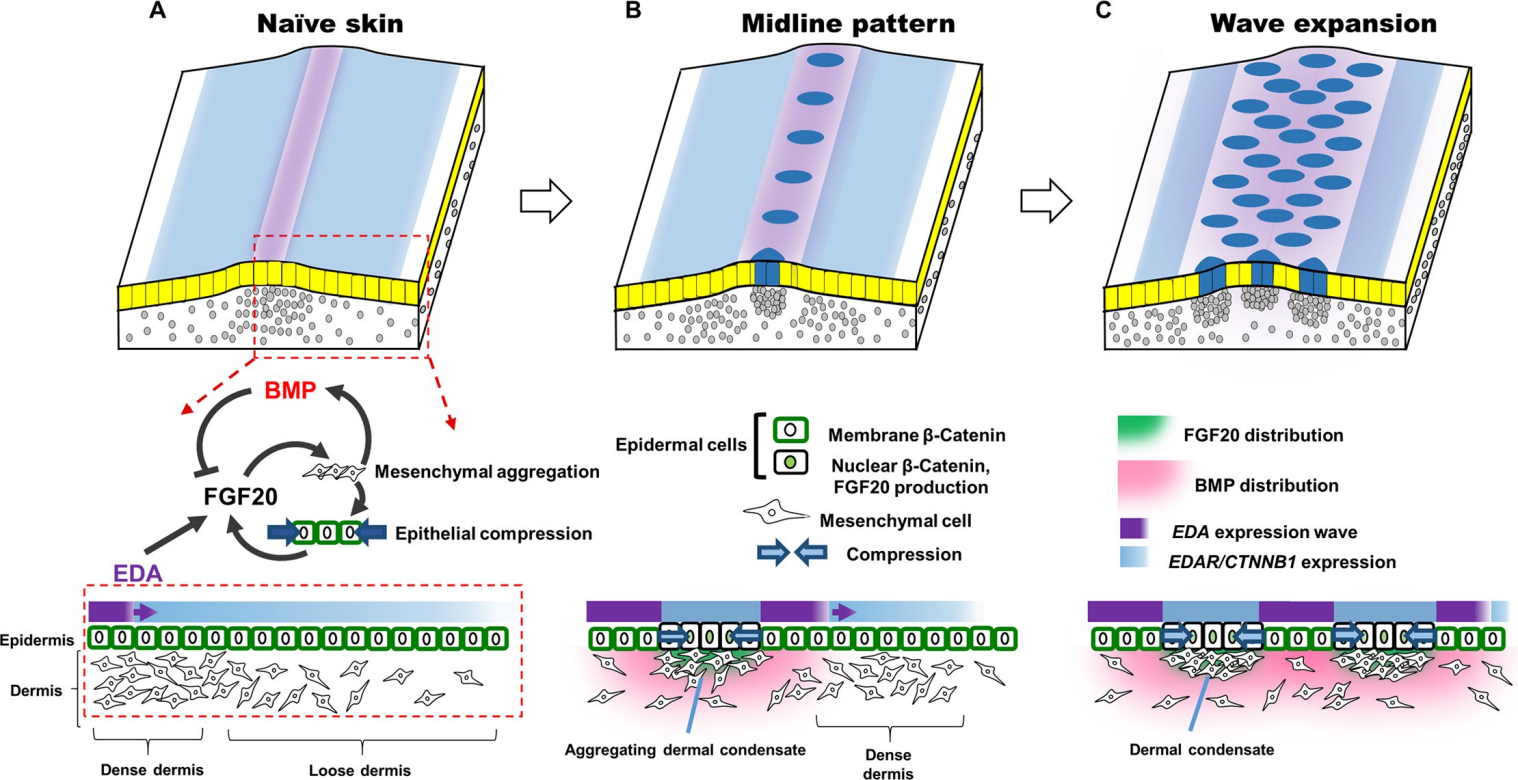
Schematic of the feather patterning mechanism in flighted birds. (A) A competent epidermis overlies a mesenchyme displaying high mesenchymal cell density on the midline (dense dermis) and gradually decreasing to the sides (loose dermis). (B) A travelling wave of EDA signalling stimulates the epidermal production of FGF20, which induces chemotaxis of mesenchymal cells and thus formation of a dermal condensate. As the condensate forms, *FGF20* expression is further stimulated, thereby reinforcing production of this attractant. The clustering of cells also stimulates the production and secretion of BMP to inhibit *FGF20* production, limiting the size of the incipient primordium and preventing fusions between neighbouring primordia. Meanwhile, the previously loose dermis increases in density to reach the threshold density that permits condensate formation. (C) As the EDA signalling wave sweeps across the skin, the reaction-diffusion-taxis system is repeated until the wave terminates at the edge of the tracts. BMP, bone morphogenetic protein; EDA, Ectodysplasin A; FGF20, fibroblast growth factor 20.

This feather periodic patterning system (Fig 9) relies on epithelial FGF to attract mesenchymal cells to form an aggregate, explaining why the absence of *FGF20* in the scaleless mutant leads to a failure of feather primordium formation [29] and demonstrating a defining role for the epidermis in avian skin patterning. The resulting focal clustering of mesenchymal cells reinforces and intensifies epithelial *FGF20* expression, likely through a mechanical triggering of epidermal β-catenin activity via local epidermal compression [16]. This form of positive feedback on FGF20 represents an integration point between Turing/reaction-diffusion and mechanical systems in biological pattern formation, a coupling shown in broad terms to yield robust periodic patterning [67]. Aggregation of cells is coupled with production of BMP by the mesenchymal condensate, inhibiting *FGF20* production and limiting condensate expansion. Mesenchymal cells move continually, and our observations of this process do not support a suggestion of preferred migration along a sparse lattice of collagen fibres to guide mesenchymal condensation [12,68].

This set of interactions is sufficient to break symmetry and produce a periodic pattern only if mesenchymal cells attain a critical density. The requirement for initially weak and ephemeral sources of FGF20 to be sustained and amplified by successful recruitment of mesenchymal cells can explain the cell-density dependence of feather development, without a need for any distinct individual cellular phenotype in dense compared to loose mesenchyme. Such a density effect emerging from a population of cells enacting the same individual behaviours should be seen as distinct from quorum sensing, in which cells sense their collective density and alter their state accordingly [69]. Rather, although we cannot rule out the possible existence of cell phenotypic differences between individual cells in dense and loose mesenchyme, we do not observe such differences, and they may not need to be invoked to understand the transition from loose dermis to a stable array of cell condensates. Events that could be characterised as community effects or quorum sensing may occur later in feather development, however, as development of each condensate progresses and the cells within alter their fate. The formation of condensates in our model is fundamentally determined through biased cell movement in a mesenchymal cell population with a density above a critical threshold. This criticality is a trait shared with another recent model, driven by cell adhesion, that was proposed for chondrogenesis during avian limb patterning [70].

The extracellular signal EDA serves to trigger periodic patterning by lowering the threshold of mesenchymal density required for condensate formation. This is achieved through induction of a transient stripe of FGF20 production where the advancing EDA wave meets the receding β-catenin/EDAR wave. This initially low FGF20 induction serves to begin mesenchymal cell aggregation along the stripe, initiating the cycle of mesenchymal aggregation, which leads to increased epidermal *FGF20* expression and in turn to enhanced mesenchymal attraction. As this process begins, it immediately becomes influenced by the periodic patterning rules defined above and by the effects of the preceding feather primordium row’s inhibition through BMP production and local cell depletion. Together, these influences define the location of condensates in each new row based on the inhibitory template of the previous one, ensuring that the condensates in each new row are placed out of register with the previous row. Repeated iterations of this process produce a hexagonally patterned array, though this may be altered by later growth. Certain aspects of tooth development show parallels to this process of feather formation. In the mouse embryo, the teeth form in sequence along the jaw [71], similar to the sequential formation of feathers along the midline row. A relationship between the primary row of teeth and successive replacement rows is observed in the fish dentition [72]. However, the ongoing formation of these replacement teeth in fish appears to be regulated locally rather than through general activation of an entire patterning field by morphogens [73], as we report in the avian skin. The position of successive teeth is thus associated with the position of the preceding tooth and its final placement not entirely dictated by inhibitory influences.

The spatiotemporal role of EDA in defining the zone in which periodic patterning may initiate is distinct from its described role in other vertebrates. Spontaneous mutations in genes encoding EDA pathway components are known in a range of species of mammals, fish [30], and in a lizard [31]. In each of these species, EDA activity has been shown to be required for hair or scale development but not for patterning in a wave. Thus, in birds, EDA is recast to a spatiotemporal role that it does not appear to play in other vertebrates, to define when and where patterning should occur in a quasi-1-dimensional manner. The loss of an EDA wave in the ostrich embryo leads to approximately 2-dimensional patterning and a reduction in the hexagonal packing of feathers. The correlation between a functional capacity for flight and the anatomical regularity of feather arrangement that we identify suggests that a highly regular feather pattern is of benefit in flight, perhaps related to aerodynamic efficiency or increased bilateral symmetry. In flightless birds, then, relaxed selection on the arrangement of the plumage has led to the loss of the cellular and molecular waves required for such high-fidelity pattern formation. In the emu, the EDA wave’s guidance of patterning is also lost, but the presumptive feather tracts are still defined molecularly by expression of *EDA* and *CTNNB1* (Fig 7C and S14B Fig and S15 Fig). However, a remarkably low mesenchymal cell density in emu occurs because of a lack of dermatome-derived cell movement towards the midline, resulting in failure of tract patterning (Fig 8C, 8D, 8E and 8F and S14B Fig). The late-arising feathers in the emu embryo are smaller than the tract feather primordia of other species, being instead of the same size as the late-forming duck secondary down feathers (S19 Fig), and first emerge well outside the presumptive tracts.

As the flighted tinamou has the same tract layout as chicken, this basic arrangement of tracts is likely to have been present in the last common ancestor of all living birds. Modifications to this specific layout are present in different species, typically related to the width of the tracts [74], though tract fusion occurs in some species, such as the ostriches reported here. That ostrich and emu have lost the high degree of order of their feather arrangement through different developmental routes agrees with phylogenetic studies indicating that these species have undergone losses of flight independently of one another, rather than from a flightless common ancestor [63]. Thus, either the cellular or the molecular aspects of the feather waves can be altered in the course of evolutionary history, to similar ends.

The structure of the skin, that of a uniform epithelial sheet interacting with underlying mesenchyme of significantly varying cell density, is common to many vertebrate organs. Such epithelial-mesenchymal organs—including the gut, kidney, lung, mammary and salivary glands, and parts of the skeleton during its development—tend to have repeating anatomical units, whether containing discrete elements such as intestinal villi or as a coherent periodic structure such as the epithelial tree of lung or kidney. This composite tissue structure lends itself to either the operation of Turing/reaction-diffusion mechanisms, dominated by relatively stationary epithelial cells, or to cell motility–driven organisation dominated by the mesenchyme. In avian skin, we report a hybrid of these conceptual systems, a reaction-diffusion-taxis mechanism integrated with mechanical processes. These local patterning interactions are triggered within the broad field of the skin by the passing of a travelling EDA wave, imposing the construction of a precise hexagonal lattice of feather primordia.

## Materials and methods

### Ethics statement

All animal work was approved by the Roslin Institute AWERB and the United Kingdom Home Office.

### Eggs and incubation

Dekalb White embryos from Henry Stewart & Co (Norfolk, UK) were used unless stated otherwise. Scaleless eggs were obtained from the University of California Davis Meyer Hall Avian Facility, USA, and incubated at the Roslin Institute. Transgenic green fluorescent (CAG-GFP) [44], TPZ, Chameleon, and MacGreen reporter chicken eggs were obtained from the Roslin Institute National Avian Research Facility (NARF); Khaki Campbell/Indian runner duck eggs were obtained from a small supplier. Ostrich (*Struthio camelus*) and emu (*Dromaius novaehollandiae*) eggs were supplied by Woodbine Farms (Northamptonshire, UK). Chilean tinamou (*N*. *perdicaria*) embryos were provided by the Hancock Wildlife Foundation (Surrey, Canada). Chicken and duck eggs were incubated vertically at 37.8°C with no rotation. Emu and ostrich eggs were incubated horizontally at 36.4°C and rotated 90° periodically until day of extraction. Embryonic developmental stages were scored according to Hamburger and Hamilton for chicken [75] and Hamburger Hamilton equivalent for emu [76].

### Skin explant culture and manipulation of intercellular signalling

Dorsal skin from the base of the skull to tail, comprising the dorsal tract and parts of both left and right femoral tracts, were dissected from embryos of the required developmental stage in PBS and affixed onto nitrocellulose filters (Millipore). For basic culture, explants were submerged in 2 ml of standard medium (DMEM supplemented with 2% FBS and 1% penicillin-streptomycin) or supplemented medium at 37°C, 5% CO_2_.

Recombinant human FGF9, BMP2, BMP4, BMP7, BMP2/7, and BMP4/7 proteins were from R&D Systems. Recombinant human FGF20 protein was obtained from two commercial suppliers, but both had no significant effects on skin development or on expression of the FGF signalling target gene *ETV5* in our hands. Fc-chEDA1, Fc-chEDA2, Ecto-D2, Aprily2, and mAb-EDAR3 were as described [52,54]. Small molecules used were SU5402 (Sigma-Aldrich), LDN193189 (Stemgent), CHIR99021 (Axon Biochem), Latrunculin A (Sigma-Aldrich), and Methotrexate hydrate (Sigma-Aldrich).

### Compressed versus taut skin culture

Skin explants at E6.5 were placed into TRI reagent (Sigma-Aldrich) immediately (as time 0 hour) or cultured for 2 or 4 hours either mounted on a nitrocellulose filter (basic culture method) or free-floating in standard culture medium. Cultured explants were subsequently placed into TRI reagent and processed for qRT-PCR.

### Explant compression culture

Rectangular segments (1 mm wide) were excised from triangular nitrocellulose filters (Millipore), leaving a thin strip of filter to connect the two parts. Dissected E6.5 TPZ dorsal skin explants were mounted to these prepared filters with the dorsal midline directly parallel to the excised section of the filter. Skin was placed across the excised gap and cultured for 2 hours under standard conditions to allow for the adherence of the explant to the filter. A cut was then made perpendicular to the excised section of the filter on the “free-floating” side of the culture (explant is untouched by the cut), separating the two parts of the filter. The “free floating” side of the culture was then manually compressed by narrowing the filter-excised gap. Compressed explants were subsequently cultured over a total period of 48 hours under standard culture conditions.

### Localised delivery of proteins

Affi-Gel Blue Gel beads (Bio-Rad) were washed twice in PBS for 10 minutes and incubated in either recombinant proteins or an equal concentration of BSA diluted in PBS overnight at 4°C. Beads were transferred to nitrocellulose filters, and dissected dorsal skin explants were mounted, dermis side down onto the beads, to the filter. Explants were then cultured using the standard culture method. For Latrunculin A cotreatments, skin explants were cultured for 2 hours prior to the application of protein-loaded beads.

### Time-lapse imaging and PIV analysis

For time-lapse imaging, E6.5 explants prepared from CAG-GFP chicken embryos were mounted onto nitrocellulose filters and cultured skin side down in a 6-well dish containing 2 ml standard or supplemented medium at 37°C, 5% CO_2_. Real-time imaging was performed using the Zeiss Live Cell Observer/Deconvolution system, imaged every 10 minutes for the duration of the experiment, and analysed using ZenBlue2012 (Zeiss) software.

For the PIV analyses [77,78], microscope image frames of skin showing fluorescent (red and green) cells were superposed in one image to achieve higher density. PIV was conducted using MatPIV, implemented in Matlab, with global, local, and signal-to-noise filter. Interrogation areas (32 × 32 pixel size) with 50% overlap were chosen. PIV yields displacement (velocity) fields for every pair of consecutive frames, which were used to derive velocity and angle kymographs as well as streamlines.

### Individual cell tracking

Dorsal skin explants were prepared from transgenic Chameleon E6 chicken embryos and placed onto a nitrocellulose filter. TAT-CRE (62.5 units; Millipore) diluted in phenol red–free DMEM (Sigma-Aldrich) to a final volume of 100 μl was delivered under the dermis. Explants were cultured in a 6-well plate containing 500 μl of phenol red–free medium supplemented with 2% FBS and 1% penicillin-streptomycin for 2 hours at 37°C, 5% CO_2_. Explants were submerged in a total of 2 ml of supplemented phenol red–free medium and cultured for 20 hours. Recombination was confirmed via fluorescence microscopy prior to time-lapse confocal imaging using the Zeiss LSM710 confocal microscope. Images were taken every 15 minutes over a period of 24 hours using the red and green channels. Data were processed using ZenBlue2012 software (Zeiss) followed by cell-tracking analysis (Brownian motion) using IMARIS software.

Automatic cell tracking was performed using Fiji. The cell diameters were estimated for each slice and each signal (green, red) separately. For preprocessing, the images were converted to 8-bit. Salt-and-pepper noise was removed with a median filter (3-pixel kernel). Cell motion analysis was performed for each slice and each signal separately. For each analysis, an image sequence with a spot and track overlay (fast backward tracks and uniform colouring) was saved. Exported files from TrackMate include track and spot statistics. The statistics for the two processed channels of each slice were combined. For time points with missing spots, the positions were interpolated using the mean of the preceding and following time point.

To measure individual cell behaviour specifically in loose dermis or primordium rows before and during condensate formation, manual cell tracking was performed using the Manual Tracking tool in the Fiji distribution version of ImageJ. Maximum-intensity Z-projection of time-lapse images of only the uppermost dermal layer from TAT-Cre-induced Chameleon skin explants were cropped and separated into two regions, loose dermis and primordium row–forming (225-μm-wide blocks). The first row lateral to the primary midline row was designated the row-forming region, whereas loose dermis was the skin region that never experienced condensate formation throughout the imaging period (determined from the Nuclear Blue signal at the end of the time lapse). Three independent skin samples were analysed. Tracking periods were 10 hours before and during primordium formation. Only cells that were tracked for the full duration of each period were included in the analysis.

### Epidermal-dermal recombination

Interspecies epidermal-dermal recombinations were performed on HH29-equivalent embryos. Dissected dorsal skins were incubated in 2X magnesium and calcium-free saline containing 0.25% EDTA for 20 minutes at 37°C followed by separation of the epidermal and dermal layers in PBS. The dermis from one species was placed onto a nitrocellulose filter, and the epidermis from the other species was stretched across the dermis and pinned to the filter. Recombinants were cultured in a 6-well plate with 500 μl of standard medium for 2 hours at 37°C, 5% CO_2_ and then submerged in a total of 2.5 ml of standard medium and cultured for a maximum of 6 days. Medium was replaced every 48 hours.

### In ovo embryo injection

Eggs incubated to E5.5 were windowed and intravenously injected with 200 μg of Aprily2 or Ecto-D2 antibody in PBS. For mAb-EDAR3 treatment, 50 μg of the antibody or an equivalent volume of PBS was intravenously injected into windowed E5.5 eggs. PBS (200 μl) containing 1% penicillin/streptomycin was pipetted over the embryo prior to injection. Glass microcapillaries were loaded with antibody and injected directly into either the left or right vitelline vein using the Femto-Jet4i microinjector. Eggs were sealed and reincubated until the desired stage followed by fixation of embryos in 4% paraformaldehyde (PFA) for processing. For analysis of older embryos (≥E11.5), a second round of injection was performed at E7.5.

### Tissue processing and paraffin sectioning

Skin explant samples or embryos fixed in 4% PFA were washed in PBS and then dehydrated through an ethanol series, followed by incubation in 1:1 (v/v) ethanol:chloroform. Samples were incubated in chloroform overnight prior to embedding in paraffin blocks. Transverse sections (8 μm) were prepared from both embryo and skin explant samples.

### Tissue processing and cryosectioning

Hetero-specific epidermal-dermal recombinant and TAT-Cre-induced Chameleon explants were fixed in 4% PFA, embedded in 15% sucrose/7.5% gelatin in 0.12 M sodium phosphate buffer, and cryosectioned at 20 μm. Gelatin was removed by washing in prewarmed (37°C) PBS. Recombinant sections were stained with DAPI mounted in ProLong Gold antifade reagent (Life Technologies) and imaged with an inverted confocal microscope (Zeiss LSM 710) using ZenBlue2012 software (Zeiss).

### Immunodetection

Sections were dewaxed in xylene, rehydrated into water through an ethanol series, and processed for antigen retrieval in the Bio-retriever2000 in either EDTA buffer or citrate buffer. Sections were washed in TBST, incubated in blocking buffer containing 5% goat serum in TBST for 2 hours at room temperature, and then incubated overnight at 4°C in TWIST1/2 antibody (GenTex) at 1/200 or phospho-Histone H3 (Ser10) antibody (Cell Signaling Technology) at 1/200 in blocking buffer. Sections were washed in TBST followed by incubation in biotinylated anti-rabbit secondary antibody (1/200 in blocking buffer) for 1.5 hours at room temperature. Sections were then washed in TBST and incubated at room temperature for 1 hour in HRP-conjugated streptavidin 1/200, followed by another TBST wash. Sections were stained in DAB solution until colour appeared and were washed in water for 20 minutes. DAB-stained sections were lightly counterstained with haematoxylin prior to mounting in hydromount. Images were collected by automated slide scanning (NanoZoomer, Hamamatsu).

For immunofluorescence, after removal of the HRP-conjugated streptavidin, Tryamide signal amplification was performed using TSA kit #26 Alexa fluor 647 (Thermo Fisher) for 10 minutes at room temperature as per manufactures guidelines. Sections were then counterstained with DAPI, mounted in ProLong Gold antifade reagent (Life Technologies), and imaged with an inverted confocal microscope (Zeiss LSM 710) using ZenBlue2012 software (Zeiss).

### RNA in situ hybridisation

Cultured skin explants or embryos were fixed overnight in 4% PFA at 4°C, dehydrated through a methanol series, bleached in 5% hydrogen peroxide (H_2_O_2_), and then rehydrated into PBS. Samples were treated with 20 μg/ml proteinase K, postfixed in 4% PFA containing 0.2% glutaraldehyde, and hybridised with a digoxigenin (DIG)-labelled riboprobe for single in situ hybridisation or in combination with a 2, 4-dinitrophenyl (DNP)-labelled riboprobe for double in situ hybridisation. Unbound probe was removed via washing prior to DIG-labelled probe detection using 1/1,000 alkaline phosphatase (AP)-conjugated anti-DIG antibody (Roche) followed by signal detection using 5-bromo-4-chloro-3-indolyl phosphate/nitroblue tetrazolium (BCIP/NBT).

For double in situ hybridisation, after the first colour reaction, samples were postfixed in 4% PFA for 30 minutes at room temperature. DNP-labelled riboprobe was detected using 1/1,000 AP-conjugated anti-DNP antibody (Vector) and then coloured using BCIP/2-(4-iodophenyl)-3-(4-nitrophenyl)-5-phenyltetrazolium chloride (INT).

### Analysis of sectioned tissue samples

For DAPI-stained samples, 0.71-μm optical slices were taken using an inverted confocal microscope (Zeiss LSM 710) with ZenBlue2012 software (Zeiss). Samples that had undergone in situ hybridisation were imaged with a stereo microscope (Olympus SZX10) using cell^B (Olympus, Tokyo, Japan) software. Images were analysed using Image-Pro Plus software (Media Cybernetics).

For determination of dermal cell density threshold permissive to feather patterning, *FGF20* expression was detected in control, Fc-chEDA1-treated, and Ecto-D2-treated skin explants by in situ hybridisation. An incision was made on each explant at the margin of the *FGF20*-expressing regions of the dorsal tract using a razor blade. Samples were processed for paraffin sectioning, and sections were dewaxed in xylene followed by rehydration through an ethanol series into water. Sections were stained with DAPI and mounted in ProLong Gold antifade reagent (Life Technologies). Dermal cells were quantified from the skin regions immediately adjacent to the cut site in the *FGF20*-negative region (site of next presumptive primordium row). All dermal cells within the first 100 μm from the cut were counted from every sample and averaged.

For dermal cell density quantification, all sections analysed were taken from the posterior dorsal region of the embryo, between the femoral tracts. Sections were dewaxed and rehydrated followed by DAPI staining and mounted in ProLong Gold antifade reagent. Dorsal tracts were partitioned into 100 × 41-μm blocks for sections from whole embryos or 100-μm sections for skin explant sections across the entire presumptive tract, below the epidermis and beginning from the dorsal midline. Dermal cell numbers were quantified for each block across the entire dorsal tract for individual samples and then averaged across each treatment group/embryonic stage.

### Pattern analyses

Measurements of the width of the patterning region were performed on cultured skin explants or whole-mount embryos that were subjected to in situ hybridisation for *FGF20* detection. The pattern-generating region of the dorsal tract on the explants was defined by *FGF20* expression. Three measurements of the width of the *FGF20* pattern were made for each skin and averaged, with a minimum of three skins per treatment.

For pattern fidelity analysis, explant or whole-mount samples processed for *CTNNB1* detection through in situ hybridisation were imaged. Primordia from the dorsal tracts were outlined and given a binary mask, for which a 1 indicated the location of the primordium and a 0 indicated the interprimordium region (see Fig 2C). The centroid of each primordium was then extracted, and a Voronoi diagram was constructed from the point cloud data. The Voronoi diagram partitions the plane into regions based on the distance to the primordium centroids. Specifically, a primordium’s defined region consists of all spatial points closer to its centroid than to any other. These regions are called Voronoi cells. The Voronoi diagram has the property that the boundary cells are anomalous, with infinite (or exceedingly large) area. In order to remove such artefacts, we removed the most external primordium centroids from consideration within the data until the area of all Voronoi cells was less than three standard deviations from the mean. In practice, this eliminated only the anomalous cells. Once the appropriate cells had been chosen, statistics were autonomously extracted regarding the number of nearest neighbours of each primordium (Fig 2D, Fig 7B, S17B Fig). Specifically, if the primordia were perfectly aligned on a (nonsquare) grid, each placode would have six neighbours (and four neighbours in the singular case of a square grid). Thus, the spread and range away from six neighbours illustrates the variability in the pattern fidelity. Namely, a higher percentage of primordia with six neighbours implies a more regular placode pattern.

### Visualisation of tinamou feather arrangement

E14 tinamou and chicken embryos were fixed in 4% PFA. Embryos were washed in PBS, and all feather filaments were cut off manually using fine scissors. Embryos were submerged in haematoxylin for 30 seconds, followed by PBS washes, and imaged with a stereo microscope (Olympus SZX10) using cell^B (Olympus, Tokyo, Japan).

### qRT-PCR

Total RNA was isolated using TRI reagent and reverse transcribed using random hexamers and Superscript III Reverse Transcriptase (Invitrogen) in a final volume of 20 μl. cDNA was diluted 10-fold, 3 μl of which was used as template for each qRT-PCR. qRT-PCRs were performed using SYBR Green Universal Master Mix (Roche) in a final volume of 20 μl, and each was performed in triplicate, with a minimum of three biological repeats. A dilution standard curve was used to determine the relative expression levels.

Primer Sequences:

*AXIN2* F: 5′-GCCGACTTCAAAAGCAAAAT-3′

*AXIN2* R: 5′-GTAGGGGTTAACGGGATCGT-3′

*BMP2* F: 5′-CCAACACCGTGTGCAGCTTCCA-3′

*BMP2* R: 5′-GAAACGTCGTGCTGTTTTCCCAC-3′

*BMP4* F: 5′-TGAGGAGCTTCCACCATGA-3′

*BMP4* R: 5′-TGCTGAGGTTGAAGACGAAG-3′

*BMP7* F: 5′-CGAACGCTTCGATAATGAAA-3′

*BMP7* R: 5′-TTCTGGAGTCGAGCAGGAAC-3′

*EDA* F: 5′-TGGTCTCGCATCACTATGAAC-3′

*EDA* R: 5′-AATACTCCGAGTGCATTGCAG-3′

*EDAR* F: 5′-TTCTTTCGAGCCACTGTCCT-3′

*EDAR* R: 5′-CGAGGTCTGTTTTCCAGCAT-3′

*ETV5* F: 5′-AGGCCTGGCTAGCTGAAGCTCA-3′

*ETV5* R: 5′-ATCTTGGCTGGAGGTGGGGCAT-3′

*FGF20* F: 5′-GCCAAGACCACAGCCTCTT-3′

*FGF20* R: 5′-TTCCAAGGTAAAGGCCACTG-3′

*GAPDH* F: 5′-GACAACTTTGGCATTGTGGA-3′

*GAPDH* R: 5′-GGCTGTGATGGCATGGAC-3′

## Supporting information

S1 Fig Effects of FGF and BMP signal perturbation on feather primordium formation and relative timing of *FGF20* expression with cell condensation. (A) Time series of primordium development in CAG-GFP skin, beginning from E7.5, over 20 hours. Scale bar: 1 mm. (B) Effect of SU5402, an inhibitor of FGF receptor signalling, treatment on E6.5 skin explants after 24 and 48 hours in culture, as assessed by detection of cell density using CAG-GFP transgenic skin. Scale bar: 1 mm. (C) Effect of two BMP4 treatment doses on E6.5 skin explants after 24 hours in culture, assessed by *FGF20* expression. Scale bar: 1 mm. (D) Dose effects of BMP4/7 heterodimer, BMP2, and BMP7 on E6.5 skin explants after 48 hours in culture on primordium row formation, assessed by *CTNNB1* expression. Scale bar: 1 mm. (E, F) Effects of LDN193189 (BMP inhibitor) treatment on E6.5 skin explants up to 48 hours in culture, assessed by *FGF20* expression (E) and by detection of cell density using CAG-GFP transgenic skin (F). Scale bars: 1 mm. (G) E6.5 GFP skin explants cotreated with FGF9-coated beads and BMP4-supplemented medium cultured over 48 hours. Scale bar: 500 μm. (H) Skin from CAG-GFP embryos cultured from E6.5 for up to 44 hours and imaged to detect GFP (below), followed by detection of *FGF20* expression in the same sample (above). Establishment of *FGF20* gene expression coincides with the formation of mesenchymal cell aggregates at all developmental stages. Faint *FGF20* signals overlap with newly condensing and unresolved mesenchymal cell aggregates (arrowheads). Scale bar: 1 mm. BMP, bone morphogenetic protein; E, embryonic day; FGF, fibroblast growth factor; GFP, green fluorescent protein.
(TIF)

S2 Fig Assessment of regulation of patterning genes. (A) qRT-PCR detecting *ETV5*, *BMP2*, *BMP4*, and *BMP7* expression in E6.5 skin explants cultured with 1 μg/ml FGF9 for 5 hours. *ETV5* is a positive control, representing a general FGF target gene. Statistical significance from control was calculated using Student *t* test, (*p < 0.05). (B) qRT-PCR detecting *AXIN2*, *BMP2*, *BMP4*, *EDA*, and *EDAR* expression in E6.5 skin explants either cultured with an underlying filter or free-floating after 2 or 4 hours in culture. T0 controls were freshly dissected from embryos to determine initial levels of gene expression. Red lines denote the mean and shapes denote values for individual skin samples. The numerical values for A and B can be found in S9 Data. E, embryonic day; FGF, fibroblast growth factor; qRT-PCR, quantitative reverse transcription PCR.
(TIF)

S3 Fig Skin compression does not initiate the wave of feather primordium formation. (A) Schematic of experimental approach. Skin explants were placed with the midline parallel to the edge of a gap in the underlying filter support. This creates a culture condition in which slightly more than one-half of the skin is attached to a filter substrate, and the remainder of the presumptive tract is unattached. (B) E6.5 skin explants prepared from tdTomato transgenic chicken embryos cultured for 2 hours over nitrocellulose filters with an excised section (dotted white line). (B’) After 2 hours in culture, the explant was compressed by physical manipulation of the nitrocellulose filter (indicated by the change of shape in the dotted white line). (C) Over 48 hours of observation, the endogenous travelling wave of primordium formation, initiating at the midline, sweeps symmetrically across both compressed and taut sides of the skin. Scale bar: 1 mm. E, embryonic day.
(TIF)

S4 Fig Induction of *FGF20* expression in a wave by EDA and β-catenin signalling. (A) Detection of *FGF20* in E6.5 explants cultured for 24 hours. A stripe of faint expression is seen ahead of the most recently defined feather row on each side. (B) qRT-PCR detecting *FGF20* expression in E6.5 skin explants cultured with either 30 μM CHIR99021 or 500 ng/ml Fc-chEDA1 (activators of WNT/β-catenin and EDAR pathways, respectively) for 5 hours. Statistical significance from control was calculated using a Student *t* test, (***p < 0.001). (C) qRT-PCR detecting *EDAR* expression in E6.5 explants cultured with 30 μM CHIR99021 for 5 hours. Statistical significance from control was calculated using a Student *t* test, (***p < 0.001). (D) From the initial site of primordium formation (arrow), a spreading wave of *EDA* expression is observed in the developing femoral tracts of chicken embryos. Scale bars: 1 mm. The numerical values for B and C can be found in S10 Data. E, embryonic day; EDA, Ectodysplasin A; EDAR, EDA receptor; qRT-PCR, quantitative reverse transcription PCR.
(TIF)

S5 Fig An expanding wave of *EDA* and a receding wave of *CTNNB1* expression persist in the absence of feather patterning. *EDA* and *CTNNB1* expression in E8 and E9 *FGF20*^*sc/sc*^ (i.e., scaleless mutant) embryos. The embryos (dorsal and lateral views) exhibit expansion of *EDA* expression despite the absence of feather primordium formation. *CTNNB1* expression becomes restricted to the edges of the presumptive tracts, which have failed to undergo patterning. Scale bar: 5 mm. E, embryonic day.
(TIF)

S6 Fig Effects of in ovo inhibition of *EDA* signalling on feather tracts. (A) Ventral, (B) lateral, and (C) head views of E8.5 control antibody (Aprily2) and Ecto-D2 injected embryos, treated at E5.5. Inhibition of EDA signalling reduces the extent of primordium formation in every tract compared to controls after 72 hours of treatment. Scale bars: 2 mm. E, embryonic day; EDA, Ectodysplasin A.
(TIF)

S7 Fig In ovo inhibition of EDA signalling leads to loss of scleral papilla formation. Scleral papillae, visible as a ring of discrete cell condensates within the eye, fail to form in embryos treated in ovo with Ecto-D2 from E5.5 and collected at E8.5. Reduced scleral papilla formation is also observed in developing *FGF20*^*sc/sc*^ embryos. Scale bar: 1 mm. E, embryonic day; EDA, Ectodysplasin A.
(TIF)

S8 Fig Effects of in ovo inhibition of EDA signalling on feather primordium stability and growth. (A) Continuous inhibition of EDA signalling from E5.5 to E11.5 or E13.5 results in the absence of feather formation and almost bare skin, as defined by *CTNNB1* and *SHH* expression. Scale bar: 5 mm. (B) Transverse sections of embryos in (A) reveal loss of mesenchymal condensates in the dorsal tract. Black dotted frame in (A) denotes skin region sectioned. Scale bar: 100 μm. (C) Focalisation of *FGF20* expression to the anterior pole within primordia is attenuated at E8.5 when EDA signalling is blocked from E5.5. Scale bar: 1 mm. (D) Mesenchymal condensates are present in E8.5 skin treated in ovo with Ecto-D2 from E5.5. These must regress to yield bare skin at E11.5 and E13.5. Dotted lines demarcate epidermal-dermal junctions. Scale bar: 100 μm. E, embryonic day; EDA, Ectodysplasin A.
(TIF)

S9 Fig Effects of ex vivo or in vivo application of EDAR-stimulating antibody on feather pattern. (A) Comparison of the width of the patterned region, defined by *FGF20* expression, in E6.5 skin explants cultured for 24 hours in the presence of 2 μg/ml mAb-EDAR3 (an activator of EDAR signalling). (B) Quantification of patterned region width between control and mAb-EDAR3-treated explants. Statistical significance was calculated using a Student *t* test (**p < 0.01). (C) Width of patterned region, defined by *FGF20* expression, is increased in mAb-EDAR3-injected E7.5 embryos compared to control embryos. (D) Quantification of width of primordium generating regions in mAb-EDAR3 in ovo–treated embryos compared to their respective controls. Statistical significance from control was calculated using a Student *t* test (*p < 0.05). Scale bars: 2 mm. The numerical values for B and D can be found in S11 Data. E, embryonic day; EDAR, Ectodysplasin A receptor.
(TIF)

S10 Fig Mesenchymal cell dynamics tracked in somite-transplanted embryos. (A) A GFP-tdTomato somite chimeric embryo was generated using a TPZ donor and CAG-GFP host. Somites 30–32 from an HH17 TPZ donor embryo were transplanted to an equivalent region in an HH15 CAG-GFP host, which was then allowed to develop to E6.5. (B) Dorsal skin from the E6.5 chimeric CAG-GFP embryo with transplanted TPZ somites was cultured and imaged in real time. (C) GFP images and tdTomato images obtained from cultured chimeric skin (far left and far right), with X-velocity kymograph (average x-direction speed towards or away from the midline) from each channel (GFP and tdTomato) generated through PIV analysis of the real-time videos. Cell behaviour in the tdTomato half-skin shows the same behaviour as intact CAG-GFP skin, showing that intact skin imaging reflects mesenchymal cell dynamics. Scale bars: 500 μm. E, embryonic day; GFP, green fluorescent protein; HH, Hamburger Hamilton stage; PIV, particle image velocimetry.
(TIF)

S11 Fig MTX treatment reduces mesenchymal cell density in cultured skin. Quantification of mesenchymal cell densities, visualised by DAPI staining of tissue sections, across cultures of E6.5 dorsal skin maintained for 48 hours in the presence or absence of 5 μM MTX, an inhibitor of cell proliferation. The numerical values for the figure can be found in S12 Data. E, embryonic day; MTX, methotrexate.
(TIF)

S12 Fig Tracking of individual cell movement in sparsely labelled Chameleon skin. (A) Angle of movement relative to anterior-posterior axis over time calculated for each z-plane optical layer (1: most superficial epidermis; 6: deepest layer mesenchyme) from 2-dimensional single-cell tracking analysis. Cell movement in epithelial layers is more coordinated (indicated by broader blocks of the same colour) and remains constant over greater time periods (indicated by little y-axis colour change) than in the mesenchyme, where mixed colours in deeper layers indicate different directions of cell movement in immediately neighbouring regions. (B) Plot of the percentage of mesenchymal cells moving in a positive (upwards) and a negative (downwards) z-direction. “Strong” movement indicates movement out of one confocal plane and into a neighbouring one (on average 12 μm). Error bars indicate S.E.M. (C) Angle plot showing the time-averaged movement direction (angle) and time-averaged velocity (line length) for every cell in all z-plane layers derived from 2-dimensional cell-tracking analysis for each slice of each of the three time-lapse movies. Medial is to the left and anterior to the top. Individual cell movement occurs almost entirely towards the midline. The numerical values for B and C can be found in S13 Data.
(TIF)

S13 Fig Individual mesenchymal characteristics in dense and loose dermis. (A) Cell velocity and (B) persistence of mesenchymal cell movement in the 10 hours before and the 10 hours during primordium row formation in the dense mesenchyme (Row) and same time period in the loose mesenchyme (Loose) in TAT-Cre induced Chameleon skin. Persistence of cell movement is highest during the medial migration of cells before initiation of condensate formation along a row. Each coloured circle represents the mean value from a single skin sample. (C) High-resolution images of labelled (red) and unlabelled (Nuclear Blue) Chameleon cells from loose dermis and dense dermis and within a primordium. (D) GFP-expressing macrophages in the skin prepared from a MacGreen reporter chicken display cell morphology that is distinct from that of the labelled Chameleon cells. Scale bars: 50 μm. The numerical values for A and B can be found in S14 Data. GFP, green fluorescent protein.
(TIF)

S14 Fig Feather pattern formation in ratite embryos. Dorsal and lateral views of primordium pattern formation through in situ hybridisation to detect *CTNNB1* transcripts in developing (A) ostrich and (B) emu embryos. Prior to the appearance of primordia, both species display a predefined *CTNNB1-*expressing dorsal tract; however, a medial-lateral patterning wave is absent. Ostrich embryos pattern within the tract region when and where cell density increases (see corresponding skin sections stained with DAPI to right). Emu embryos fail to attain high mesenchymal cell density while *CTNNB1* expression defines the tract, instead producing later feather primordia in lateral regions with high cell density. + indicates skin from a region undergoing feather primordium formation;—indicates skin from a region not undergoing feather primordium formation. Scale bar: 5 mm for embryos; 50 μm for skin sections.
(TIF)

S15 Fig *EDA* expression in emu and ostrich embryos. Emu and ostrich embryos express *EDA* in the dorsal skin corresponding to the tract region, prior to feather formation in both species. Scale bars: 5 mm.
(TIF)

S16 Fig Feather tract arrangements in different species. Comparisons of feather distributions between E9.5 chicken, E10 duck, E18 ostrich, and E21 emu. Feather primordia are visualised by detection of the *CTNNB1* transcript. Matching feather tracts in chicken, duck, and ostrich can be identified by the presence of the featherless (apteric) regions separating neighbouring feather tracts. However, distinct feather tracts cannot be identified in emu embryos. Instead, almost the entirety of the embryo is covered in small feather primordia. Scale bar: 5 mm. E, embryonic day.
(TIF)

S17 Fig Tinamou, a flighted ratite, has feather tracts and hexagonal arrangements of feather primordia. (A) E12 chicken and E14 tinamou embryos were processed for haematoxylin staining to aid visualisation of feather arrangements in the developing embryos. Feather filaments were cut off and embryos dipped in haematoxylin to stain the remaining shaft. Comparisons of the dorsal and lateral sides of the embryos show that the shape of the individual tracts differ between species, but the basic layout of tracts is the same. Scale bar: 5 mm. (B) Voronoi tessellation analysis of images of chicken and tinamou dorsal tracts reveal that tinamou feathers are laid out in a hexagonal lattice that is comparable to the arrangement in chicken. The numerical values for B can be found in S15 Data. E, embryonic day.
(TIF)

S18 Fig Failure of presumptive dorsal tract patterning in emu and late onset densification of mesenchyme. (A) Double in situ hybridisation to detect *EDA* (purple) and *CTNNB1* (orange) transcripts in an E18 emu dorsal tract reveal that primordium formation (*CTNNB1* positive foci) is initially absent from regions expressing *EDA*. Scale bar: 2 mm. (B) Immunohistochemical staining for TWIST1/2 proteins (nuclear marker of mesenchymal cells) in transverse sections of dorsal skin from HH23, HH26, and HH29 chicken and emu embryos. Magnified sections from these images are shown in Fig 8. Scale bar: 100 μm. (C) Immunohistochemical detection of phospho-Histone H3 (Ser10) in transverse sections of dorsal HH21 chicken and emu embryos. Dotted lines denote the dermomyotomal lip. Scale bar: 100 μm. E, embryonic day; HH, Hamburger Hamilton stage.
(TIF)

S19 Fig Duck secondary primordium formation and comparison of feather primordium sizes between species. (A) Secondary primordium formation in E10 to E12 duck embryos detected by in situ hybridisation for *CTNNB1* expression. At day 10, only the primary feather primordia are visible, with no intervening *CTNNB1* expression. At day 11, *CTNNB1* expression appears between the existing primary feather primordia. By day 12, secondary feather primordia are resolving between the outgrowing primary feathers. Scale bar: 1 mm. (B) *SHH* in situ detection of secondary feather primordia, visible as small dots between primary feathers in an E14 duck embryo. The primary feathers at this age each have several stripes of *SHH* expression related to internal branching of the filament. Scale bar: 1 mm. (C) Primordium size distribution in chicken, duck, emu, and ostrich embryos. Primordium surface areas were grouped into 5,000-μm^2^ bins, and the percentage of the primordia within each bin was plotted. The numerical values for C can be found in S16 Data. E, embryonic day.
(TIF)

S1 Video Live cell imaging of dorsal tract patterning in a CAG-GFP skin explant. Time-lapse video of cultured E6.5 CAG-GFP dorsal skin explant developing over a period of 70 hours. Scale bar: 500 μm. E, embryonic day.
(AVI)

S2 Video Live cell imaging of chimeric GFP-tdTomato skin explant. Time-lapse video of cultured E6.5 CAG-GFP dorsal skin explant (green) containing, on the right side, mesenchyme from a donor tdTomato somite (red). Development of the skin was observed over a period of 63 hours. Top: green channel, middle: red channel, bottom: merged. Scale bar: 500 μm. E, embryonic day.
(WMV)

S3 Video Live cell imaging of epithelial cell movement in a TAT-Cre-induced Chameleon skin explant. Representative confocal time-lapse video of labelled and cultured E6.5 Chameleon dorsal skin explant over a period of 9 hours. This z-plane is focussed on epithelial (epidermal) cells. Green and red colours indicate cells that have undergone random recombination at the transgene locus, permitting their visualisation. Top: green channel, middle: red channel, bottom: merged. Arrowheads indicate the position of the midline. Scale bar: 200 μm. E, embryonic day.
(WMV)

S4 Video Live cell imaging of mesenchymal cell movement in a TAT-Cre-induced Chameleon skin explant. Representative confocal time-lapse video of labelled and cultured E6.5 Chameleon dorsal skin explant over a period of 9 hours. This z-plane is focussed on dermal mesenchymal cells. Green and red colours indicate cells that have undergone random recombination at the transgene locus, permitting their visualisation. Top: green channel, middle: red channel, bottom: merged. Arrowheads indicate the position of the midline. Scale bar: 200 μm. E, embryonic day.
(WMV)

S1 Data Data pertaining to Fig 1D, 1E and 1H. (XLSX)

S2 Data Data pertaining to Fig 2D. (XLSX)

S3 Data Data pertaining to Fig 3C, 3E, 3G and 3I. (XLSX)

S4 Data Data pertaining to Fig 4B. (XLSX)

S5 Data Data pertaining to Fig 5C, 5D, 5F and 5G. (XLSX)

S6 Data Data pertaining to Fig 6D and 6F. (XLSX)

S7 Data Data pertaining to Fig 7B. (XLSX)

S8 Data Data pertaining to Fig 8E and 8F. (XLSX)

S9 Data Data pertaining to S2A and S2B Fig. (XLSX)

S10 Data Data pertaining to S4B and S4C Fig. (XLSX)

S11 Data Data pertaining to S9B and S9D Fig. (XLSX)

S12 Data Data pertaining to S11 Fig. (XLSX)

S13 Data Data pertaining to S12B and S12C Fig. (XLSX)

S14 Data Data pertaining to S13A and S13B Fig. (XLSX)

S15 Data Data pertaining to S17B Fig. (XLSX)

S16 Data Data pertaining to S19C Fig. (XLSX)

S1 Supporting Methods Generation of the tdTomato (TPZ) chicken line. (DOCX)

S2 Supporting Methods Mathematical methods. (DOCX)

## Abbreviations

a.u.: arbitrary units
BMP: bone morphogenetic protein
E: embryonic day
EDA: Ectodysplasin A
EDAR: Ectodysplasin A receptor
eYFP: enhanced yellow fluorescent protein
FGF: fibroblast growth factor
GFP: green fluorescent protein
HH: Hamburger Hamilton stage
MTX: methotrexate
PIV: particle image velocimetry
qRT-PCR: quantitative reverse transcription PCR
