# Supplementary figures and images for: Feather arrays are patterned by interacting signalling and cell density waves
Source: PLoS Biol. 2019 Feb 21;17(2):e3000132. doi: 10.1371/journal.pbio.3000132 (PMC6383868; doi:10.1371/journal.pbio.3000132)

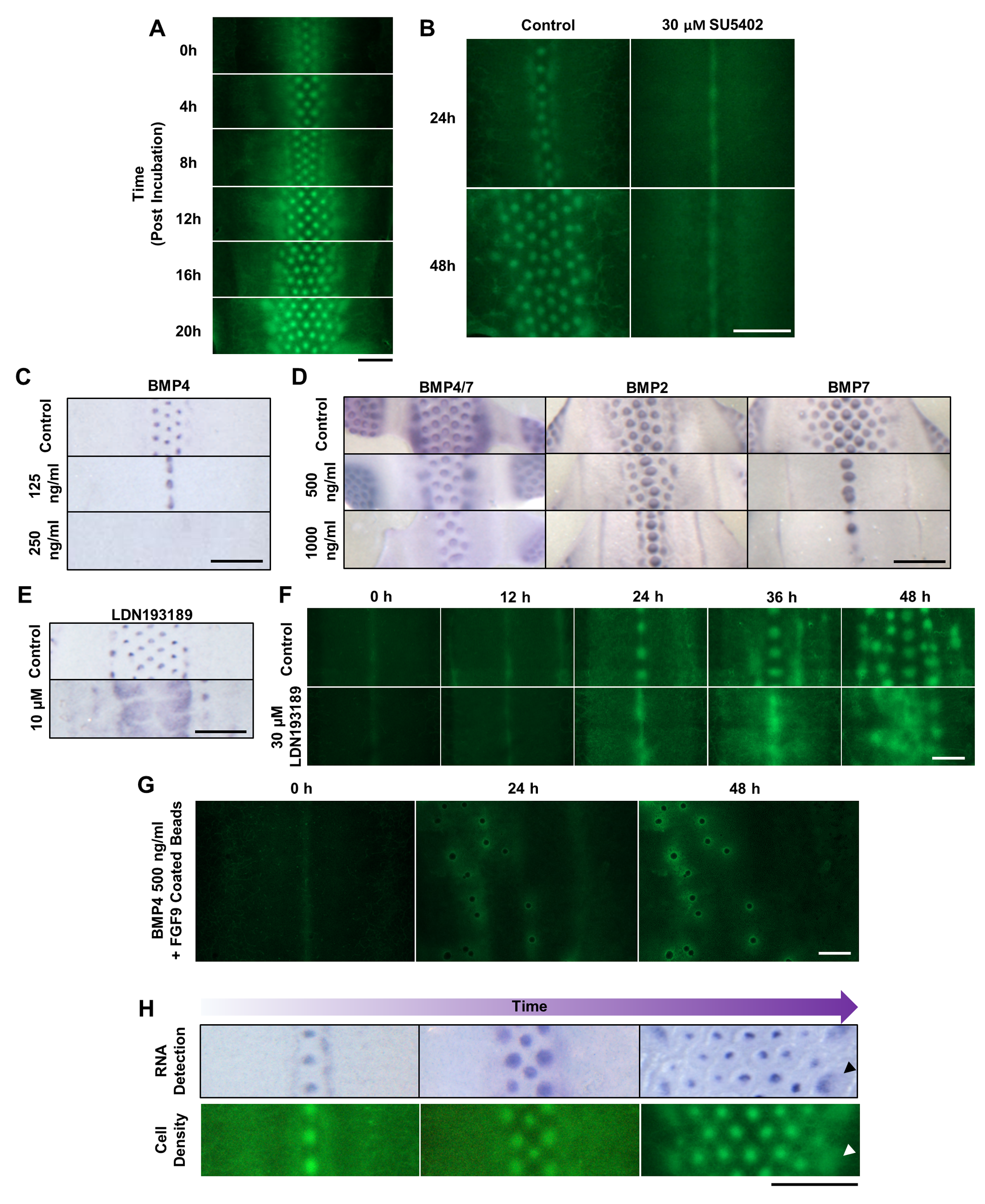

Supplement: S1 Fig — (A) Time series of primordium development in CAG-GFP skin, beginning from E7.5, over 20 hours. Scale bar: 1 mm. (B) Effect of SU5402, an inhibitor of FGF receptor signalling, treatment on E6.5 skin explants after 24 and 48 hours in culture, as assessed by detection of cell density using CAG-GFP transgenic skin. Scale bar: 1 mm. (C) Effect of two BMP4 treatment doses on E6.5 skin explants after 24 hours in culture, assessed by FGF20 expression. Scale bar: 1 mm. (D) Dose effects of BMP4/7 heterodimer, BMP2, and BMP7 on E6.5 skin explants after 48 hours in culture on primordium row formation, assessed by CTNNB1 expression. Scale bar: 1 mm. (E, F) Effects of LDN193189 (BMP inhibitor) treatment on E6.5 skin explants up to 48 hours in culture, assessed by FGF20 expression (E) and by detection of cell density using CAG-GFP transgenic skin (F). Scale bars: 1 mm. (G) E6.5 GFP skin explants cotreated with FGF9-coated beads and BMP4-supplemented medium cultured over 48 hours. Scale bar: 500 μm. (H) Skin from CAG-GFP embryos cultured from E6.5 for up to 44 hours and imaged to detect GFP (below), followed by detection of FGF20 expression in the same sample (above). Establishment of FGF20 gene expression coincides with the formation of mesenchymal cell aggregates at all developmental stages. Faint FGF20 signals overlap with newly condensing and unresolved mesenchymal cell aggregates (arrowheads). Scale bar: 1 mm. BMP, bone morphogenetic protein; E, embryonic day; FGF, fibroblast growth factor; GFP, green fluorescent protein. (TIF) [file pbio.3000132.s001.tif]

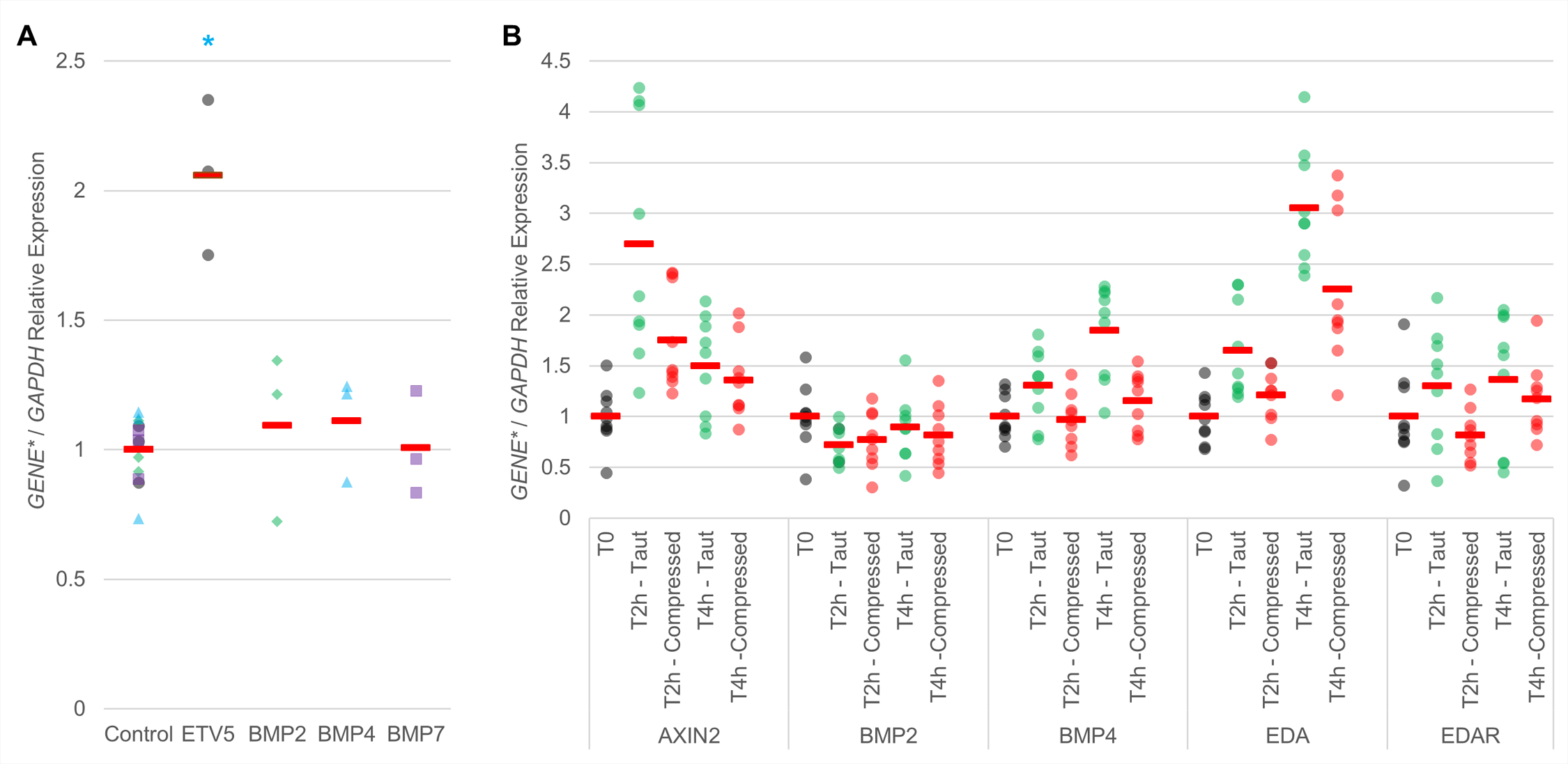

Supplement: S2 Fig — (A) qRT-PCR detecting ETV5, BMP2, BMP4, and BMP7 expression in E6.5 skin explants cultured with 1 μg/ml FGF9 for 5 hours. ETV5 is a positive control, representing a general FGF target gene. Statistical significance from control was calculated using Student t test, (*p < 0.05). (B) qRT-PCR detecting AXIN2, BMP2, BMP4, EDA, and EDAR expression in E6.5 skin explants either cultured with an underlying filter or free-floating after 2 or 4 hours in culture. T0 controls were freshly dissected from embryos to determine initial levels of gene expression. Red lines denote the mean and shapes denote values for individual skin samples. The numerical values for A and B can be found in S9 Data. E, embryonic day; FGF, fibroblast growth factor; qRT-PCR, quantitative reverse transcription PCR. (TIF) [file pbio.3000132.s002.tif]

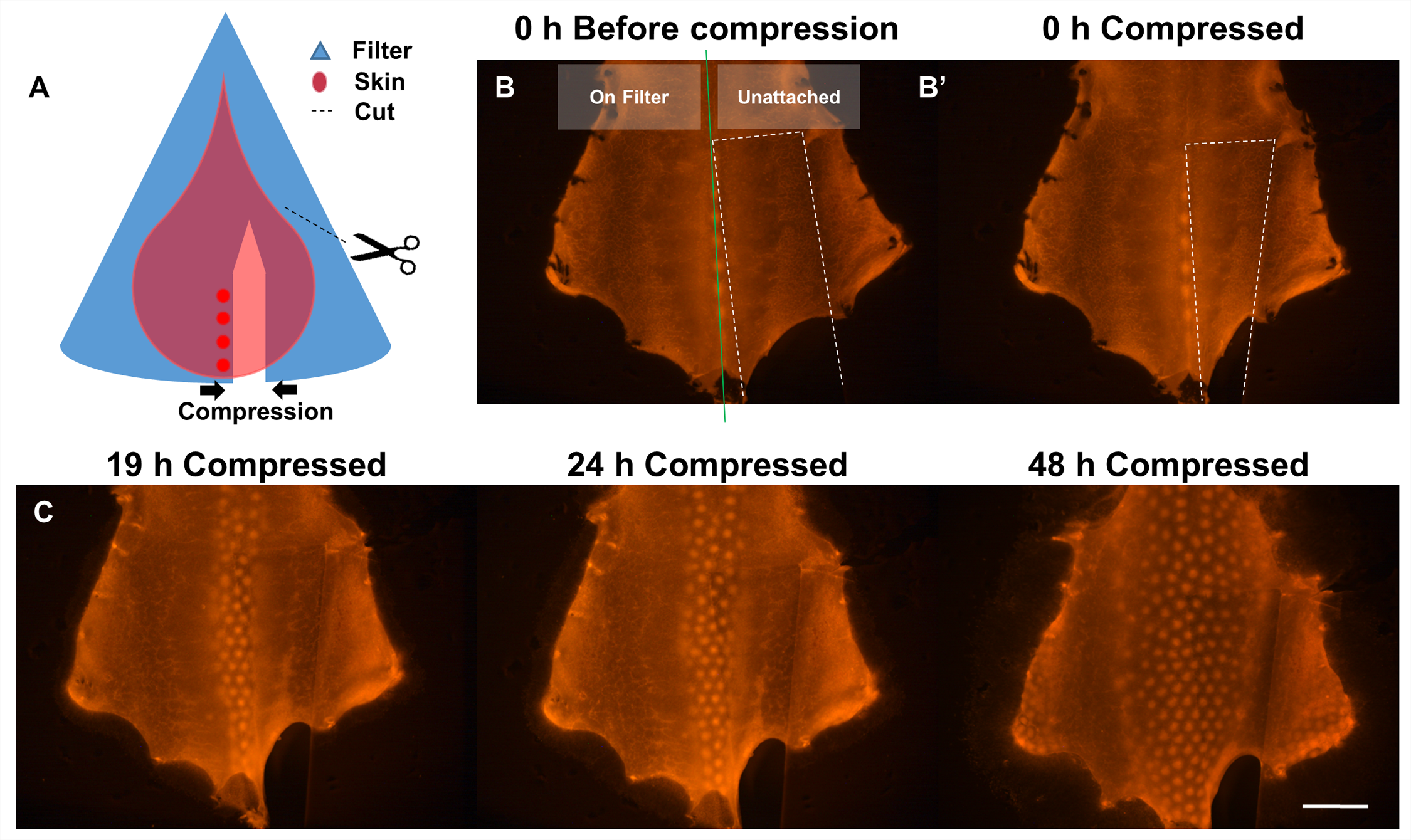

Supplement: S3 Fig — (A) Schematic of experimental approach. Skin explants were placed with the midline parallel to the edge of a gap in the underlying filter support. This creates a culture condition in which slightly more than one-half of the skin is attached to a filter substrate, and the remainder of the presumptive tract is unattached. (B) E6.5 skin explants prepared from tdTomato transgenic chicken embryos cultured for 2 hours over nitrocellulose filters with an excised section (dotted white line). (B’) After 2 hours in culture, the explant was compressed by physical manipulation of the nitrocellulose filter (indicated by the change of shape in the dotted white line). (C) Over 48 hours of observation, the endogenous travelling wave of primordium formation, initiating at the midline, sweeps symmetrically across both compressed and taut sides of the skin. Scale bar: 1 mm. E, embryonic day. (TIF) [file pbio.3000132.s003.tif]

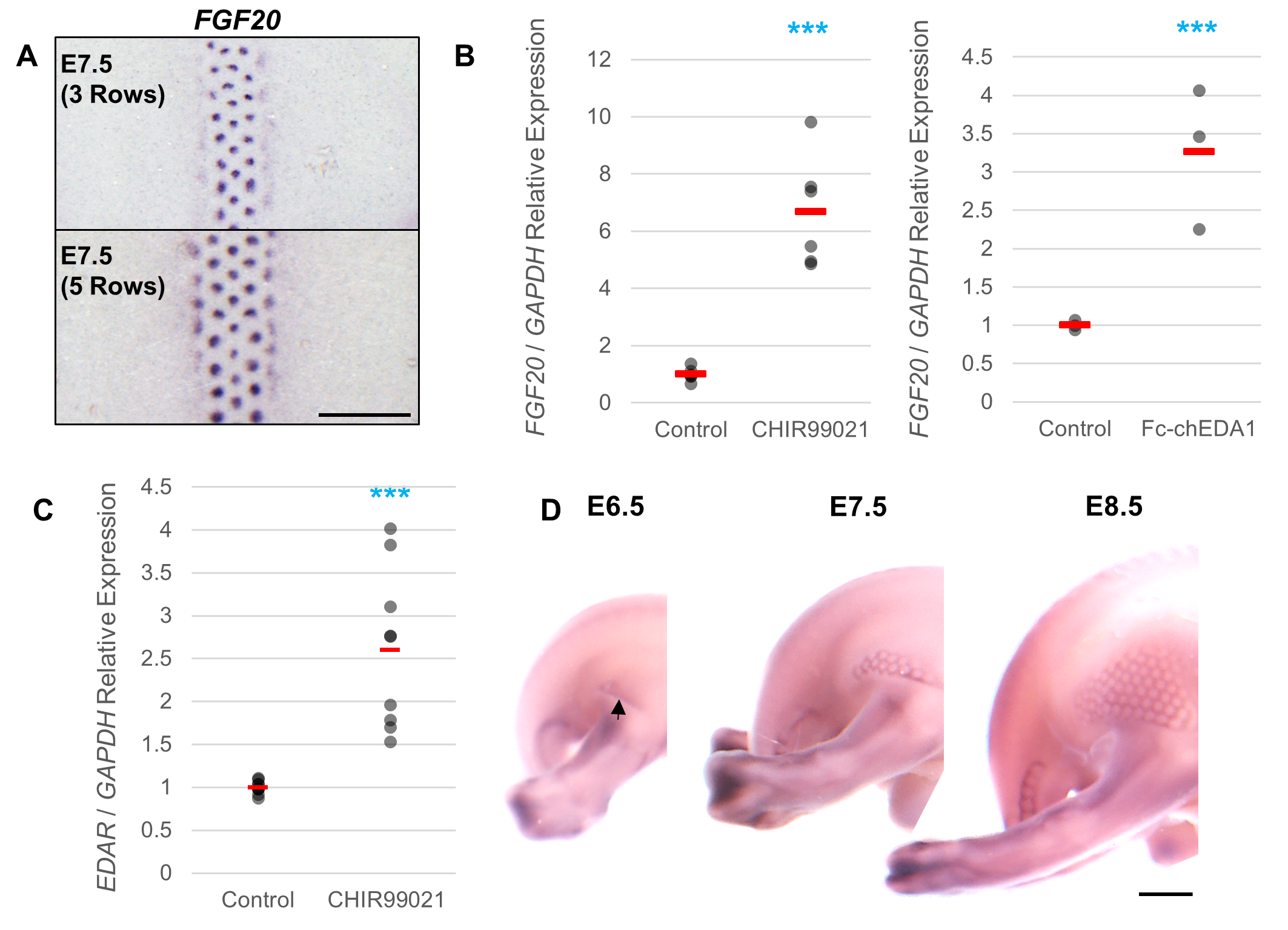

Supplement: S4 Fig — (A) Detection of FGF20 in E6.5 explants cultured for 24 hours. A stripe of faint expression is seen ahead of the most recently defined feather row on each side. (B) qRT-PCR detecting FGF20 expression in E6.5 skin explants cultured with either 30 μM CHIR99021 or 500 ng/ml Fc-chEDA1 (activators of WNT/β-catenin and EDAR pathways, respectively) for 5 hours. Statistical significance from control was calculated using a Student t test, (***p < 0.001). (C) qRT-PCR detecting EDAR expression in E6.5 explants cultured with 30 μM CHIR99021 for 5 hours. Statistical significance from control was calculated using a Student t test, (***p < 0.001). (D) From the initial site of primordium formation (arrow), a spreading wave of EDA expression is observed in the developing femoral tracts of chicken embryos. Scale bars: 1 mm. The numerical values for B and C can be found in S10 Data. E, embryonic day; EDA, Ectodysplasin A; EDAR, EDA receptor; qRT-PCR, quantitative reverse transcription PCR. (TIF) [file pbio.3000132.s004.tif]

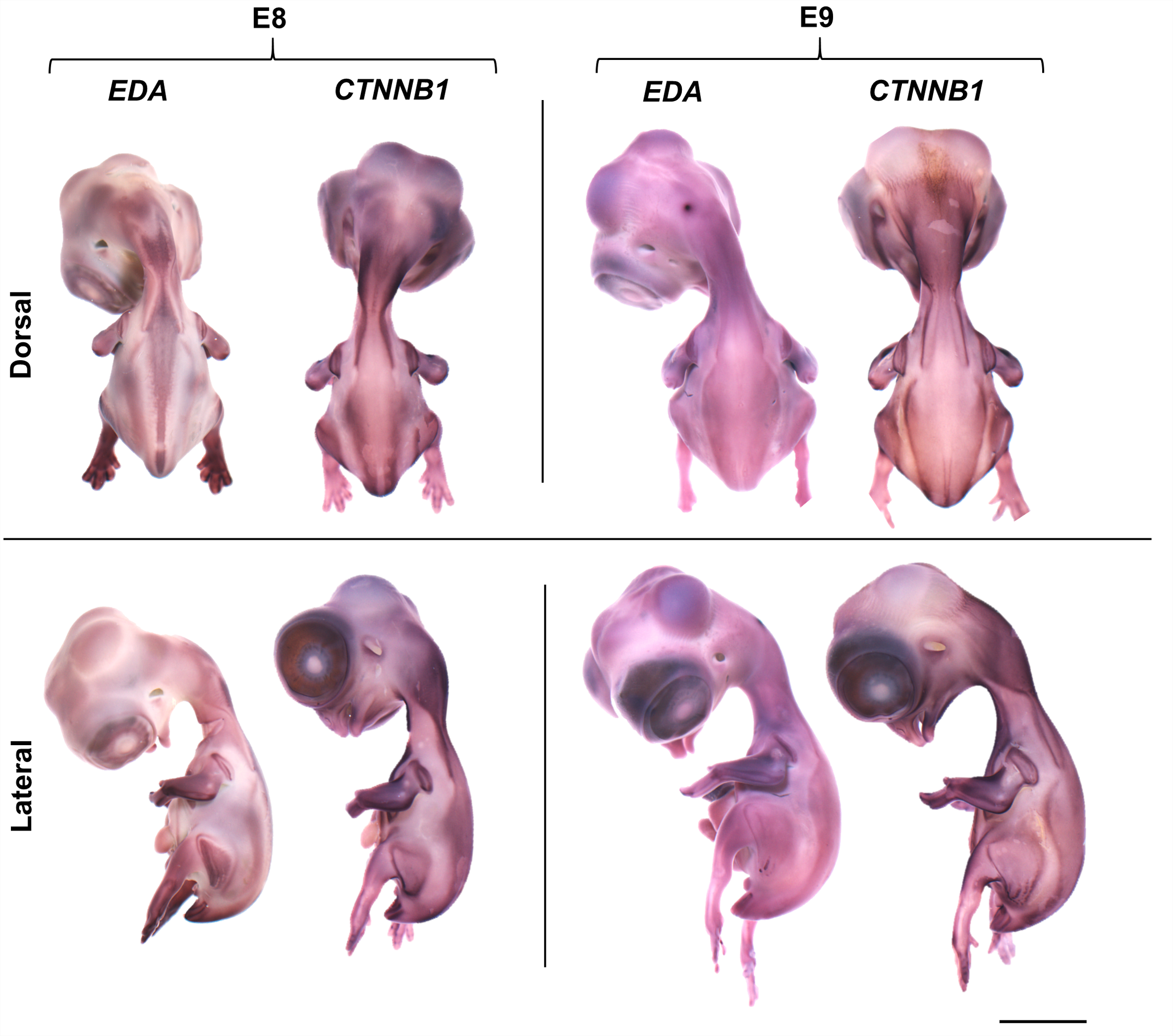

Supplement: S5 Fig — EDA and CTNNB1 expression in E8 and E9 FGF20sc/sc (i.e., scaleless mutant) embryos. The embryos (dorsal and lateral views) exhibit expansion of EDA expression despite the absence of feather primordium formation. CTNNB1 expression becomes restricted to the edges of the presumptive tracts, which have failed to undergo patterning. Scale bar: 5 mm. E, embryonic day. (TIF) [file pbio.3000132.s005.tif]

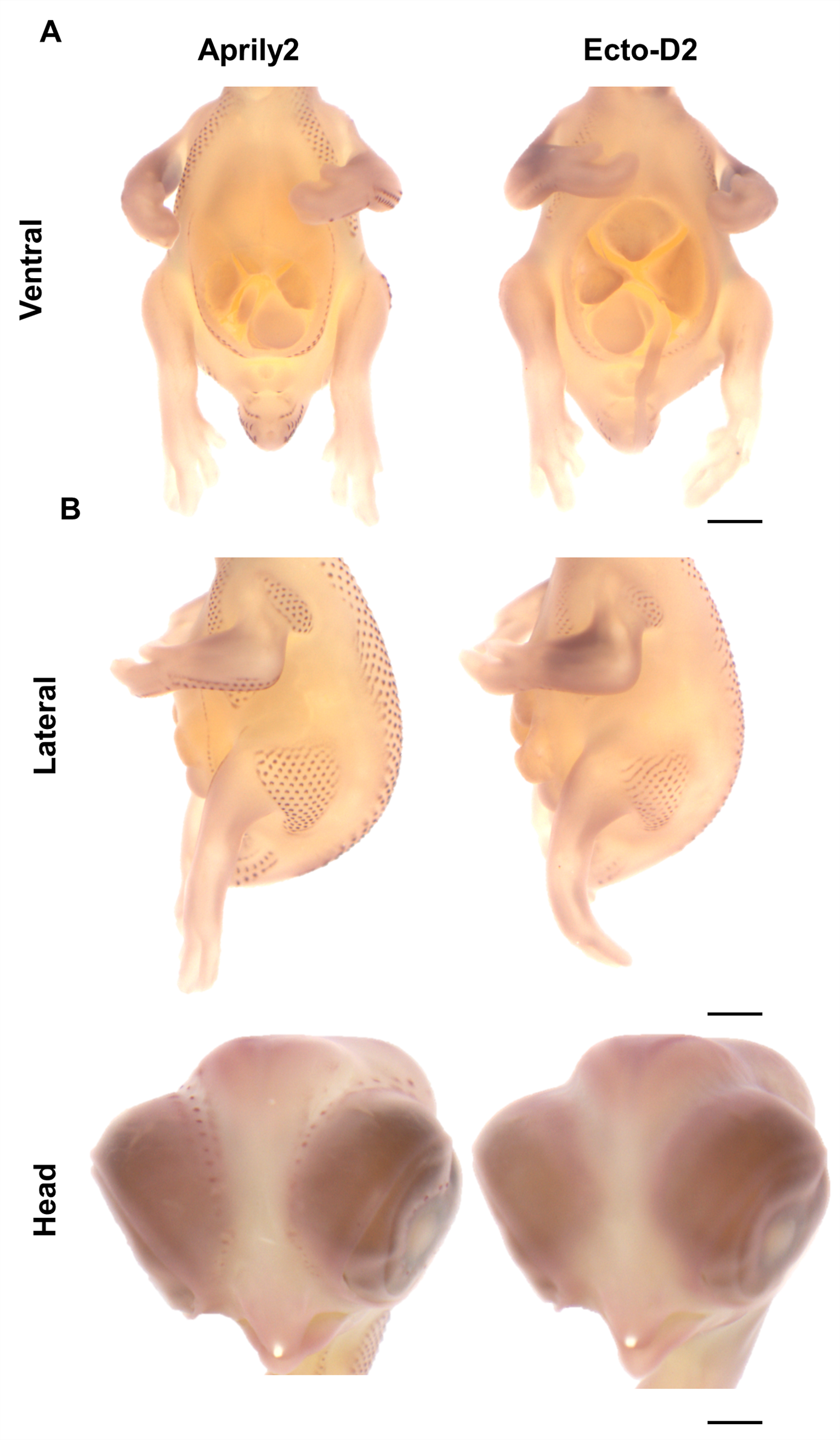

Supplement: S6 Fig — (A) Ventral, (B) lateral, and (C) head views of E8.5 control antibody (Aprily2) and Ecto-D2 injected embryos, treated at E5.5. Inhibition of EDA signalling reduces the extent of primordium formation in every tract compared to controls after 72 hours of treatment. Scale bars: 2 mm. E, embryonic day; EDA, Ectodysplasin A. (TIF) [file pbio.3000132.s006.tif]

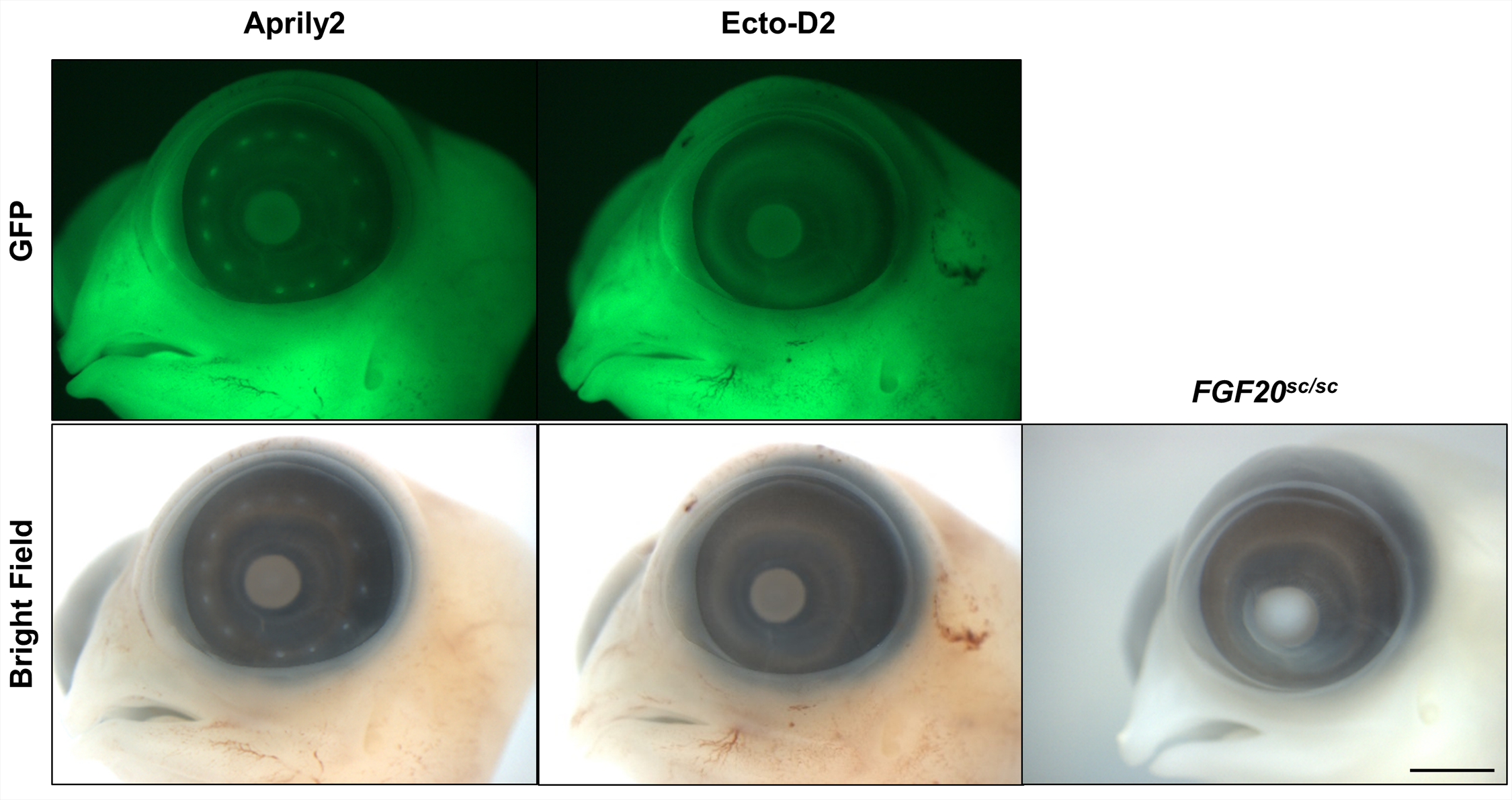

Supplement: S7 Fig — Scleral papillae, visible as a ring of discrete cell condensates within the eye, fail to form in embryos treated in ovo with Ecto-D2 from E5.5 and collected at E8.5. Reduced scleral papilla formation is also observed in developing FGF20sc/sc embryos. Scale bar: 1 mm. E, embryonic day; EDA, Ectodysplasin A. (TIF) [file pbio.3000132.s007.tif]

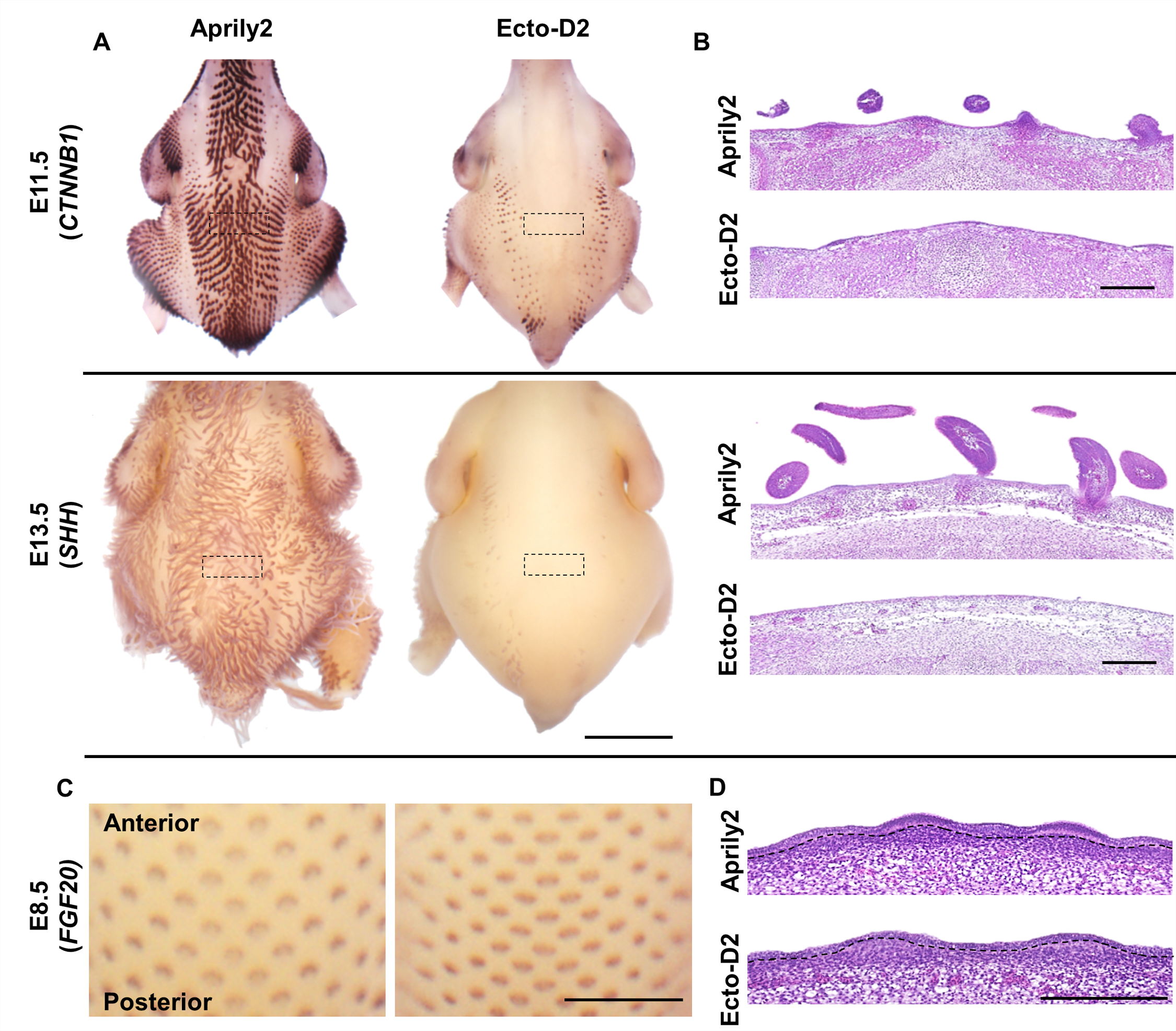

Supplement: S8 Fig — (A) Continuous inhibition of EDA signalling from E5.5 to E11.5 or E13.5 results in the absence of feather formation and almost bare skin, as defined by CTNNB1 and SHH expression. Scale bar: 5 mm. (B) Transverse sections of embryos in (A) reveal loss of mesenchymal condensates in the dorsal tract. Black dotted frame in (A) denotes skin region sectioned. Scale bar: 100 μm. (C) Focalisation of FGF20 expression to the anterior pole within primordia is attenuated at E8.5 when EDA signalling is blocked from E5.5. Scale bar: 1 mm. (D) Mesenchymal condensates are present in E8.5 skin treated in ovo with Ecto-D2 from E5.5. These must regress to yield bare skin at E11.5 and E13.5. Dotted lines demarcate epidermal-dermal junctions. Scale bar: 100 μm. E, embryonic day; EDA, Ectodysplasin A. (TIF) [file pbio.3000132.s008.tif]

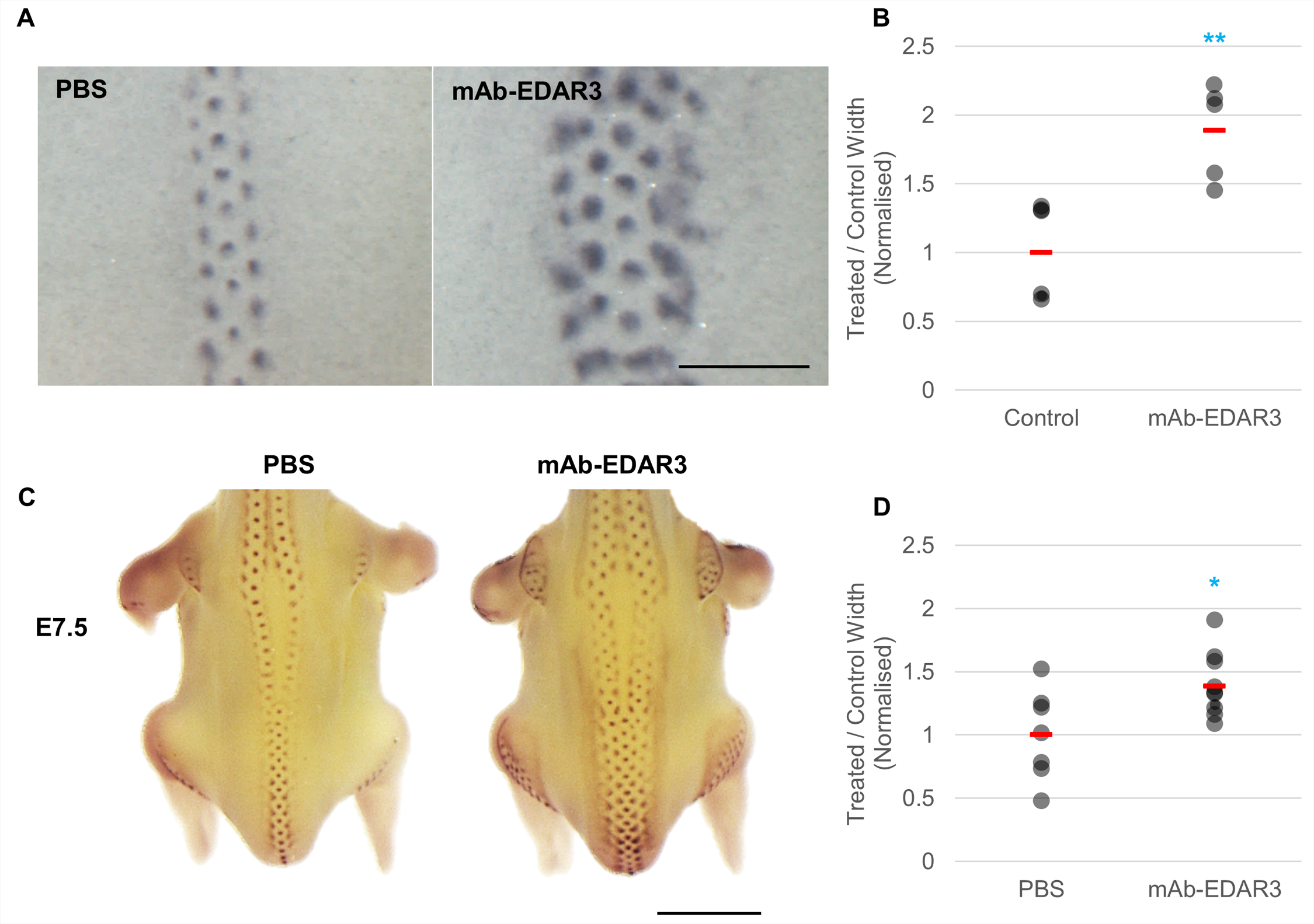

Supplement: S9 Fig — (A) Comparison of the width of the patterned region, defined by FGF20 expression, in E6.5 skin explants cultured for 24 hours in the presence of 2 μg/ml mAb-EDAR3 (an activator of EDAR signalling). (B) Quantification of patterned region width between control and mAb-EDAR3-treated explants. Statistical significance was calculated using a Student t test (**p < 0.01). (C) Width of patterned region, defined by FGF20 expression, is increased in mAb-EDAR3-injected E7.5 embryos compared to control embryos. (D) Quantification of width of primordium generating regions in mAb-EDAR3 in ovo–treated embryos compared to their respective controls. Statistical significance from control was calculated using a Student t test (*p < 0.05). Scale bars: 2 mm. The numerical values for B and D can be found in S11 Data. E, embryonic day; EDAR, Ectodysplasin A receptor. (TIF) [file pbio.3000132.s009.tif]

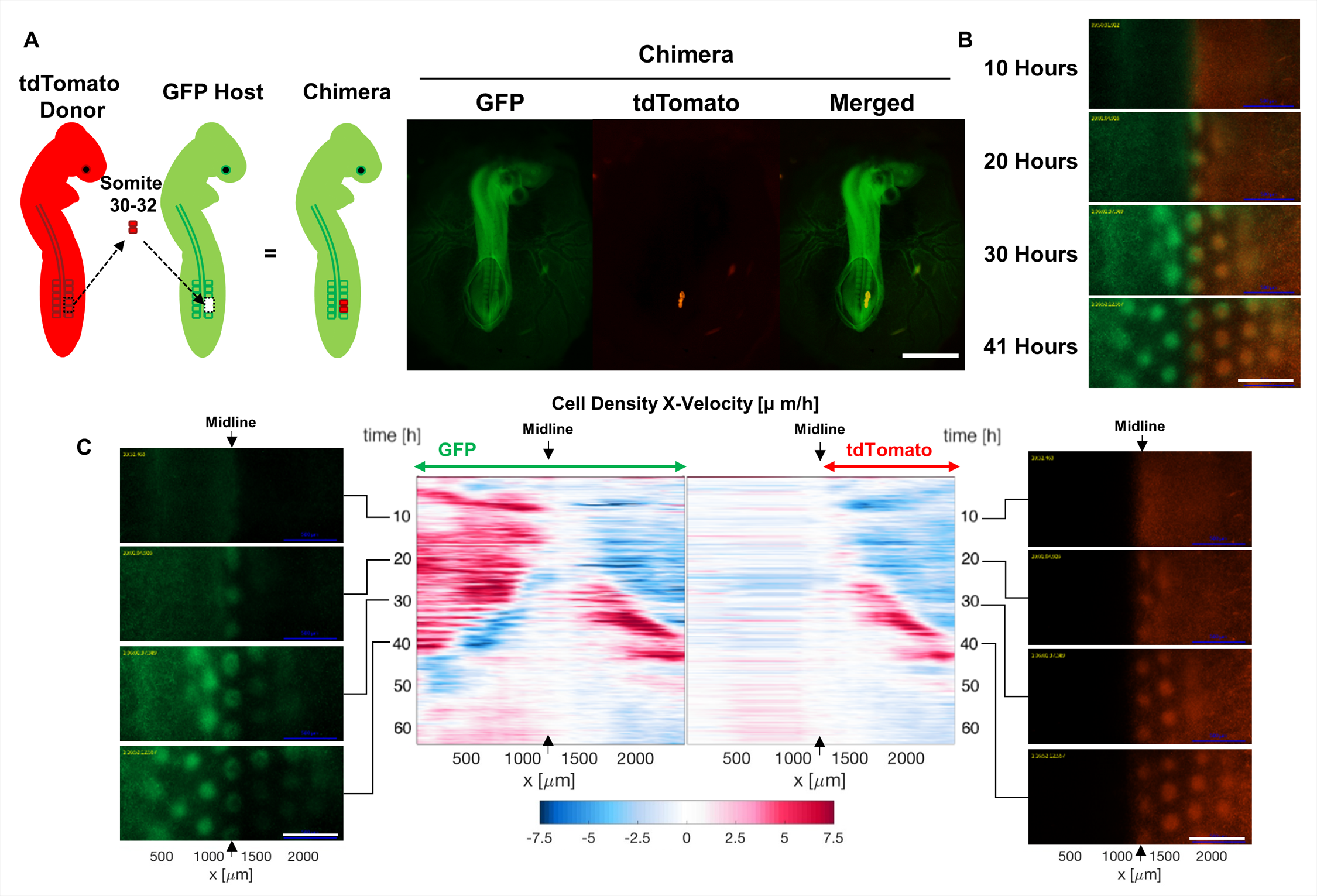

Supplement: S10 Fig — (A) A GFP-tdTomato somite chimeric embryo was generated using a TPZ donor and CAG-GFP host. Somites 30–32 from an HH17 TPZ donor embryo were transplanted to an equivalent region in an HH15 CAG-GFP host, which was then allowed to develop to E6.5. (B) Dorsal skin from the E6.5 chimeric CAG-GFP embryo with transplanted TPZ somites was cultured and imaged in real time. (C) GFP images and tdTomato images obtained from cultured chimeric skin (far left and far right), with X-velocity kymograph (average x-direction speed towards or away from the midline) from each channel (GFP and tdTomato) generated through PIV analysis of the real-time videos. Cell behaviour in the tdTomato half-skin shows the same behaviour as intact CAG-GFP skin, showing that intact skin imaging reflects mesenchymal cell dynamics. Scale bars: 500 μm. E, embryonic day; GFP, green fluorescent protein; HH, Hamburger Hamilton stage; PIV, particle image velocimetry. (TIF) [file pbio.3000132.s010.tif]

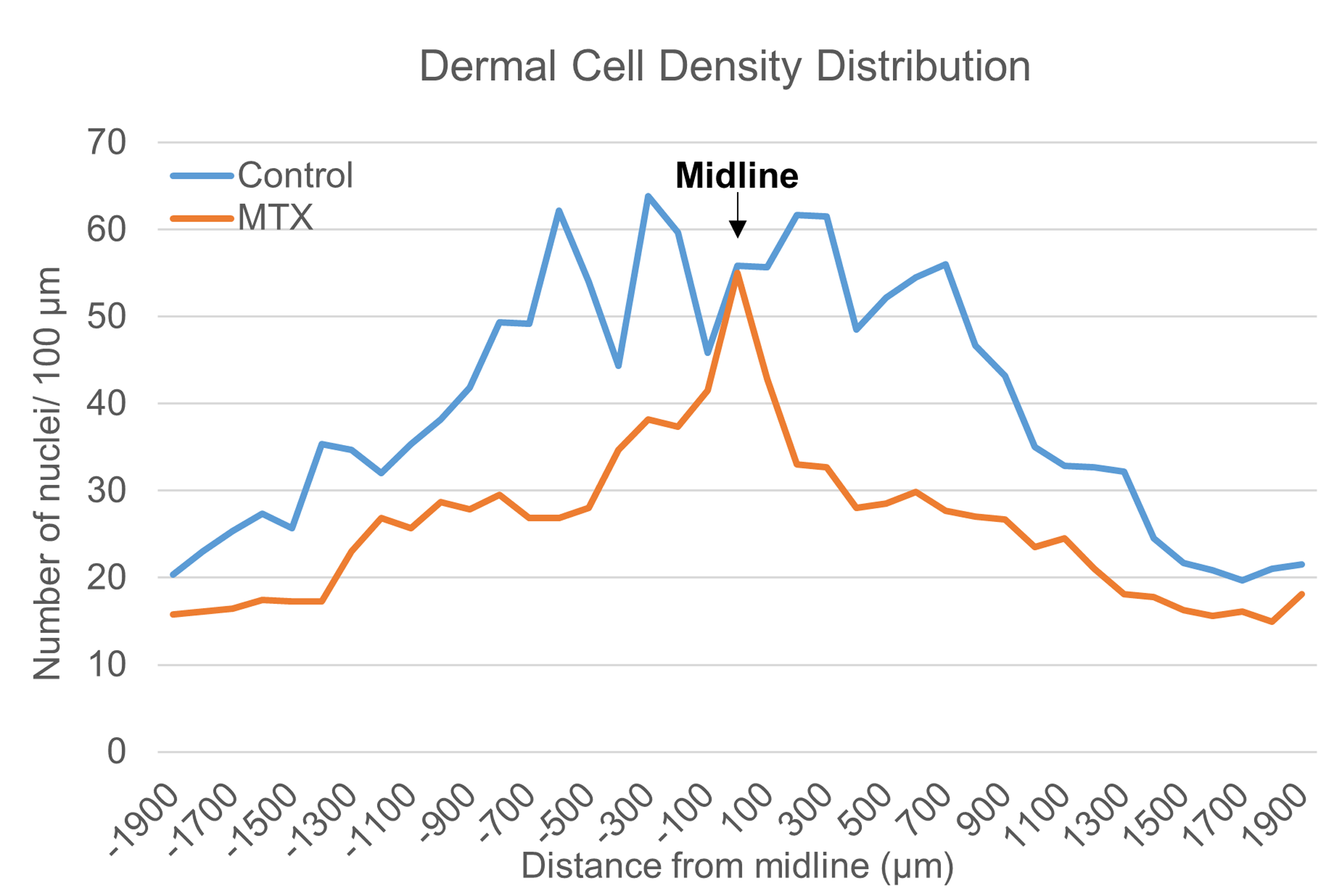

Supplement: S11 Fig — Quantification of mesenchymal cell densities, visualised by DAPI staining of tissue sections, across cultures of E6.5 dorsal skin maintained for 48 hours in the presence or absence of 5 μM MTX, an inhibitor of cell proliferation. The numerical values for the figure can be found in S12 Data. E, embryonic day; MTX, methotrexate. (TIF) [file pbio.3000132.s011.tif]

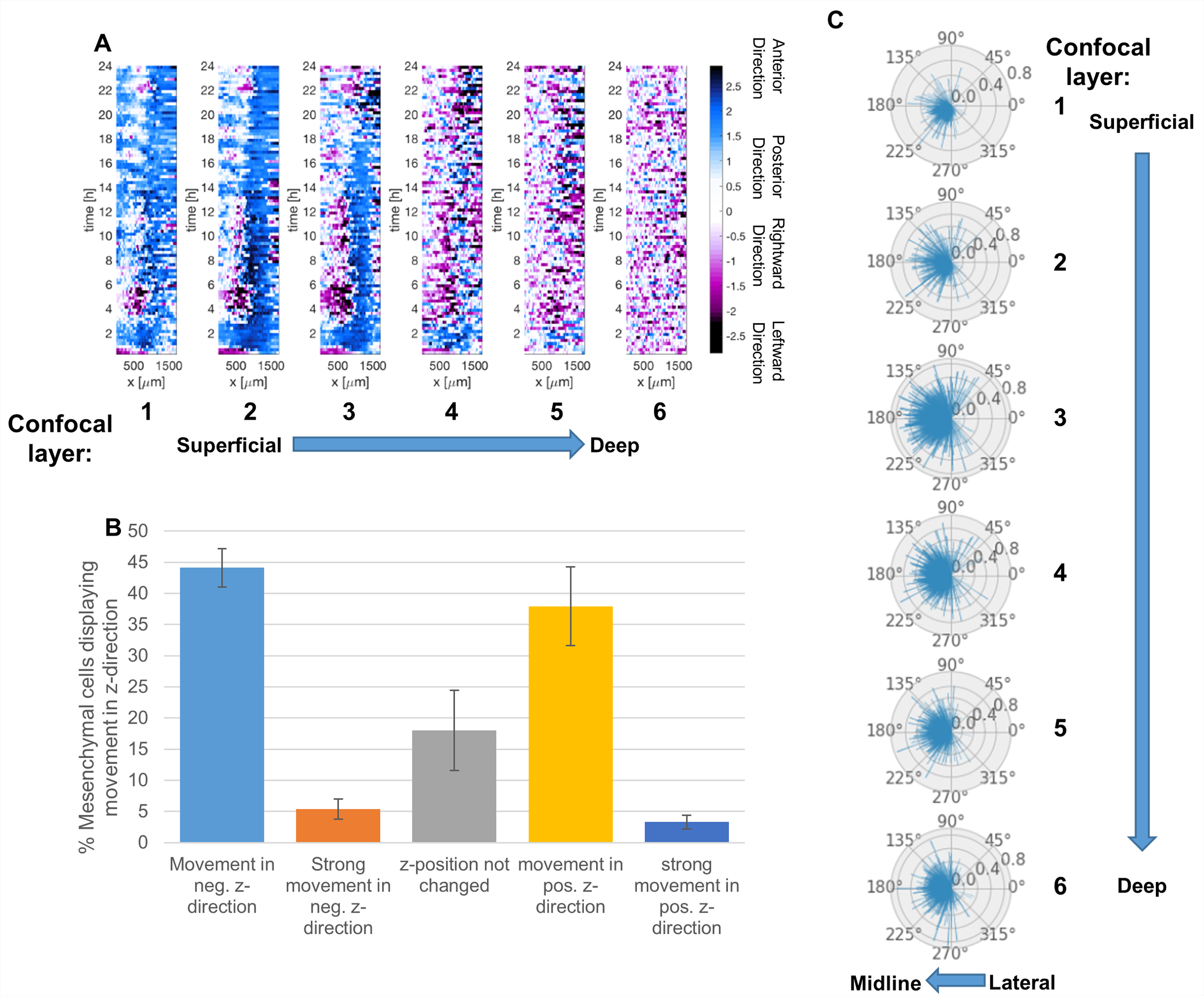

Supplement: S12 Fig — (A) Angle of movement relative to anterior-posterior axis over time calculated for each z-plane optical layer (1: most superficial epidermis; 6: deepest layer mesenchyme) from 2-dimensional single-cell tracking analysis. Cell movement in epithelial layers is more coordinated (indicated by broader blocks of the same colour) and remains constant over greater time periods (indicated by little y-axis colour change) than in the mesenchyme, where mixed colours in deeper layers indicate different directions of cell movement in immediately neighbouring regions. (B) Plot of the percentage of mesenchymal cells moving in a positive (upwards) and a negative (downwards) z-direction. “Strong” movement indicates movement out of one confocal plane and into a neighbouring one (on average 12 μm). Error bars indicate S.E.M. (C) Angle plot showing the time-averaged movement direction (angle) and time-averaged velocity (line length) for every cell in all z-plane layers derived from 2-dimensional cell-tracking analysis for each slice of each of the three time-lapse movies. Medial is to the left and anterior to the top. Individual cell movement occurs almost entirely towards the midline. The numerical values for B and C can be found in S13 Data. (TIF) [file pbio.3000132.s012.tif]

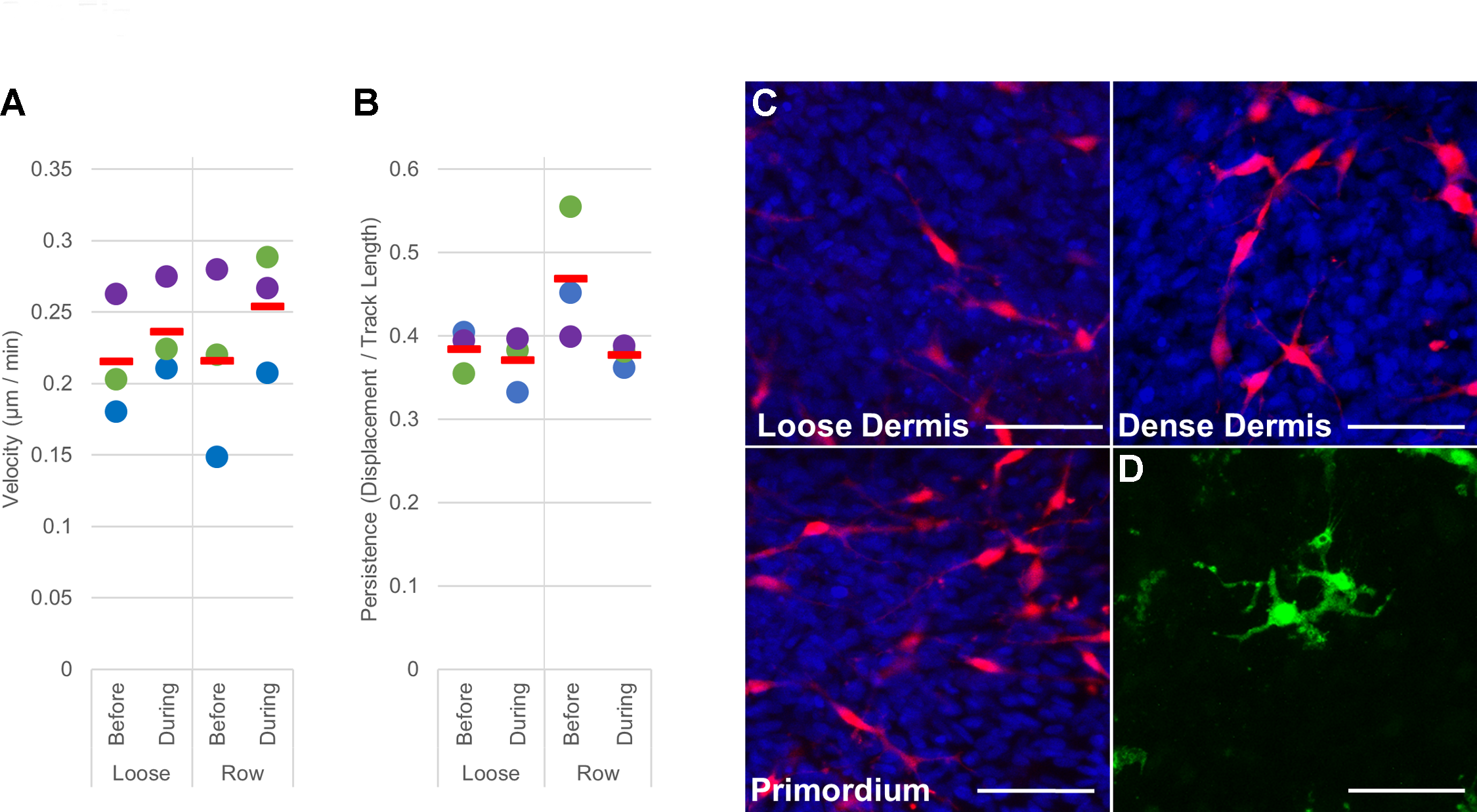

Supplement: S13 Fig — (A) Cell velocity and (B) persistence of mesenchymal cell movement in the 10 hours before and the 10 hours during primordium row formation in the dense mesenchyme (Row) and same time period in the loose mesenchyme (Loose) in TAT-Cre induced Chameleon skin. Persistence of cell movement is highest during the medial migration of cells before initiation of condensate formation along a row. Each coloured circle represents the mean value from a single skin sample. (C) High-resolution images of labelled (red) and unlabelled (Nuclear Blue) Chameleon cells from loose dermis and dense dermis and within a primordium. (D) GFP-expressing macrophages in the skin prepared from a MacGreen reporter chicken display cell morphology that is distinct from that of the labelled Chameleon cells. Scale bars: 50 μm. The numerical values for A and B can be found in S14 Data. GFP, green fluorescent protein. (TIF) [file pbio.3000132.s013.tif]

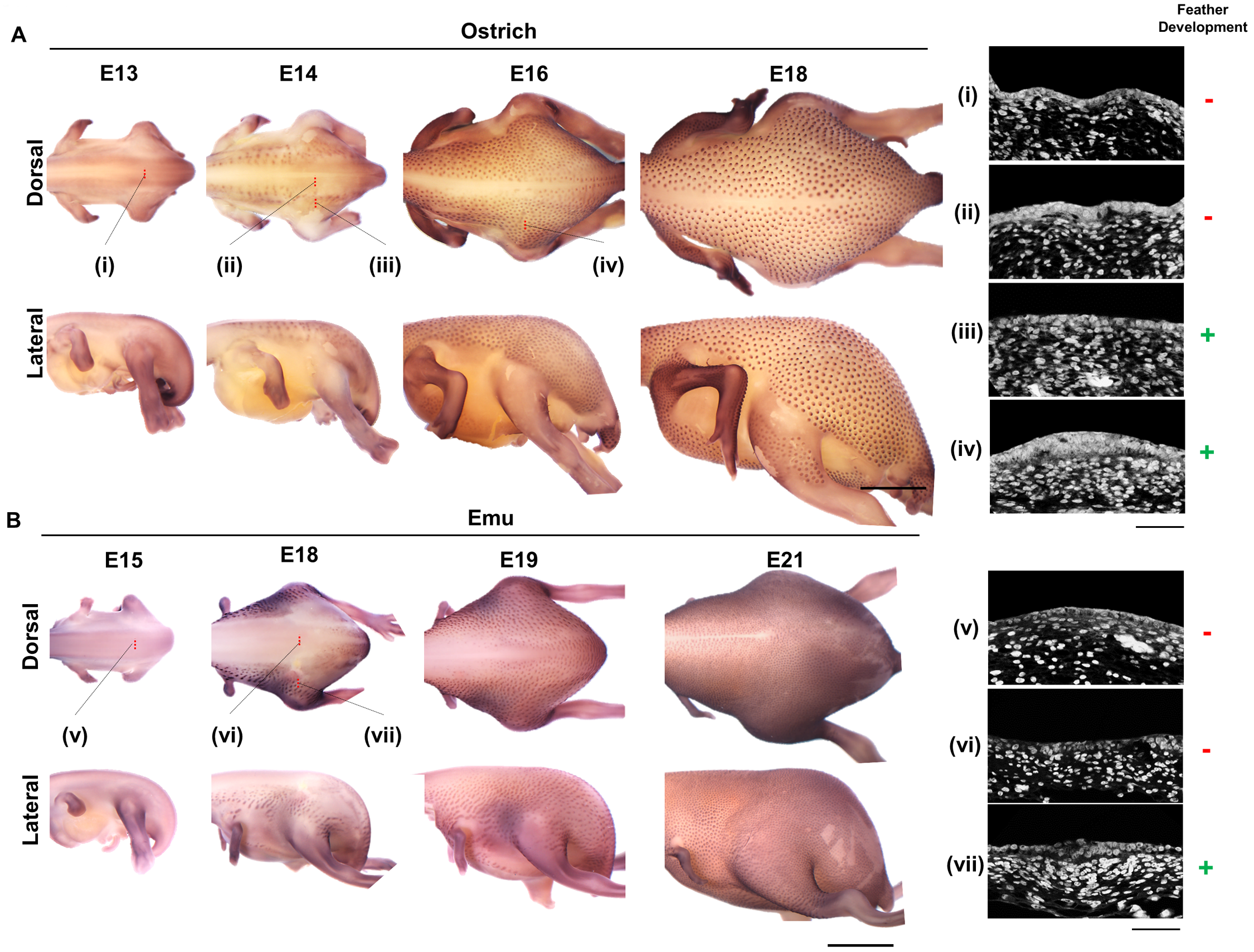

Supplement: S14 Fig — Dorsal and lateral views of primordium pattern formation through in situ hybridisation to detect CTNNB1 transcripts in developing (A) ostrich and (B) emu embryos. Prior to the appearance of primordia, both species display a predefined CTNNB1-expressing dorsal tract; however, a medial-lateral patterning wave is absent. Ostrich embryos pattern within the tract region when and where cell density increases (see corresponding skin sections stained with DAPI to right). Emu embryos fail to attain high mesenchymal cell density while CTNNB1 expression defines the tract, instead producing later feather primordia in lateral regions with high cell density. + indicates skin from a region undergoing feather primordium formation;—indicates skin from a region not undergoing feather primordium formation. Scale bar: 5 mm for embryos; 50 μm for skin sections. (TIF) [file pbio.3000132.s014.tif]

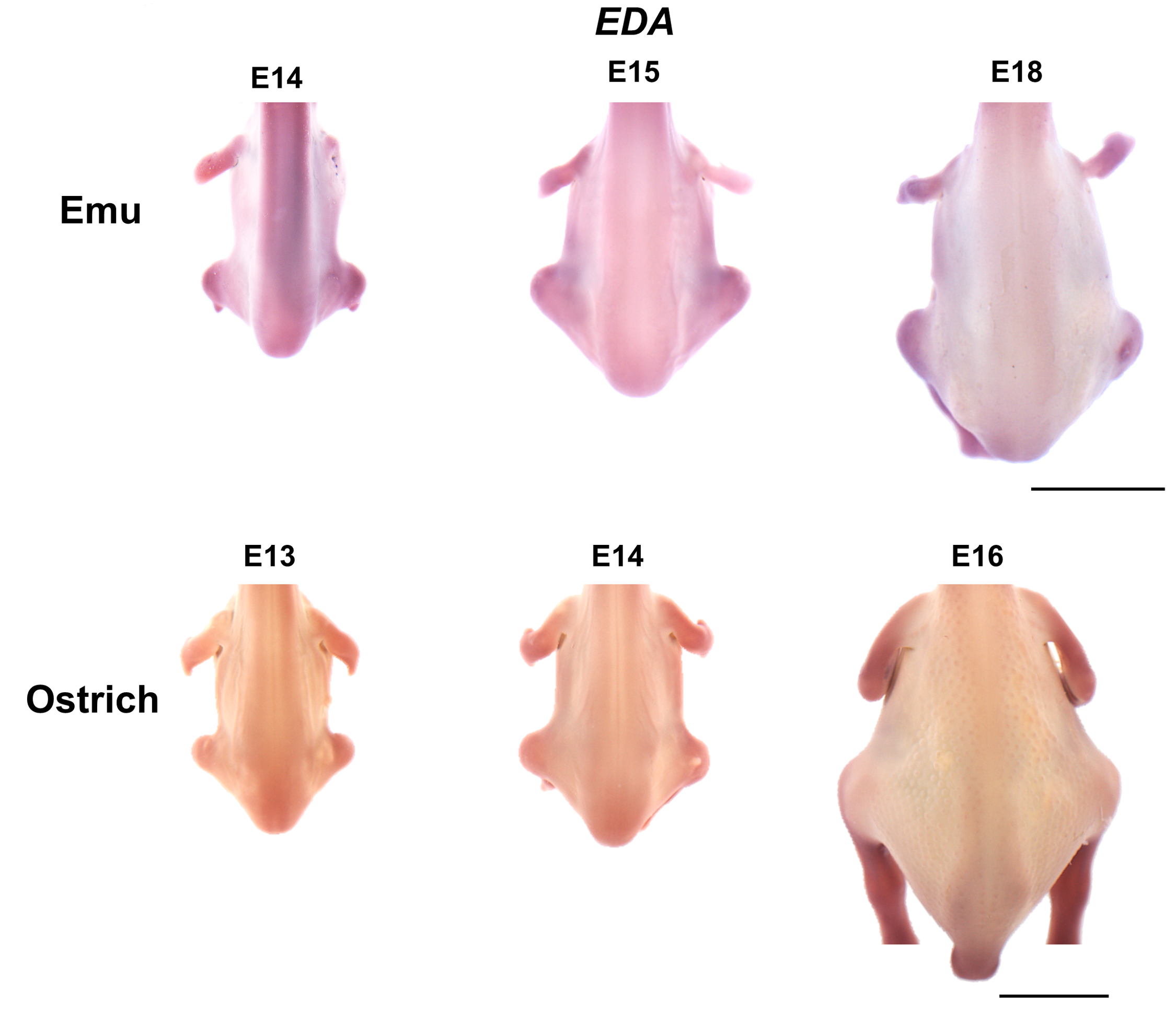

Supplement: S15 Fig — Emu and ostrich embryos express EDA in the dorsal skin corresponding to the tract region, prior to feather formation in both species. Scale bars: 5 mm. (TIF) [file pbio.3000132.s015.tif]

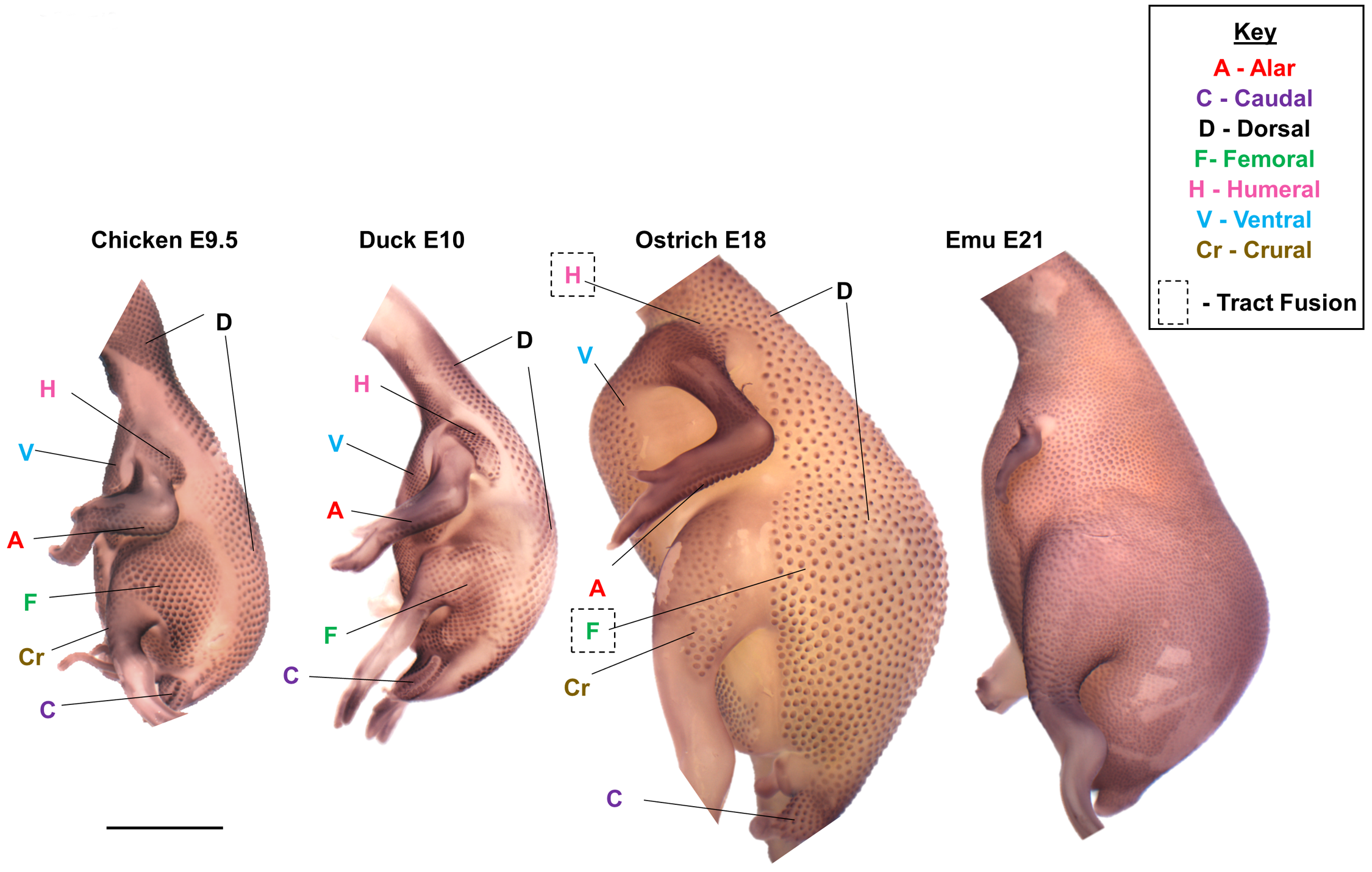

Supplement: S16 Fig — Comparisons of feather distributions between E9.5 chicken, E10 duck, E18 ostrich, and E21 emu. Feather primordia are visualised by detection of the CTNNB1 transcript. Matching feather tracts in chicken, duck, and ostrich can be identified by the presence of the featherless (apteric) regions separating neighbouring feather tracts. However, distinct feather tracts cannot be identified in emu embryos. Instead, almost the entirety of the embryo is covered in small feather primordia. Scale bar: 5 mm. E, embryonic day. (TIF) [file pbio.3000132.s016.tif]

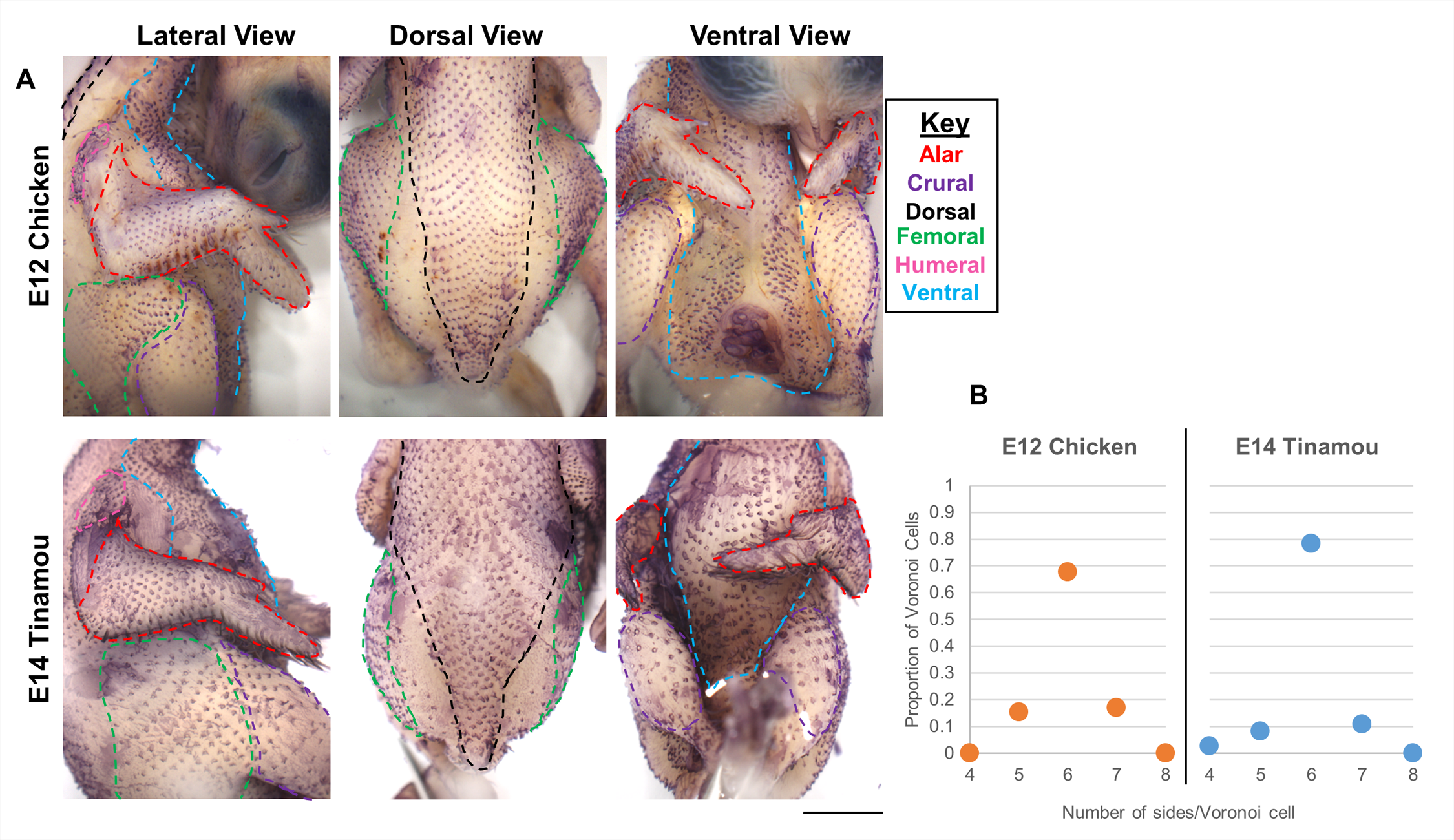

Supplement: S17 Fig — (A) E12 chicken and E14 tinamou embryos were processed for haematoxylin staining to aid visualisation of feather arrangements in the developing embryos. Feather filaments were cut off and embryos dipped in haematoxylin to stain the remaining shaft. Comparisons of the dorsal and lateral sides of the embryos show that the shape of the individual tracts differ between species, but the basic layout of tracts is the same. Scale bar: 5 mm. (B) Voronoi tessellation analysis of images of chicken and tinamou dorsal tracts reveal that tinamou feathers are laid out in a hexagonal lattice that is comparable to the arrangement in chicken. The numerical values for B can be found in S15 Data. E, embryonic day. (TIF) [file pbio.3000132.s017.tif]

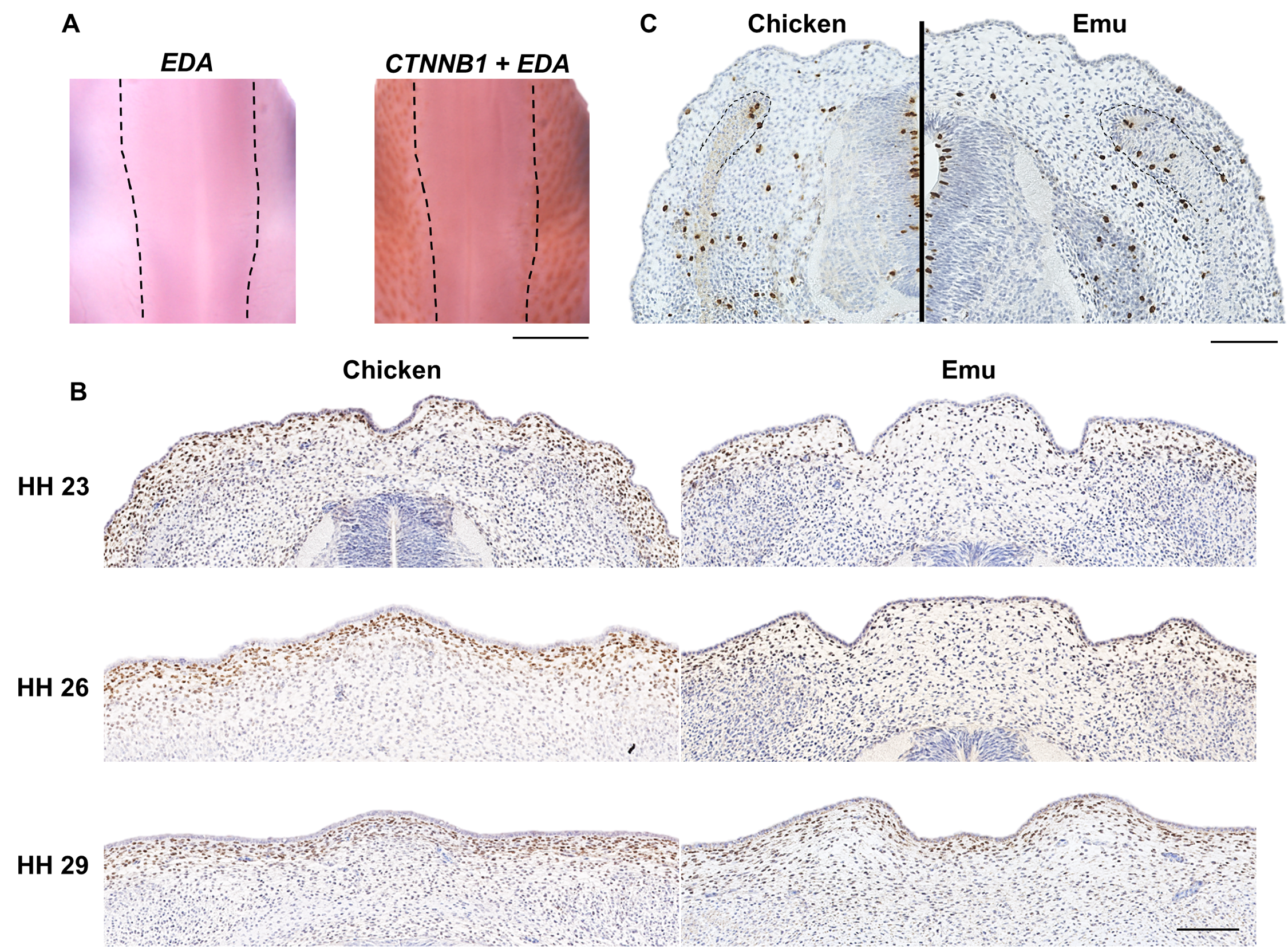

Supplement: S18 Fig — (A) Double in situ hybridisation to detect EDA (purple) and CTNNB1 (orange) transcripts in an E18 emu dorsal tract reveal that primordium formation (CTNNB1 positive foci) is initially absent from regions expressing EDA. Scale bar: 2 mm. (B) Immunohistochemical staining for TWIST1/2 proteins (nuclear marker of mesenchymal cells) in transverse sections of dorsal skin from HH23, HH26, and HH29 chicken and emu embryos. Magnified sections from these images are shown in Fig 8. Scale bar: 100 μm. (C) Immunohistochemical detection of phospho-Histone H3 (Ser10) in transverse sections of dorsal HH21 chicken and emu embryos. Dotted lines denote the dermomyotomal lip. Scale bar: 100 μm. E, embryonic day; HH, Hamburger Hamilton stage. (TIF) [file pbio.3000132.s018.tif]

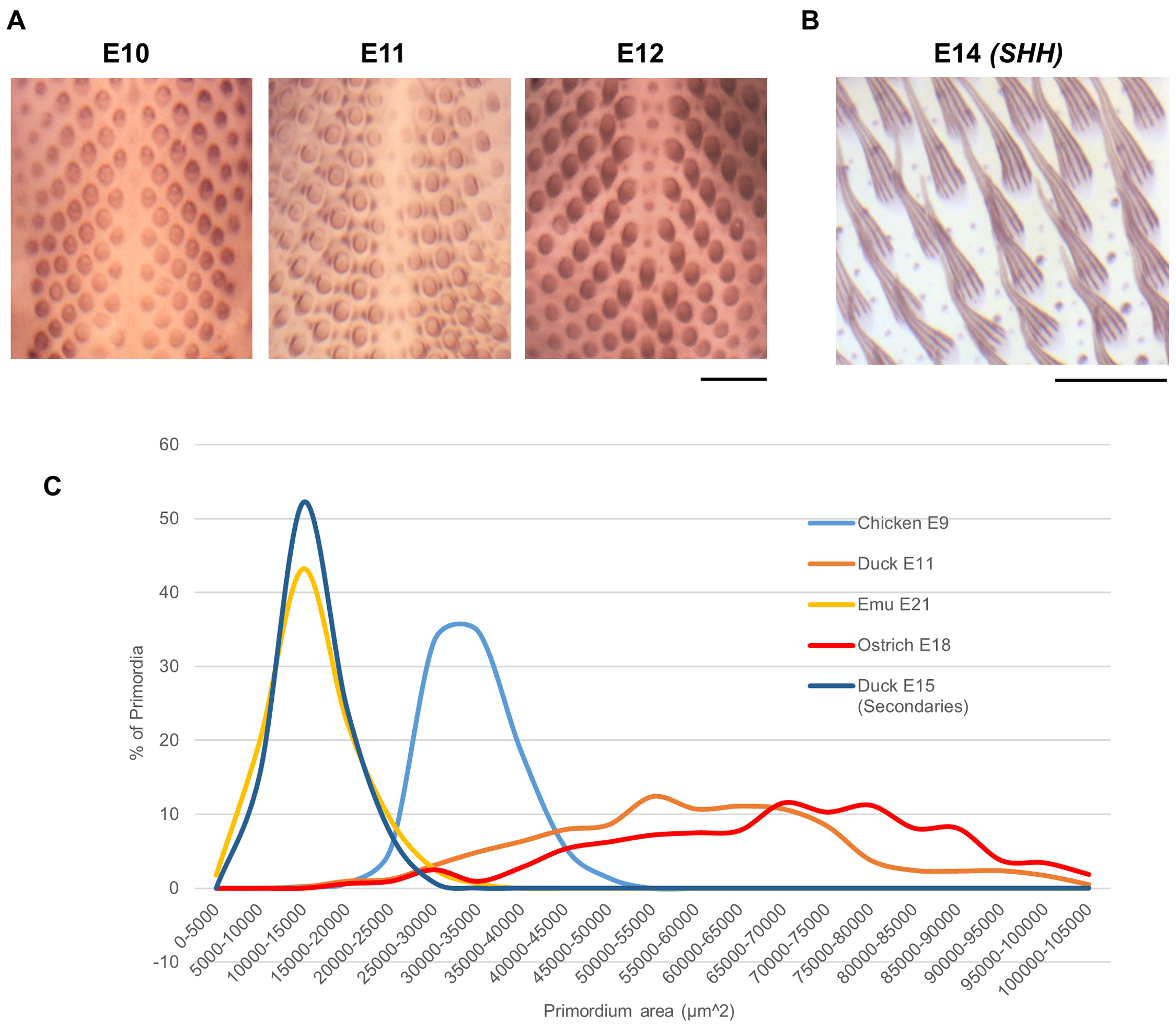

Supplement: S19 Fig — (A) Secondary primordium formation in E10 to E12 duck embryos detected by in situ hybridisation for CTNNB1 expression. At day 10, only the primary feather primordia are visible, with no intervening CTNNB1 expression. At day 11, CTNNB1 expression appears between the existing primary feather primordia. By day 12, secondary feather primordia are resolving between the outgrowing primary feathers. Scale bar: 1 mm. (B) SHH in situ detection of secondary feather primordia, visible as small dots between primary feathers in an E14 duck embryo. The primary feathers at this age each have several stripes of SHH expression related to internal branching of the filament. Scale bar: 1 mm. (C) Primordium size distribution in chicken, duck, emu, and ostrich embryos. Primordium surface areas were grouped into 5,000-μm2 bins, and the percentage of the primordia within each bin was plotted. The numerical values for C can be found in S16 Data. E, embryonic day. (TIF) [file pbio.3000132.s019.tif]
