# Supplementary material for: Feather arrays are patterned by interacting signalling and cell density waves
Source: PLoS Biol. 2019 Feb 21;17(2):e3000132. doi: 10.1371/journal.pbio.3000132 (PMC6383868; doi:10.1371/journal.pbio.3000132)
Supplement: S1 Supporting Methods — (DOCX) [file pbio.3000132.s040.docx]

**Generation of the tdTomato (TPZ) chicken line.**

A CAG-piggyBac transposase (pCyL43) expression plasmid and minimal piggyBac transposon vector (pCyL50) [1] were originally provided by the Wellcome Trust Sanger Institute, and utilised as previously for transposon-mediated stable integration of transgenes in chicken primordial germ cells (PGCs) [[2](#_ENREF_80)]. The enhancer/promoter sequence CAGGS [3], which we have shown drives ubiquitous expression in chicken embryos [[4](#_ENREF_44)], was cloned into the piggyBac vector followed by the coding sequence for the red fluorescent protein tdTomato [[5](#_ENREF_82)]. The transposase expression plasmid and the vector, PBTom, were transfected into PGCs isolated from the transgenic chicken line expressing green fluorescent protein (GFP) [[4](#_ENREF_44)]. After 12 days of propagation in culture the PGCs were sorted by FACS to enrich for cells expressing tdTomato, indicating stable transfection due to transposition of PBTom. The pool of PGCs was further cultured after sorting and pools of approximately 10^5^ cells injected into host embryos at approximately 2.5 days of incubation [[2](#_ENREF_80)]. Six male chicks hatched and survived to sexual maturity. Two of these founder generation males were crossed with stock hens and chicks were visually screened at hatch for expression of GFP and tdTomato. A line was established from a single male that expressed tdTomato alone and embryos produced for the experiments detailed here.

1. **Wang W, Lin C, Lu D, Ning Z, Cox T, et al. (2008) Chromosomal transposition of PiggyBac in mouse embryonic stem cells. Proc Natl Acad Sci U S A 105: 9290-9295.**
2. **Macdonald J, Taylor L, Sherman A, Kawakami K, Takahashi Y, et al. (2012) Efficient genetic modification and germ-line transmission of primordial germ cells using piggyBac and Tol2 transposons. Proc Natl Acad Sci U S A 109: E1466-1472.**
3. **Niwa H, Yamamura K, Miyazaki J (1991) Efficient selection for high-expression transfectants with a novel eukaryotic vector. Gene 108: 193-199.**
4. **McGrew MJ, Sherman A, Lillico SG, Ellard FM, Radcliffe PA, et al. (2008) Localised axial progenitor cell populations in the avian tail bud are not committed to a posterior Hox identity. Development 135: 2289-2299.**
5. **Shaner NC, Campbell RE, Steinbach PA, Giepmans BN, Palmer AE, et al. (2004) Improved monomeric red, orange and yellow fluorescent proteins derived from Discosoma sp. red fluorescent protein. Nature Biotechnology 22: 1567-1572.**
