# Supplementary material for: Feather arrays are patterned by interacting signalling and cell density waves
Source: PLoS Biol. 2019 Feb 21;17(2):e3000132. doi: 10.1371/journal.pbio.3000132 (PMC6383868; doi:10.1371/journal.pbio.3000132)
Supplement: S2 Supporting Methods — (DOCX) [file pbio.3000132.s041.docx]

**Mathematical model for chemotaxis-mediated feather patterning**

We construct a mathematical model to describe the proposed network of cellular and molecular interactions, schematised in Figure 1J. Simulations in 2A are generated using a model first proposed in [1], and we refer there for further details, *in silico* experiments, stability and sensitivity analyses. The simulations in Figure 5G demanded minor modifications in terms of the function choices (allowing comparison / fitting according to the biologically motivated data / aims of the current paper) and the inclusion of cell proliferation kinetics and an initial medial-lateral gradient in cell density. However, the overall structure of the model is the same and based on the schematic in Figure 1J. The brief description here is for the specific model analysed in 5G.

Our mathematical model considers the evolution over space and time of four key variables: dermal cell density, *m*(*x,y,t*); the activated state of the epithelium, *e*(*x,y,t*); FGF concentration, *f*(x*,y,t*); and, BMP concentration, *b*(*x,y,t*). In addition, we consider an imposed “priming wave”, *w*(*x,y,t*), that enhances the patterning capability of the skin. Here, *t* represents time and (*x,y*) defines the spatial position in a two-dimensional rectangular slice of embryonic skin, where *x* represents the anterior-posterior coordinate and *y* is the medial-lateral coordinate. We note that for reasons of model simplicity we have not attempted an explicit description of tissue depth. However, depth is represented implicitly via the variables: the overlying epithelium is represented by its activation state, while dermal cells reside in the underlying mesenchyme. We note that for certain simulations in Figure 5 we exploit the quasi-one-dimensional nature in which patterning starts medially and spreads laterally by eliminating the *x* variable, thereby allowing simulations on a much finer spatial grid.

The model is constructed according to the following set of principal assumptions:

1. Elevated local dermal cell density leads to local epithelium activation, with the mediolaterally-spreading priming wave lowering the density required for activation;

2. Activated epithelium secretes FGF, which diffuses into the mesenchymal layer;

3. FGF induces positive chemotaxis of dermal cells;

4. Clustering of dermal cells triggers their secretion of diffusible BMP;

5. BMP deactivates epithelium.

Mathematically, our model is given by a system of partial-differential equations of reaction-diffusion-advection type, with equations given in the following word-form:

*Rate of variable change* = *Change due to movement* + *Change due to production*/*loss*.

**Priming wave**

An initial wave is assumed to spread through the tissue in medial to lateral fashion, priming the skin for patterning. In the absence of detailed information, we do not model the actual molecular regulation of this process: this represents a challenge more appropriate for future modelling. Rather, we impose a functional form for *w*(*x,y,t*) as follows:

*w*(*x,y,t*) = *ω*_1_(1 + tanh(*ω*_2_(*t* - *y*/*ω*_3_)))/2 [1]

The above generates a “travelling wave” type profile that progressively shifts the skin between an unprimed (*w* = 0) and primed (w = *ω*_1_) state, beginning along the midline and spreading laterally, with speed *ω*_3_, maximum activity *ω*_1_ and “steepness” *ω*_2_. Note that currently we assume any spread along the x-axis (anterior-posterior) is negligible.

**Cell populations**

**Mesenchymal/dermal cell density**

The dynamics of the dermal cells, *m*(*x,y,t*), are given as follows:

*m_t_* = *D_m_*∇^2^*m* - ∇∙(*mχ*(*m, f*)∇*f*) + *g*(*x,y,t*). [2]

The right-hand side terms derive from cellular movement and proliferation: the first term specifies a diffusion-type term, representing an undirected (random) component to dermal cell movement (with associated random motility coefficient, *D_m_*); the second term describes positive chemotaxis of dermal cells up FGF gradients; the final function, *g*(*x,t*), describes cell proliferation. The representation of chemotaxis follows the classical choice of Keller and Segel [2] and subsequently employed in numerous mathematical models for chemotaxis (e.g. see [3]). The function *χ*(*m,f*) is the chemotactic sensitivity. Here we choose *χ*(*m,f*)=*αe*^‑^*^γm^*, where α defines the chemotactic strength coefficient and the *e^-γm^* component limits “overcrowding”: it assumes that chemotactic movement is reduced as the cells become increasingly aggregated.

Cell counts along medial-lateral transects at different stages of bud formation suggest that mean dermal cell density changes spatio-temporally. Initially, dermal cell density is highest along the midline and decreases laterally. Dermal cell densities increase as development proceeds, but such that the average cell density stabilises as clusters form. In line with this, we consider a spreading proliferation wave that gradually reduces the proliferative capacity, via the following *g*(*x,t*):

*g*(*x,y,t*) = *ρ*(1 - *p*(*x,y,t*))*m*(*x,y,t*),

*p*(*x,y,t*) = (1 + tanh(*ω*_2_(*t* - *t** - *y*/*ω*_3_ )))/2,

where *ρ* defines the maximum growth rate. For a specific point along the medial-lateral axis, the above presumes that the cell doubling time is approximately constant from the beginning of the simulation until a time *t** post local passage of the priming wave, following which proliferation slows until negligible. For convenience, we have chosen the parameters that define the shape and speed of the proliferative wave to be identical to those for the priming wave, although this could easily be set otherwise.

**Epithelium activity**

We do not model the epithelial cell population *per se*, rather we consider its overall activity state, defined by *e*(*x,y,t*). In terms of the model, this activity defines the local proportion of epithelium cells that are secreting FGF. Specifically, we assume:

*e_t_* = *k_on_*(*w,m*)(1 - e) - *k_off_*(*b,m*)*e*. [3]

Note that *e*(*x,y,t*) varies between 0 (inactivated) and 1 (activated). The above assumes overall epithelium activity is the balance between activating and inactivating factors, with rates *k_on_*(*w,m*) and *k_off_*(*b,e*). For these two functions we assume:

*k_on_*(*w,c*) = *κ*_1_*q*(*w,m*);

*k_off_*(*b*) = (1 - *q*(*w,m*))(*κ*_2_ + *κ*_3_*b*);

*q*(*w,m*) = *w*∙(*m*/*μ*_1_)*^l^*^1^ + (*m*/*μ*_2_)*^l^*^2^ / 1 + *w* ∙ (*m*/*μ*_1_)*^l^*^1^ + (*m*/*μ*_2_ )*^l^*^2^.

Activation of epithelium is fundamentally linked to the density of underlying dermal cells, such that activity sharply increases above a critical dermal density. In the absence of the priming wave this dermal density is dictated by parameter *μ*_2_. The passage of the priming wave principally serves to lower the dermal density required for activation, via the choice *μ*_1_ < *μ*_2_. Inactivation only occurs in regions of low cell density, at a rate that is enhanced by the inhibitor BMP.

**FGF and BMP extracellular concentrations**

We assume the molecular components FGF and BMP are secreted as diffusible ligands, with dynamics of the following form:

*f_t_* = *D_f_* ∇^2^*f* + *κ_FGF_e* - *δ_FGF_f* ; [4]

*b_t_* = *D_b_* ∇^2^*b* + □(*m*)*m* - *δ_BMP_b* . [5]

The terms on the right-hand side represent molecular diffusion (with diffusion coefficients *D_f,b_*), secretion/production and decay (with decay rates *δ_f,b_*), respectively. The production term for FGF derives from its secretion from the activated epithelium, at a constant rate *κ_FGF_*. BMP is produced by the dermal cells, although we assume that this occurs at a rate that increases with their degree of clustering. Specifically, we consider the Hill function

*h*(*m*) = *κ_BMP_* (*m^l^*^3^ / *μ*_3_*^l3^* + *m^l^*^3^),

where *κ_BMP_* is the maximum production rate, *μ_3_* is the critical clustering density and *l*_3_ is the Hill function coefficient. Note that the choice *μ*_3_ > *μ*_1,2_ dictates that BMP is only produced post-activation/initial clustering.

**Initial/Boundary Conditions**

In line with experimental counts of mesenchymal density along medial-lateral transects, we initially assume a dermal cell density that is highest medially and decreases laterally, taking *m*(*x,y*,0) = *m*_0_*e^-φy^*(1 + *ξ*(*x,y*)): *m*_0_ determines the initial local density of cells along the midline, *φ* denotes the rate of cell density drop-off and *ξ*(*x,y*)∈[-0.01,0.01] defines a small addition of environmental noise. All other variables are initially set at zero. At domain edges we impose zero-flux (no loss) boundary conditions.

**Parameter values**

In the absence of experimental data, our model has relied on a non-dimensional parameter set (below) that fundamentally shows that the proposed mechanism is capable of generating patterns in a manner that qualitatively replicates certain experimentally observed features of feather bud arrangement. Moreover, linear stability analyses and sensitivity analysis of an earlier model [1] imply that the pattern forming capacity of the model is relatively robust to certain parameter variations and, further, recapitulates a variety of experimentally observed pattern changes resulting from targeted perturbations. Nevertheless, it is clearly desirable to move towards a more quantitative test of the model’s capacity through parameter estimation (e.g. diffusion coefficients, proliferation rates, molecular half-lives) to understand whether patterning can be achieved within experimentally observed timescales.

**Symbol Definition Default value**

*D_m_* Dermal cell random motility coefficient 0.01

*D_f_* FGF diffusion coefficient 0.1

*D_b_* BMP diffusion coefficient 1.0

α Dermal cell chemotactic coefficient 5.0

γ Overcrowding limiter 1.5

ρ Maximum dermal cell proliferation rate 0.005

ω_1_ Maximum wave activity 1

ω_2_ Wave steepness 0.2

ω_3_ Wave speed 0.04

κ_1_ Maximum rate of epithelium activation 0.05

κ_2_ BMP-independent epithelium inactivation rate 1

κ_3_ BMP-dependent epithelium inactivation rate 1

μ_1_ Critical dermal density for activation of primed skin 0.5

μ_2_ Critical dermal density for activation of unprimed skin 1.0

μ_3_ Critical dermal density for BMP secretion 2.0

κ*_FGF_* Maximum rate of FGF production 1

κ*_BMP_* Maximum rate of BMP production 1

δ*_FGF_* FGF decay rate 1

δ*_BMP_* BMP decay rate 1

μ_1,2,3_ Hill function coefficients 5

**Simulation Protocols**

Simulations in Figure 5G replicate the impact of perturbations on cell growth and the priming wave on the lateral extent of patterning:

“Normal scenario”, where equations [1-5] are solved with the reference parameter set;

“Block wave”, where we simulate inhibition of the priming wave via setting *w*(*x,y,t*) = 0;

“Block growth”, where we simulate inhibition of cell proliferation via decreasing *ρ* to 0.0025;

“Block both”, where we simultaneously block the priming wave and block cell proliferation

**References:** **Mathematical model for chemotaxis-mediated feather patterning**

[1] Painter, K. J., Ho, W., & Headon, D. J. (2018). A chemotaxis model of feather primordia pattern formation during avian development. Journal of Theoretical Biology, 437, 225-238.

[2] Keller, E. F., & Segel, L. A. (1970). Initiation of slime mold aggregation viewed as an instability. Journal of Theoretical Biology, 26(3), 399-415.

[3] Painter, K. J. (2018). Mathematical models for chemotaxis and their applications in self-organisation phenomena. Journal of Theoretical Biology. Online First. doi.org/10.1016/j.jtbi.2018.06.019
